# KDM6A phosphorylation suppresses PER2 to confer a glycolytic vulnerability in HNSCC

**DOI:** 10.1038/s41419-025-08130-w

**Published:** 2025-11-03

**Authors:** Jun Chen, Yikang Ji, Xin Chen, Mi Zhang, Mei Zhang, Xin Hu, Xing Xu, Yu Zhang, Zhen Zhang, Xinhua Pan, Ming Yan, Jianjun Zhang, Qin Xu, Xi Yang, Wantao Chen, Xu Wang

**Affiliations:** 1 Department of Oral and Maxillofacial-Head and Neck Oncology, Shanghai Ninth People’s Hospital, Shanghai Jiao Tong University School of Medicine, Shanghai, China; 2 National Clinical Research Center for Oral Disease, Shanghai, China; 3 Shanghai Laboratory of Stomatology & Shanghai Research Institute of Stomatology, Shanghai, China

**Keywords:** Oral cancer, Oral cancer

## Abstract

As a key tumor suppressor, KDM6A plays critical roles in maintaining epigenetic homeostasis and suppressing tumorigenesis. However, the regulatory mechanisms controlling KDM6A activity in head and neck squamous cell carcinoma (HNSCC) are not well defined. In this study, we employed tissue microarray analysis of clinical specimens to identify Ser829 as a predominant phosphorylation site of KDM6A in HNSCC and other solid tumors. Using mass spectrometry and biochemical assays, we demonstrate that CDK1-mediated phosphorylation at Ser829 enhances KDM6A binding to SFN, leading to its nuclear export and functional inactivation. Integrated chromatin profiling and metabolic analyses revealed that phosphorylated KDM6A-pSer829 drives glycolytic reprogramming through H3K27Me3-dependent transcriptional silencing of PER2, ultimately promoting tumor growth in vitro and in vivo. These findings establish KDM6A post-translational modification as a pivotal regulator of metabolic adaptation in HNSCC progression, providing a potential therapeutic target for combating cancer through this epigenetic-metabolic axis.

## Introduction

Metabolic reprogramming in cancer, specifically glucose metabolism, is a key feature in oncogenesis [1, 2]. The mitochondrial damage, hypoxia condition and glucose deficiency in cancer cells lead to activation of glycolytic genes, which alter the normal cellular metabolism [3–6]. Glycolysis is considered a late adaptation of rapidly proliferating advanced tumors, yet several descriptive studies indicated that such adaptation may occur early, with increased glycolysis and its branched metabolic pathways [7–9]. Head neck squamous cell carcinoma (HNSCC) exhibits high glycolytic activity [10–12]. Most HNSCC patients are treated with surgery, chemotherapy, and radiotherapy. Targeted agents have been approved for HNSCC treatment, but overall response rates have been moderate. There is an urgent need to explore how HNSCC use intracellular and extracellular signals to promote the glycolysis for developing novel therapeutic strategies.

Histone methylation on some lysine residues is one of the most studied types of epigenetic modification to regulate gene expression [13, 14]. The histone H3 at lysine 27 trimethylation (H3K27Me3) is a transcriptional repressive mark in determining cell fate [15, 16] and metabolic reprogramming [17]. Lysine-specific demethylase 6A (KDM6A), also named as ubiquitously transcribed tetratricopeptide repeat on chromosome X (UTX) [18–22], is the H3K27Me3 site-specific demethylase considered as tumor suppressor in many kinds of tumors. KDM6A is involved in tumor suppression not only via its H3K27Me3 demethylase activity [23, 24], but also via demethylase-independent interactions with other epigenetic complexes. For example, the phase separation of KDM6A is the basis for its chromatin regulatory activity in tumor inhibition [20]. KDM6A was reported as a highly mutated histone demethylase in a survey of different human cancers and cancer cell lines [25]. However, most cancer cells still express the non-mutant KDM6A. Therefore, it is worthwhile to investigate whether there is a post-translational modification to inactivate the wild type of KDM6A.

In the present study, we report that both human and murine HNSCC contain a high density of KDM6A-pSer829 that contribute to tumor growth. Cancer cells export KDM6A from the nucleus through CDK1-triggered phosphorylation at Ser829. This process inhibits the demethylation of H3K27me3, leading to increased glycolytic activity in cancer cells.

## Results

### KDM6A-pSer829 levels are aberrantly high in HNSCC

**Fig. 1:**
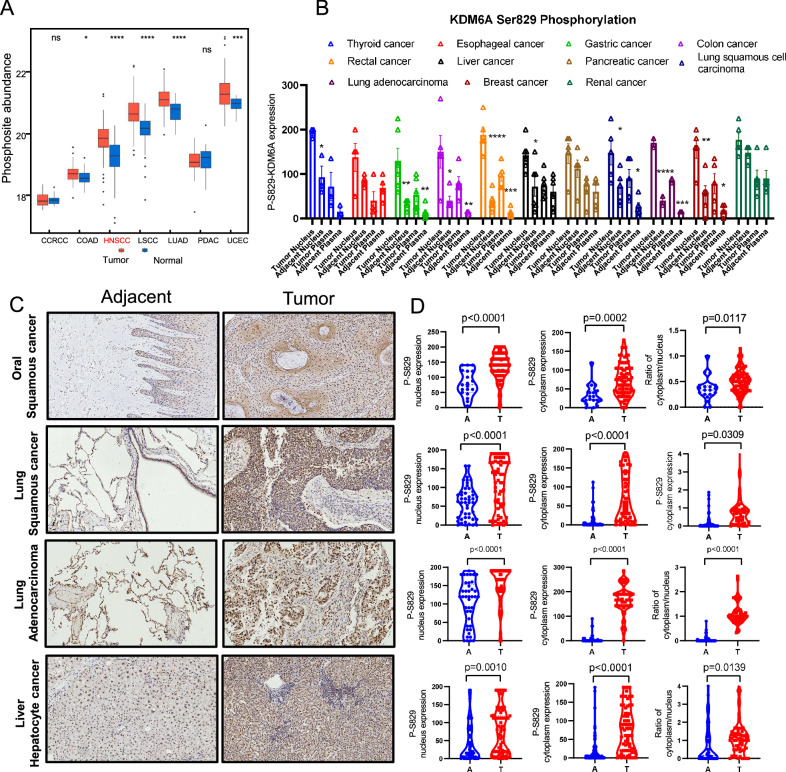
KDM6A-pSer829 levels are aberrantly high in HNSCC. **A** KDM6A-pSer829 abundance in CCRCC, COAD, HNSCC, LSCC, LUAD, PDAC, UCEC, and adjacent tissues from phosophproteomics data in a multiple omics database (http://kb.linkedomics.org). **B** Quantitative analysis of KDM6A-pSer829 levels in nuclei and cytoplasm of section from resected cancer and adjacent tissues. **C** Representative images of IHC assay for KDM6A-pSer829 in Oral squamous cancer, Lung squamous cancer, Lung adenocarcinoma, and Liver hepatocyte cancer. **D** Quantitative analysis of KDM6A-pSer829 levels in nuclei and cytoplasm of section from Oral squamous cancer, Lung squamous cancer, Lung adenocarcinoma, Liver hepatocyte cancer (T), and adjacent tissues (A). Data represent mean ± SEM, unpaired two-tailed *t* test.

Post-translational modifications have been shown to change the function of tumor suppressors in cancer tissues [26]. There are 17 reported KDM6A phosphosites, and we found that only the phosphorylated Ser829 was significantly higher in all examined cancers compared to the corresponding normal tissues when exploring phosophproteomics data in a multiple omics database (http://kb.linkedomics.org) [27] (Fig. 1A). There was no commercially available antibody against KDM6A-pSer829, so we developed a rabbit antibody against KDM6A-pSer829 for immunoblotting and IHC assays (Supplementary Fig. 1A, B).

We performed quantitative analysis of IHC sections of KDM6A-pSer829 levels in nuclei and cytoplasm of section from resected cancer and adjacent tissues. The KDM6A-pSer829 levels in 8 kinds of cancers were significantly higher than in adjacent tissues (Fig. 1B). In a cohort of 80 patients with HNSCC, the KDM6A-pSer829 level was significantly higher in tumors than in adjacent tissues (Fig. 1C, D). We again observed significantly higher of KDM6A-pSer829 levels in tumors than in adjacent tissues in a cohort of Lung Squamous cancer, Lung Adenocarcinoma, and Liver Hepatocyte cancer patients (Fig. 1C, D). Further analysis showed higher abundance of KDM6A-pSer829 in stage III/IV cancer tissues than stage I/II cancer tissues from LSCC (Supplementary Fig. 1C). These data indicate that KDM6A-pS829 levels are elevated in cancer tissue and are closely related to tumor stage.

### KDM6A-pSer829 promotes tumor proliferation

**Fig. 2:**
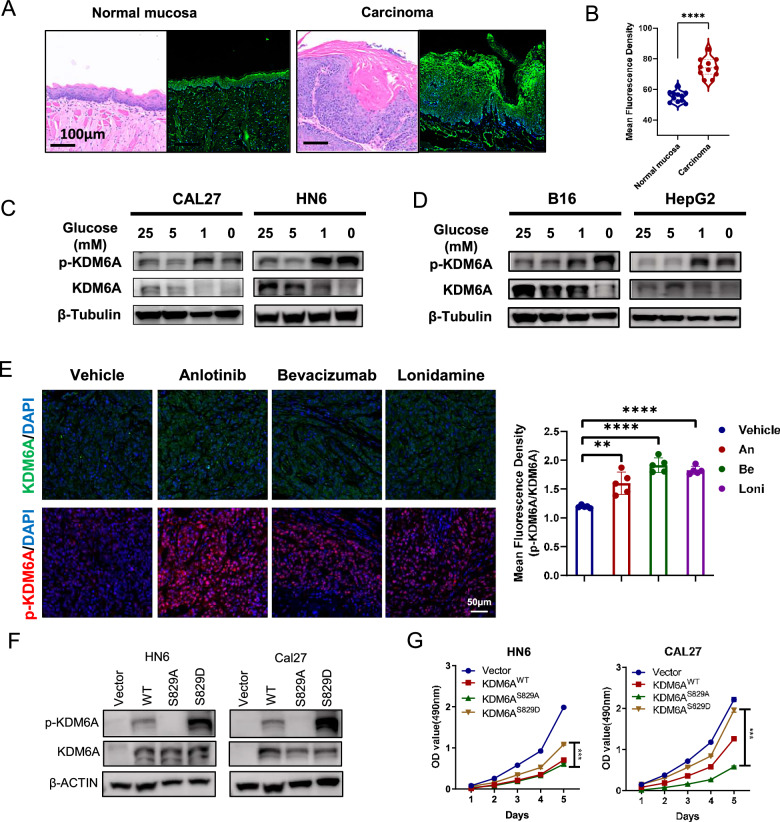
KDM6A-pSer829 promotes tumor proliferation. **A**, **B** Quantitative analysis of KDM6A-pSer829 in normal mucosa and invasive carcinoma. (*n* = 3 per group). **C**, **D** Immunoblotting analysis to examine KDM6A-pSer829 after cells were treated with glucose in the concentration-dependent manner. **E** The immunofluorescent assay to indicate KDM6A-pSer829 and KDM6A levels after the CAL27 xenograft received the indicated hangry therapies. **F** Immunoblotting against p-KDM6A, KDM6A, and β-ACTIN in whole cell lysates from HN6 or CAL27 cells expressing Vector, KDM6A^WT^, KDM6A^S829A^, or KDM6A^S829D^ for 48 h. **G** Proliferation of HN6 and CAL27 cells expressing the Vector, KDM6A^WT^, KDM6A^S829A^, or KDM6A^S829D^ for 1–5 days (*n* = 5 per group). Data represent mean ± SEM. Statistics used unpaired two-tailed *t* test (**B**) and one way ANOVA test (**E** and **G**). Significance is noted as *p < 0.05, ***p < 0.001, ****p < 0.0001.

We also measured KDM6A-pSer829 levels in 4-nitroquinoline 1-oxide (4NQO)-induced murine HNSCC throughout tumorigenesis [28]. The density of KDM6A-pSer829 was significantly higher in invasive carcinoma as compared to normal mucosa (Fig. 2A, B). These results establish that KDM6A-pSer829 levels are aberrantly high in murine HNSCC. Considering that solid tumors exhibit glucose deficiency compared to normal tissues [29], we next performed immunoblotting assay to detect whether glucose deprivation increases KDM6A-pSer829 levels in cancer cells. We found that the glucose deprivation concentration-dependently increased Ser829 phosphorylation of KDM6A in squamous cancer cells HN6 and CAL27 (Fig. 2C and Supplementary Fig. 1D), as well as the B16 melanoma cell and HepG2 hepatocellular carcinoma cell (Fig. 2D and Supplementary Fig. 1E). In vitro, direct glucose deprivation concentration-dependently increased KDM6A-pSer829 levels across multiple cancer cell types. To examine whether altered glucose availability induce KDM6A-pSer829 in vivo, we evaluated two classes of nutrient-starvation therapies: glycolysis inhibitors and angiogenesis inhibitors. To inhibit glycolysis, we used the hexokinase inhibitor in phase II clinical studies, Lonidamine [30]. To inhibit angiogenesis, we used the VEGFR2 inhibitor, Anlotinib [31], and a humanized VEGF antibody, Bevacizumab [32]. We administrated the CAL27 xenograft models with the nutrient-starvation therapies and found that each of the hangry therapies significantly increased KDM6A-pSer829 levels than vehicle group (Fig. 2E). These clinically relevant metabolic interventions significantly elevate tumor KDM6A-pSer829 levels. While employing distinct methodologies, both approaches converge on disrupting glucose metabolism, establishing impaired glucose utilization as a consistent driver of KDM6A phosphorylation in malignant cells.

KDM6A has been reported to function as a tumor suppressor in various cancers. To investigate its intrinsic role in HNSCC, we first performed knockdown experiments and found that knocking down KDM6A significantly enhanced HNSCC cell proliferation (Supplementary Fig. 2A). To evaluate whether KDM6A phosphorylation promotes cell proliferation, we overexpressed phospho-mimic KDM6A^S829D^ and phospho-dead KDM6A^S829A^ in HN6 and CAL27 cells (Fig. 2F and Supplementary Fig. 2B, C). Expressing KDM6A^S829D^ resulted in significant increases in cell proliferation and colony formation compared to cells expressing KDM6A^S829A^, as well as significantly decreased G_1_ phase arrest (Fig. 2G and Supplementary Fig. 2D, E). Thus, KDM6A-pSer829 promotes cell proliferation in HNSCC.

### KDM6A is phosphorylated by CDK1 and transported out of the nucleus

**Fig. 3:**
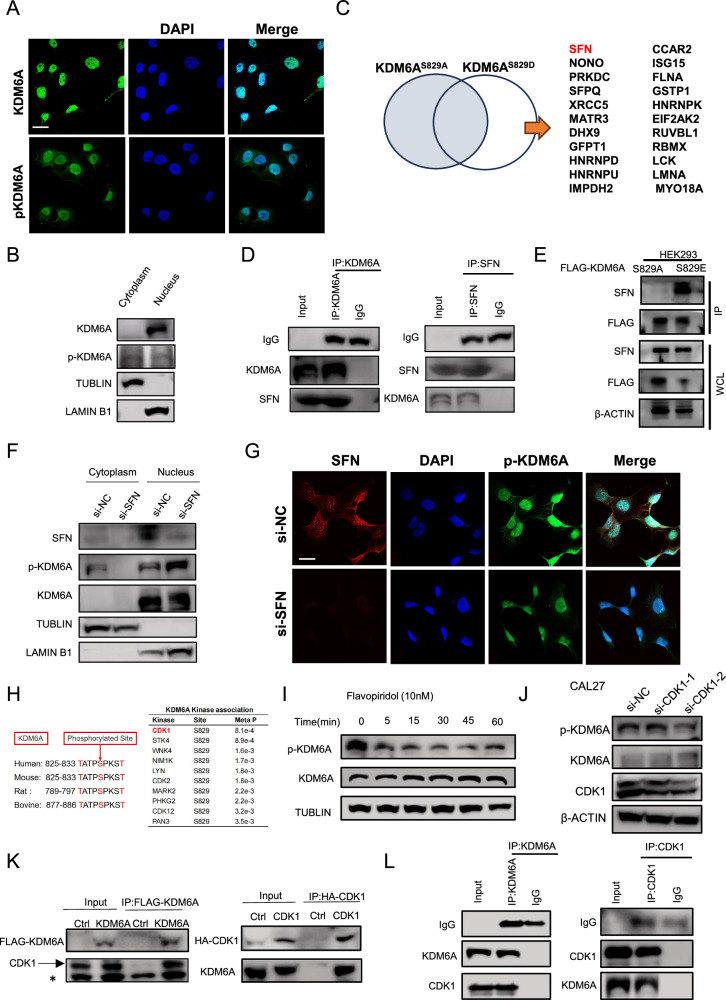
KDM6A is phosphorylated by CDK1 and transported out of the nucleus. **A** Immunostaining against KDM6A, KDM6A-pSer829, and DAPI staining of HN6 cells. Scale bars, 20 μm. **B** Immunoblotting against KDM6A, p-KDM6A, LAMIN B1, and TUBLIN in cytoplasm and nucleus fractions of HN6 cells. **C** Venn diagram showing the numbers of proteins that bind to KDM6A^S829D^ but not to KDM6A^S829A^. **D** Immunoblotting against IgG, KDM6A, and SFN in whole cell lysates and KDM6A-immunoprecipitated eluates, both from HEK293 cells. Right: Immunoblotting against IgG, SFN, and KDM6A in whole cell lysates and SFN-immunoprecipitated eluates, both from HEK293 cells. **E** Immunoblotting against SFN, FLAG-KDM6A, and ACTB in whole cell lysates and FLAG-KDM6A-immunoprecipitated eluates, both from HEK293 cells expressing exogenous KDM6A^S829A^ and KDM6A^S829E^ for 48 h. **F** Immunoblotting against SFN, KDM6A, p-KDM6A, LAMIN B1, and TUBLIN in cytoplasm and nucleus fractions from HN6 cells expressing a control (si-NC) or SFN-targeting (si-SFN) siRNA. **G** Immunostaining against SFN, p-KDM6A, and DAPI staining of HN6 cells expressing a control (si-NC) or SFN-targeting (si-SFN) siRNA. Scale bars, 20 μm. **H** Left: Sequence of amino acid residues for KDM6A phosphorylation in human, mouse, rat, and bovine. Right: Potential kinase associated with KDM6A-Ser829 based on Sequence of amino acid residues. **I** Immunoblotting against p-KDM6A, KDM6A, and TUBLIN in whole cell lysates from HN6 cells treated with the CDK1 inhibitor Flavopiridol (10 nM). **J** Immunoblotting against p-KDM6A, KDM6A, CDK1, and β-ACTIN in whole cell lysates from CAL27 cells expressing a control (si-NC) or CDK1-targeting (si-CDK1) siRNA. **K** Left: Immunoblotting against FLAG-KDM6A and CDK1 in whole cell lysates and FLAG-KDM6A-immunoprecipitated eluates, both from HEK293 cells expressing exogenous CDK1 and FLAG-KDM6A for 48 h. Right: Immunoblotting against HA-CKD1 and KMD6A in whole cell lysates and in HA-CKD1-immunoprecipitated eluates, both from HEK293 cells expressing exogenous KMD6A and HA-CDK1 for 48 h. **L** Immunoblotting against IgG, KDM6A, and CDK1 in whole cell lysates and KDM6A-immunoprecipitated eluates, both from HEK293 cells. Right: Immunoblotting against IgG, CDK1, and KDM6A in whole cell lysates and CDK1-immunoprecipitated eluates, both from HEK293 cells.

Post-translational modification can control a protein’s localization [33]. To examine whether the increased H3K27Me3 in expressing KDM6A^S829D^ cells may reflect the differential nuclear localization of the unmodified & phosphorylated forms of KDM6A, we performed immunocytochemistry (ICC) of HN6 cell. Although KDM6A has been primarily reported as a nuclear protein, our ICC staining demonstrated that HN6 cells exhibited stronger cytoplasmic localization of KDM6A-pSer829 compared to unmodified KDM6A (Fig. 3A and Supplementary Fig. 2F). This observation was further confirmed by nucleocytoplasmic fractionation assays, which revealed substantial accumulation of KDM6A-pSer829 in the cytoplasmic fraction (Fig. 3B). These findings suggest that serine-829 phosphorylation significantly alters the subcellular distribution of KDM6A.

Through systematic protein interaction analysis, we found that KDM6A physically associates with chromatin-modifying enzymes, including KMT2D and EZH2, as well as metabolic regulators such as ATP5F1A and genome maintenance factors like TP53. These interactions functionally connect epigenetic regulation with metabolic control, positioning KDM6A as a molecular bridge between chromatin remodeling and cellular metabolism during tumor development, as supported by our pathway analysis (Supplementary Fig. 3A–E).To identify which protein(s) mediate the translocation of KDM6A-pSer829, we transfected phospho-mimic KDM6A^S829D^, KDM6A^S829E^, and phospho-dead KDM6A^S829A^ into HEK293 cells and performed co-IP and LC/MS assays. In the KDM6A^S829D^ specifically interacting proteins, we focus on Stratifin (SFN), also known as 14-3-3σ (Fig. 3C). SFN has been reported to mediate the translocation of phosphorylated proteins [34, 35]. Co-immunoprecipitation (co-IP) assays revealed a direct interaction between the endogenous KDM6A and SFN (Fig. 3D). Additionally, we found that SFN band more KDM6A^S829E^ than KDM6A^S829A^ with co-IP assay (Fig. 3E). Further supporting SFN-mediated translocation, nucleocytoplasmic fractionation revealed significantly reduced cytoplasmic KDM6A-pSer829 levels in SFN-knockdown cells (Fig. 3F and Supplementary Fig. 3A). Consistent with this finding, immunofluorescence staining demonstrated markedly decreased cytoplasmic localization of KDM6A-pSer829 following SFN depletion (Fig. 3G). These data support that KDM6A-pSer829 was translocated through SFN-mediated export out of nucleus.

Given a previous studying reporting a sequence of XXXSPXXX at the 829 and 830 residues of KDM6A is probably recognized by cyclin-dependent kinase 1 (CDK1) as reported previously (Fig. 3H) [36], we performed kinase assays to test if CDK1 can phosphorylate KDM6A’s Ser829 site. Immunoblotting of extracts from HN6 and CAL27 cells exposed to 3 CDK1 inhibitors (Flavopiridol, Dinaciclib, and Ro-3306) showed time-dependently reductions in the KDM6A-pSer829 level (Fig. 3I and Supplementary Fig. 3B–D). Further, siRNA-mediated knockdown of CDK1 in CAL27 cells resulted in a decreased KDM6A-pSer829 level (Fig. 3J and Supplementary Fig. 3E, F). Additionally, co-immunoprecipitation (co-IP) assays revealed physical interaction between ectopic KDM6A and CDK1 (Fig. 3K), as well as a direct interaction between the endogenous KDM6A and CDK1 (Fig. 3L). These data indicate CDK1 catalyzes the KDM6A-pSer829 modification and results in SFN-mediated export from the nucleus of KDM6A, thus enabling accumulation of H3K27Me3 across the genome.

### KDM6A-pSer829 suppresses PER2 expression via histone modification

**Fig. 4:**
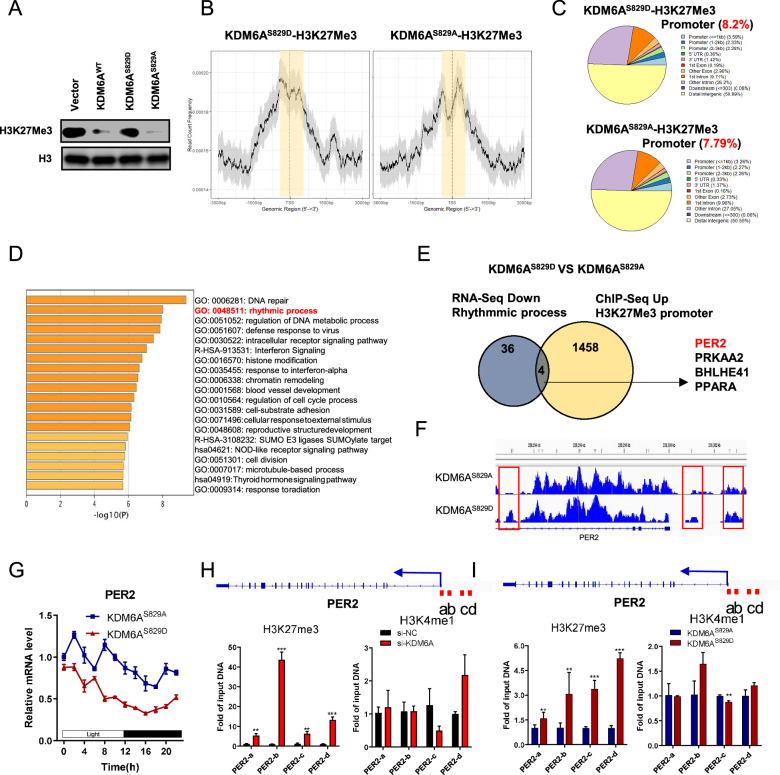
KDM6A-pSer829 suppresses PER2 expression via histone modification. **A** Immunoblotting against H3K27Me3 and H3 in whole cell lysates from HEK293 cells expressing Vector, KDM6A^WT^, KDM6A^S829A^, or KDM6A^S829D^ for 48 h. **B** Peak map of H3K27Me3-associated promoters from ChIP-seq, showing count frequency of genomic region in HN6 cells expressing KDM6A^S829A^ or KDM6A^S829D^. **C** The ratio of H3K27ME3-associated promoters to all promoters in HN6 cells expressing KDM6A^S829A^ or KDM6A^S829D^. **D** GO analysis of differentially expressed genes in HN6 cells expressing KDM6A^S829D^ vs. KDM6A^S829A^ (*n* = 3/3 pooled samples). **E** Venn diagram showing an integrated RNA-seq and ChIP-seq approach harboring increased H3K27Me3 peaks and reduced mRNA expression in CAL27 cells expressing KDM6A^S829D^ compare to KDM6A^S829A^. **F** Genome browser view of normalized ChIP-Seq signals for H3K27Me3 at the *PER2* locus in HN6 cells expressing KDM6A^S829A^ or KDM6A^S829D^. **G** Relative expression of *PER2* at 0, 4, 8, 12, 16, and 24 h in HN6 cells expressing KDM6A^S829A^ or KDM6A^S829D^ (*n* = 3 per group). **H** ChIP-qPCR analysis of H3K27Me3 and H3K4Me1 modifications at *PER2* upon HN6 cells expressing a control (si-NC) or CDK1-targeting (si-CDK1) siRNA (*n* = 3 per group). **I** ChIP-qPCR analysis of H3K27Me3 and H3K4Me1 modifications at *PER2* upon expression of KDM6A^S829A^ or KDM6A^S829D^ in HN6 cells (*n* = 3 per group). Data represent mean ± SEM. Statistics used unpaired two-tailed *t* test (**H**). Significance is noted as *p < 0.05, **p < 0.01, ***p < 0.001.

A previous study reports that loss of demethylase activity, either by chemical inhibition or knock-in of demethylase-dead KDM6A, results in increased H3K27Me3 modification [37]. To examine whether KDM6A-pSer829 changes H3K27Me3 levels in HNSCC, we performed immunoblotting assays of HN6 cells. Expressing phospho-mimic KDM6A^S829D^ in HN6 cells significantly increased the total H3K27Me3 compared to the KDM6A^S829A^ group (Fig. 4A and Supplementary Fig. 3G), indicating the KDM6A-pSer829 decreases the demethylase activity of KDM6A. H3K27Me3 modification in the promoter of genes suppresses transcriptions [38]. To examine whether KDM6A-pSer829 changed the H3K27Me3 modification at the promoter region in the whole genome, we performed ChIP-Seq of HN6 cells transfected with KDM6A^S829A^ or KDM6A^S829D^. The average read count frequency in the transcript start site (TSS) in KDM6A^S829D^ cells was significantly higher than in KDM6A^S829A^ cells (Fig. 4B). The peaks of H3K27Me3 in the promoter region in KDM6A^S829D^ cells are 8.2% and higher than the 7.79% in KDM6A^S829A^ cells (Fig. 4C). These data indicate that phosphorylation at Ser829 site of KDM6A increases H3K27Me3 in HNSCC.

Seeking mechanistic insights into the glycolysis-promoting effects of KDM6A-pSer829 in HNSCC, a GO analysis of HN6 cells showed that expressing KDM6A^S829D^ significantly changed gene expression in rhythmic process pathway compared to expressing KDM6A^S829A^ (Fig. 4D and Supplementary Fig. 4A). An integrated RNA-seq and ChIP-seq approach in rhythmic process pathway showed that four genes mRNA were downregulated with higher H3K27Me3 levels of gene promoters in KDM6A^S829D^ group compared to KDM6A^S829A^ group, including period circadian protein homolog 2 (*PER2*) [39], 5′-AMP-activated protein kinase catalytic subunit alpha-2 (*PRKAA2*) [40], class E basic helix-loop-helix protein 41 (*BHLHE41*) [41] and peroxisome proliferator-activated receptor alpha (*PPARA*) [42] (Fig. 4E), without directly affecting glycolytic pathway genes(*GLUT1*, *ALDOA*, *ENO1*, *PKM2*, *LDHA*, and *MCT1*) (Supplementary Fig. 4B). Among these four genes, PER2 was particularly interesting not only because the H3K27me3 peaks at its promoter region were significantly higher in the OE-KDM6A^S829D^ group compared to the OE-KDM6A^S829A^ group (Fig. 4F), but also for three additional reasons: (1) In HNSCC, we observed significant downregulation of PER2 expression, and patients with low PER2 levels exhibited poorer survival rates (Supplementary Fig. 4C, D); (2) *Per2* knockout in mouse promoted tumorigenesis [39]; (3) *PER2* loss increased glycolysis in cancer cells [43]. The circadian clock gene is typically assessed in terms of its oscillation period, phase, and amplitude. To examine whether KDM6A-pSer829 induced changes in these three clock parameters for *PER2*, we assessed *PER2* expression every 2 h in HN6 cells. Expressing KDM6A^S829D^ in HN6 cells significantly reduced the amplitude of *PER2* mRNA compared to expressing KDM6A^S829A^ (Fig. 4G); this did not affect the period or phase. These data suggest that KDM6A-pSer829 suppresses *PER2* mRNA expression.

KDM6A is a H3K27Me3 histone demethylase, and it is also a component of the COMPASS complex that catalyzes H3K4Me1 to activate target gene expressions [16]. To detect whether the KDM6A-pSer829-suppressed *PER2* was via H3K27Me3 or H3K4Me1 modification, we first performed endogenous KDM6A knockdown followed by ChIP-qPCR analyses using antibodies against H3K27Me3 or H3K4Me1, which revealed reduced H3K27Me3 enrichment at the PER2 promoter while H3K4Me1 levels remained unchanged (Fig. 4H). We then overexpressed phospho-mimic and phospho-dead variants of KDM6A into HN6 cells. ChIP-qPCR analyses with H3K27Me3 antibody showed that increased H3K27Me3 localized to the *PER2* locus in HN6 cells expressing KDM6A^S829D^ (Fig. 4I), suggesting transcriptional inactivation, whereas H3K4Me1 levels were unaffected (Fig. 4I). In other examined circadian clock genes, there were no significant changes of H3K27Me3 or H3K4Me1 localized in the loci, including *PER3*, *CRY1* and *CRY2* (Supplementary Fig. 5A, B). These data suggest that KDM6A-pSer829 suppresses *PER2* expression via increasing the H3K27Me3 levels of *PER2* promoter in HNSCC.

### KDM6A-pSer829 promotes glucose consumption and lactate production

**Fig. 5:**
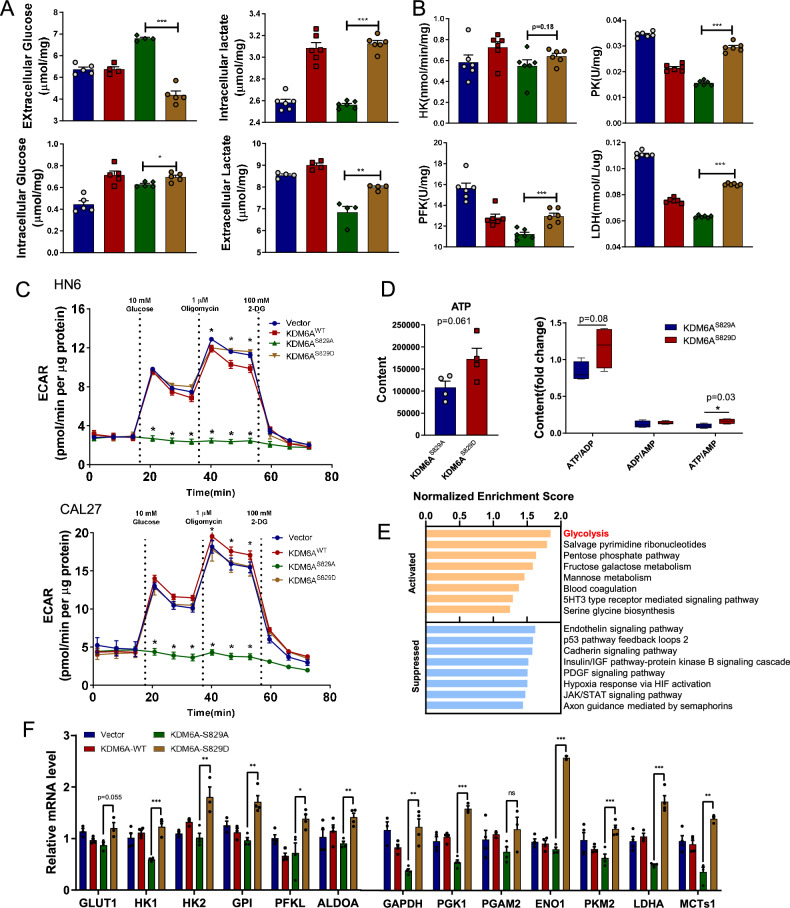
KDM6A-pSer829 promotes glycolysis in HNSCC. **A** Extracellular glucose, extracellular lactate, intracellular glucose, and intracellular lactate in HN6 cells transfected with Vector, KDM6A^WT^, KDM6A^S829A^, or KDM6A^S829D^ for 48 h (*n* = 5 per group). **B** The enzyme activity of HK, PFK, PK, and LDH in HN6 cells transfected with Vector, KDM6A^WT^, KDM6A^S829A^, or KDM6A^S829D^ for 48 h (*n* = 6 per group). **C** ECAR of HN6 and CAL27 cells expressing KDM6A^S829A^ or KDM6A^S829D^ in Seahorse assay (*n* = 5 per group). **D** Relative incorporation of ATP and the ratio of ATP/ADP, ADP/AMP, and ATP/AMP in HN6 cells expressing KDM6A^S829A^ or KDM6A^S829D^ for 48 h (*n* = 4 per group). **E** GSEA analysis of hallmark gene sets from RNA-seq, showing the most significantly enriched gene sets in HN6 cells expressing KDM6A^S829D^ vs. KDM6A^S829A^ (*n* = 3/3 pooled samples). **F** Relative expression of *GLUT1*, *HK1*, *HK2, GPI*, *PFKL*, *ALDOA*, *GAPDH*, *PGK1*, *PGAM2*, *ENO1*, *PKM2*, *LDHA*, and *MCTs1* in HN6 cells transfected with Vector, KDM6A^WT^, KDM6A^S829A^ or KDM6A^S829D^ for 24 h (*n* = 3 per group). Data represent mean ± SEM in (**B** and **D**–**H**). Statistics used unpaired two-tailed *t* test (**A**–**D** and **F**). Significance is noted as *p < 0.05, **p < 0.01, ***p < 0.001.

Both the activation of oncogenes and the loss of tumor suppressors have been shown to induce metabolic reprogramming in various cancers, resulting in enhanced nutrient uptake to supply energetic and biosynthetic pathways [44]. A high rate of lactate production from glucose, despite available oxygen for mitochondrial respiration, is known as a hallmark of rapid cellular proliferation in cancer cells [45]. To examine whether KDM6A-pSer829 elevates lactate production of HNSCC, we expressed phospho-mimic KDM6A^S829D^ and phospho-dead KDM6A^S829A^ in HN6 cells. The intracellular lactate content was significantly increased in the KDM6A^S829D^ group compared to the KDM6A^S829A^ group. The extracellular glucose content was significantly decreased in the KDM6A^S829D^ group compared to the KDM6A^S829A^ group, while the intracellular glucose was significantly increased (Fig. 5A). These data demonstrate that KDM6A-pSer829 promotes the uptake of glucose and production of lactate in HNSCC.

We were interested in the overall glycolytic capacity of these cells, and used the panel of 4 enzymes as representative of glycolytic capacity, including HK, PFK, PKM, and LDH. We examined the catalytic activities of four enzymes regulating glycolysis with in HN6 cells using colorimetric method. The activities of these enzymes were significantly increased in the KDM6A^S829D^ group compared to the KDM6A^S829A^ group (Fig. 5B). A Seahorse analysis showed that the KDM6A^S829D^ group had a significantly increased Extracellular Acidification Rate (ECAR) in both CAL27 and HN6 cells as compared to the corresponding KDM6A^S829A^ groups (Fig. 5C). We collected the cell lysates and measured the ATP, ADP, and AMP content with LC/MS. The KDM6A^S829D^ group had a significantly increased ATP/AMP ratio compared to the KDM6A^S829A^ group (Fig. 5D). These data show that KDM6A-pSer829 promotes the overall glycolytic capacity in HNSCC.

We performed bulk RNA-Seq analysis of HN6 cells transfected with KDM6A^S829A^ or KDM6A^S829D^ and were particularly interested in the potential impacts of the KDM6A-pSer829 on genes with known functions in energy metabolism (Fig. 5E). The mRNA levels of genes encoding glycolytic enzymes were significantly higher in the KDM6A^S829D^ compared to the KDM6A^S829A^ group (Fig. 5F), while knocking down KDM6A in HN6 cells significantly increased the mRNA levels of genes encoding glycolytic enzymes (*p* < 0.05) (Supplementary Fig. 6A). Moreover, GSEA analysis indicated that KDM6A protein levels significantly negatively correlated with glycolysis in HNSCC tissues (Supplementary Fig. 6B). Collectively, these data support that KDM6A-pSer829 promotes cell proliferation and glycolysis in HNSCC.

### Overexpressing PER2 inhibits KDM6A-pSer829-promoted tumor growth

**Fig. 6:**
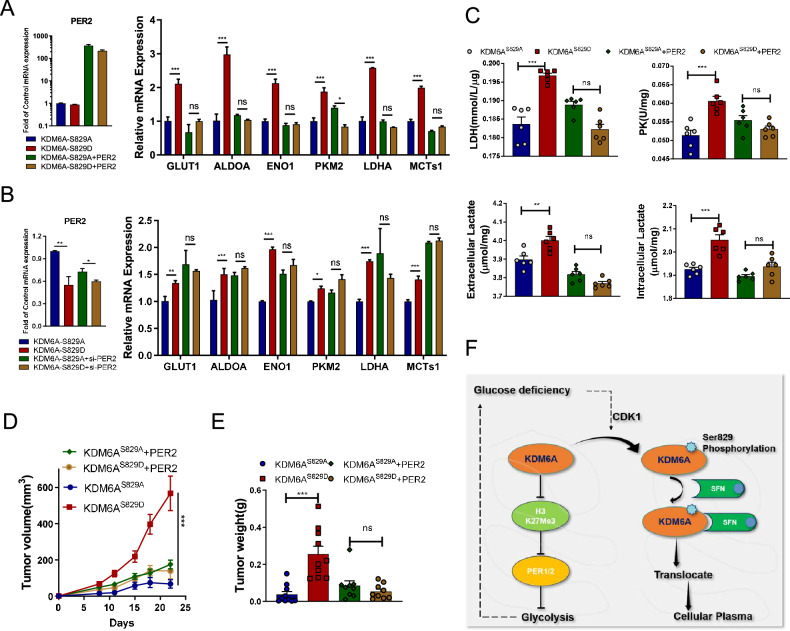
KDM6A-pSer829 promotes glycolysis by suppressing *PER2* mRNA expression. **A** Relative expression of *PER2*, *GLUT1*, *ALDOA*, *ENO1*, *PKM2*, *LDHA*, and *MCTs1* in HN6 cells expressing KDM6A^S829A^, KDM6A^S829D^, KDM6A^S829A^ + PER2 or KDM6A^S829D^ + PER2 for 24 h (*n* = 3 per group). **B** Relative expression of *PER2*, *GLUT1*, *ALDOA*, *ENO1*, *PKM2*, *LDHA*, and *MCTs1* in HN6 cells expressing KDM6A^S829A^, KDM6A^S829D^, KDM6A^S829A^ + si-PER2 or KDM6A^S829D^ + si-PER2 for 24 h (*n* = 3 per group). **C** Upper: The enzyme activity of LDH and PK in HN6 cells expressing KDM6A^S829A^, KDM6A^S829D^, KDM6A^S829A^ + PER2, or KDM6A^S829D^ + PER2 for 48 h (*n* = 6 per group). Lower: Extracellular lactate and intracellular lactate in HN6 cells expressing KDM6A^S829A^, KDM6A^S829D^, KDM6A^S829A^ + PER2, or KDM6A^S829D^ + PER2 for 48 h (*n* = 6 per group). **D**, **E** Volume and weight of HN6 xenografts (*n* = 8 mice per group). **F** Cartoon illustrating the regulation of glycolysis by KDM6A: after phosphorylation by CDK1, KDM6A binds to SFN and is exported out of the nucleus to inhibit PER2 expression through H3K27Me3 mediation and promote glycolysis. Data represent mean ± SEM. Statistics used unpaired two-tailed *t* test (**A**–**E**). Significance is noted as *p < 0.05, **p < 0.01, ***p < 0.001.

PER2 expression is decreased in HNSCC tissues [46], while restoring PER2 expression represses tumor-promoting glycolysis via inactivation several oncogenic glycolytic genes [43]. To examine whether KDM6A-pSer829 increased glycolysis via decreasing PER2 expression, we transfected phospho-mimic KDM6A^S829D^, phospho-dead variants KDM6A^S829A^ and PER2 in HNSCC. Compared to single KDM6A^S829D^, co-overexpressing PER2 with KDM6A^S829D^ suppressed a panel of glycolytic genes as revealed by quantitative PCR experiments, including *GLUT1*, *ALDOA*, *ENO1*, *PKM2*, *LDHA*, and *MCT1* (Fig. 6A). Co-expression of KDM6A^S829D^ with PER2 significantly decreased intracellular glucose and extracellular lactate, and suppressed the activities of PK and LDH when compared to KDM6A^S829D^ (Fig. 6C). Compared to single KDM6A^S829A^, co-transfecting KDM6A^S829A^ with specific siRNAs against *PER2* significantly increased these glycolytic genes, enzyme activity and metabolite (Fig. 6B and Supplementary Fig. 6C, D). These data suggest that cancer cells increase KDM6A-pSer829 and subsequently decrease PER2 levels to promote glycolysis.

To examine whether KDM6A-Ser829-promoted proliferation is mediated by decreasing PER2, we transfected we KDM6A^S829D^, KDM6A^S829A^, and PER2 in HN6 cells. Notably, HN6 cells harboring ectopic PER2 expression significantly inhibited cell proliferation compared to KDM6A^S829D^-expressing counterparts (Supplementary Fig. 6E). Compared to single KDM6A^S829A^, the KDM6A^S829A^ with knocking down PER2 promoted cell proliferation in HN6 cells (Supplementary Fig. 6F). The similar anti-proliferation roles of KDM6A were observed in CAL27 xenograft model. Compared to their KDM6A^S829A^ counterpart, KDM6A^S829D^ tumor displayed faster growth (Fig. 6D, E and Supplementary Fig. 6G). Co-expression of PER2 significantly suppressed the growth of KDM6A^S829D^-transfected tumor (Fig. 6D, E and Supplementary Fig. 6G). These results further support that KDM6A-pSer829 promotes tumor growth via suppressing PER2 expression.

## Discussion

In this study, we aimed to investigate the post-translational modification underlying the regulation of KDM6A-suppressed cell proliferation in HNSCC. Through comprehensive biochemical and functional analyses, we identified that Ser829 phosphorylation of KDM6A as a pivotal regulator of PER2 signaling and glycolysis in solid tumors. Specifically, CDK1-mediated KDM6A-pSer829 promotes SFN-mediated nuclear export and subsequent functional inactivation to erase H3K27Me3. KDM6A-pSer829 suppressed PER2 expression via histone modification, increased glycolytic activity, and enhanced tumor proliferation. Inhibiting KDM6A-pSer829 results in superior antitumor efficacy.

Importantly, while KDM6A mutations are relatively rare in HNSCC [47], we observed significantly elevated KDM6A-pSer829 levels in clinical specimens, suggesting that post-translational modification represents a predominant mechanism of KDM6A regulation in this cancer type [47]. Since KDM6A primarily functions as an H3K27me2/3 demethylase in the nucleus, its exclusion from this compartment likely disrupts its tumor-suppressive role. A particularly intriguing aspect of our findings is the demonstration that KDM6A-pSer829 functions as a sophisticated metabolic sensor. Through detailed kinetic analyses, we revealed that this phosphorylation event exhibits distinct temporal patterns in response to varying glucose concentrations. Under nutrient-deprived conditions, we observed a progressive increase in phosphorylation levels, while high glucose environments induced a non-linear response, suggesting pathway saturation. This dynamic regulation positions KDM6A as a critical molecular integrator that translates metabolic information into epigenetic changes through its H3K27me3 demethylase activity. The biphasic phosphorylation pattern observed at 60 min post-stimulation further indicates the existence of complex regulatory mechanisms, potentially involving kinase reactivation, phosphatase inhibition, or crosstalk with other post-translational modifications [48, 49].

Our mechanistic studies uncovered a crucial link between KDM6A phosphorylation and metabolic reprogramming through the epigenetic regulation of PER2. We demonstrated that pSer829-modified KDM6A exhibits reduced demethylase activity at the PER2 promoter, leading to increased H3K27me3 levels and consequent transcriptional silencing. This finding is particularly significant given PER2’s well-established role as a tumor suppressor that regulates c-Myc expression and maintains metabolic homeostasis [39]. The suppressed PER2 expression drives enhanced glycolytic flux, effectively promoting the Warburg effect characteristic of cancer metabolism [50]. Clinically, our analysis of patient samples revealed a strong correlation between low PER2 expression and poor survival outcomes, highlighting the pathological relevance of this regulatory axis in HNSCC progression and patient prognosis.

The elucidation of this pathway presents multiple promising therapeutic avenues. First, pharmacological inhibition of CDK1 could potentially restore KDM6A’s nuclear localization and tumor suppressive function. Second, strategies to stabilize PER2 expression, possibly through circadian rhythm modulators [51], might counteract the metabolic effects of KDM6A phosphorylation. Third, combination approaches simultaneously targeting both the upstream kinase activity and downstream metabolic consequences could yield synergistic therapeutic benefits [52, 53]. Importantly, our findings suggest that KDM6A-pSer829 levels could serve as a valuable predictive biomarker to identify patients who might benefit most from these targeted interventions. This is particularly relevant for tumors that rely on PTM-mediated rather than genetic KDM6A inactivation, potentially enabling more precise patient stratification and personalized treatment strategies.

While our study establishes a clear connection between KDM6A phosphorylation and metabolic reprogramming, several important questions remain to be addressed. First, the potential crosstalk between phosphorylation and other post-translational modifications of KDM6A, such as acetylation or ubiquitination, warrants further investigation. Second, additional in vivo studies are needed to determine whether PER2 reactivation alone is sufficient to reverse the metabolic effects of KDM6A phosphorylation in tumor models. Third, the broader applicability of this mechanism across different cancer types with varying frequencies of KDM6A mutations and phosphorylation events should be systematically evaluated. Finally, the temporal aspects of this regulation suggest that chronotherapeutic approaches, timed to circadian rhythms, might optimize the efficacy of interventions targeting this pathway. Addressing these questions in future research could significantly enhance our understanding and therapeutic targeting of this important regulatory axis.

In summary, we uncovered a novel CDK1-mediated phosphorylation of KDM6A at Ser829 that connects epigenetic silencing of PER2 and glycolytic activity in HNSCC. These findings not only advance our understanding of non-genetic KDM6A inactivation but also provide a framework for developing targeted therapies against glycolysis in HNSCC (Fig. 6H).

## Methods and Materials

### Cell culture and transfection

HN6 (RRID: CVCL_5516), CAL27 (RRID: CVCL_1107), HEK293T (RRID: CVCL_0063), and SCC7(RRID: CVCL_V412) cell lines were used for this study. HN6 and CAL27 cell lines all originated from male patients of oral squamous cell carcinoma. SCC7 cell lines originated from mouse squamous cell carcinoma. HN6 cell lines were kindly gifted by Professor Mao Li from the University of Maryland. CAL27 and HEK293T cell lines were purchased from the American Type Culture Collection (ATCC, USA). SCC7 cell line was obtained from the Cell Resource Center, Peking Union Medical College (which is part of the National Science and Technology Infrastructure, the National Biomedical Cell-Line Resource, NSTI-BMCR. http://cellresource.cn). HN6, CAL27, and HEK293T were cultured in high-glucose DMEM medium (BasalMedia, China) supplemented with 10% fetal bovine serum (FBS; Cellmax, China) and 1% penicillin-streptomycin (TBD, China). Cells were incubated at 37 °C in an atmosphere of 5% CO_2_. Transfection with polyethylenimine (Polysciences) or Lipofectamine 3000 (Invitrogen) was performed according to the manufacturer’s instructions.

### Antibody generation against S829 phosphorylated KDM6A

Rabbit Polyclonal antibody against KDM6A-pSer829 were generated by Genescript, China. The phosphorylated peptide (STATPpSPKSTEQTTC) conjucted on vector protein and injected into rabbit four times. The antibody was purified by antigen affinity purification.

### Animal models

All animal research was conducted in accordance with the “Guidelines for the Care and Use of Laboratory Animals” (Ministry of Science and Technology of China, 2006) and the corresponding ethical regulations of the hospital. All experimental procedures were approved by the hospital’s Animal Care and Use Committee. All animals lived at room temperature (22–25 °C), with free access to water and food, and were exposed to a 12-h light/dark cycle daily.

Establishment of mouse subcutaneous xenografts model was described in our previous study [54]. Male BALB/c nude mice aged 6–7 weeks were selected in this experiment. After general anesthesia with 1% pentobarbital sodium, the mice were injected with 100 μl cell suspension (5 × 10^5^ cells re-suspended in PBS) was injected into in the right and left flanks of each mouse. Body weights of mice were recorded daily. Tumor volume was calculated using the formula V = (L × W^2^)/2. At the end of the study, mice were sacrificed, and tumors were collected and stored at −80 °C until analysis. For the experiments shown in Fig. 2E, the same number of CAL27 cells were unilaterally injected into male BALB/c nude mice. Treatments (anlotinib 50 mg/kg, bevacizumab 1.5 mg/kg, lonidamine 2 mg/kg) were administered daily for 21 days post-tumor establishment. p-KDM6A and KDM6A levels were quantified by immunofluorescence.

To induce the mouse in-situ carcinoma model, 100 μg/ml 4NQO was added to the drinking water of C57BL/6J mouse in 8-weeks age as previously reported [10]. When the weight of the mouse began to decline (about 16 weeks), the 4NQO water was replaced by normal water. The mouse was observed three times a week until the lesions were detectable on the tongues (about 16 weeks). The mouse was anesthetized and underwent heart perfusion with 4% paraformaldehyde, and then the entire tongue was harvested. The histological stages of the lesions were determined by H&E staining.

### RNA-sequencing (RNA-seq) experiment and analysis

RNA-seq assay was performed by OEBiotech China. Total RNA was extracted from HN6 cells overexpressing different KDM6A mutants, and a cDNA library was prepared according to the standard Illumina RNA-seq instructions. Hisat2 was selected as the mapping tool because Hisat2 can generate a database of splice junctions based on the gene model annotation file. FeatureCounts v1.5.0-p3 was used to count the read numbers mapped to each gene. A fold change > 1.5 and false discovery rate (FDR) < 0.05 were set as the thresholds for identifying the differentially expressed genes (DEGs). Gene Ontology (GO) enrichment analysis and Kyoto Encyclopaedia of Genes and Genomes (KEGG) analysis of differentially expressed genes were performed using the “clusterProfiler” R package, and a cDNA library was prepared according to the standard Illumina RNA-seq instructions. Hisat2 was selected as the mapping tool because Hisat2 can generate a database of splice junctions based on the gene model annotation file. FeatureCounts v1.5.0-p3 was used to count the read numbers mapped to each gene. A fold change > 1.5 and false discovery rate (FDR) < 0.05 were set as the thresholds for identifying the DEGs. GO enrichment analysis and KEGG analysis of differentially expressed genes were performed using the “clusterProfiler” R package.

### Enzyme activity and metabolite concentration

To detect metabolic enzyme activity and metabolite content, 5 × 10^4^ cells were cultured and treated according to instructions, and then the metabolic enzyme activity was analyzed using a specific detection kit according to the manufacturer’s instructions. The list of all kits used is as follows: hexokinase (HK) activity assay kit (Nanjing Jiancheng Bioengineering Institute, A077-3-1), phosphofructokinase (PFK) activity assay kit (Nanjing Jiancheng Bioengineering Institute, A129-1-1), Pyruvate kinase (PK) activity assay kit (Nanjing Jiancheng Bioengineering Institute, A076-1-1), lactate dehydrogenase (LDH) activity assay kit (Nanjing Jiancheng Bioengineering Institute, A020-2-2), glucose detection kit (Nanjing Jiancheng Bioengineering Institute, F006-1-1) and lactic acid detection kit (Nanjing Jiancheng Bioengineering Institute, A019-2-1). These values are normalized to protein concentrations.

### Antibodies and Nucleic acid transfection

Antibodies used in this study are listed in Supplementary Table S1. The KDM6A-Ser829 antibody was generated by Genscript, China. KDM6A (WT, S829A, S829D, S829E), CDK1, PER1 and PER2 were separately constructed into pCMV3 vectors. Cells were transfected with different Nucleic acid fragment or expression vectors using Lipofectamine 3000 (Life Technologies).

The si-RNA transfection was performed when the cell density reached 60%. The cells were starved under serum-free medium for 4 h, and siRNA (final transfection concentration: 20 nM) and Lipo3000 were diluted in OPTI-MEM, respectively. The diluted siRNA and Lipo300 were mixed and left at room temperature for 5 min before being added to the cells. After 4 h, the cells were replaced with serum-containing medium. The siRNA sequences were listed in Supplementary Table S1.

### Nuclear and cytoplasmic protein extraction

Prepare reagents by thawing kit (P0027, Beyotime) components on ice and supplementing cytoplasmic/nuclear extraction buffers with PMSF (1 mM final). For adherent cells, wash with PBS, harvest by scraping, and pellet by centrifugation. Lyse cells in cytoplasmic extraction buffer A, vortex 5 s, then incubate 10–15 min on ice. Add 10 μL buffer B, vortex 5 s, incubate 1 min on ice, then centrifuge at 12,000–16,000 × *g* for 5 min. Collect supernatant (cytoplasmic fraction). Resuspend pellet in 50 μL nuclear extraction buffer, vortex 15–30 s, and incubate on ice for 30 min with intermittent vortexing. Centrifuge at 12,000–16,000 × *g* for 10 min and collect supernatant (nuclear fraction) for Immunoblotting Analysis.

### Immunoblotting analysis

After cell lysis, an equal of proteins were separated on SDS-PAGE gels and transferred to PVDF membranes. Next, 5% milk powder-containing buffer was used to block. Bands were detected using various antibodies as indicated. The membranes were incubated with primary antibodies at 4 °C overnight and secondary antibodies for 1 h at room temperature before exposure to an AI600 system in the dark for band detection. The catalog numbers of the primary antibodies are listed in Supplementary Table S1.

### Coimmunoprecipitation (IP) and IP-MS analysis

To examine the interaction between KDM6A and CDK1, Flag-KDM6A and HA-CKD1 were transfected into HEK293T cells and cultured for 48 h. Cells were lysed with IP lysate and incubated with anti-FLAG and anti-HA antibodies or IgG at 4 °C overnight. The protein A/G magnetic beads and immune complex solution were mixed and incubated at room temperature for 1 h, and then washed to remove the unbound immune complex. The bound immune complex was separated from the microbeads with an ice buffer for Western blotting analysis. The antibodies used in this study are listed in the Supplementary Table S1.

For IP-MS analysis, cells expressing KDM6A phospho-mutants were lysed, and KDM6A-associated complexes were immunoprecipitated using anti-KDM6A antibody with IgG controls. After on-bead tryptic digestion, peptides were analyzed by nanoLC-MS/MS. Data were processed in MaxQuant (MaxLFQ normalization) and filtered using the CRAPome database (>20% frequency threshold). Significant interactors were defined as fold change > 2 and *p* < 0.05, with technical triplicates for reproducibility.

### Seahorse XF cell glycolysis stress test assay

Hippocampal cell glycolytic stress tests were used to detect cell glycolytic capacity. In short, cells were inoculated in XFe96 cell culture microplates and then allowed to grow overnight. In the test, different compounds were added to different ports respectively, (Port A: glucose with a final concentration of 10 mM; Port B: oligomycin, the final concentration is 1 μM; Port C: 2-DG with a final concentration of 50 mM). The experiment was then run on Agilent Seahorse XFe96, using Wave Desktop and Report Generator software to view and analyze the results.

### Cell proliferation and colony formation assays

For the cell proliferation assay, the MTT was added to the well and incubated at 37 °C for 4 h. After the well were washed with PBS once, DMSO was added, and the plates were shaken for 15 min. The OD values were determined at 450 nm. For colony formation assay, 500 cells were seeded in 6-well plates at 37 °C for 10 d. The cell colonies were fixed with methanol and stained with crystal violet.

### Realtime-PCR assays

Quantitative PCR was performed using an ABI Prism 7300 system (Applied Biosystems, Foster City, CA, USA) and SYBR Green (Takara, Dalian, China). For PCR, up to 1 μl of cDNA was used as a template. The thermal cycling conditions were 95 °C for 10 s followed by 40 cycles of 95 °C for 5 s and 60 °C for 30 s. A primer efficiency of >90% was confirmed with a standard curve spanning four orders of magnitude. Following the reactions, the raw data were exported using 7300 System Software 4 v1.3.0 (Applied Biosystems) and analyzed. The primers used are listed in Supplementary Table S1.

### Chromatin Immunoprecipitation (ChIP) assay and ChIP-Seq

HN6 overexpressed different mutants of kdm6a for 48 h. The EZ ChIP kit (Cell Signaling Technology, 9003) were used for ChIP detection. Briefly, HN6 (1 × 107 cells) were fixed with 1% formaldehyde and then neutralized with 0.125 M glycine. Cells were collected and cleaved in a cell lysis buffer containing a mixture of SDS and protease inhibitors. Soluble chromatin with an average length of 500 bp were obtained by ultrasonic treatment of the lysate. After 1:10 dilution in the dilution buffer, the chromatin solutions were pre-cleared and incubated with anti-H3K27Me3 or anti-H3K4Me1 antibodies. Next, the mixtures were incubated overnight on a rotating platform at 4 °C. The immunocomplexes were captured by A protein A/G-Sepharose beads. After a large amount of washing, the bound DNA fragments were eluted, and the obtained DNA were analyzed by real-time PCR using ChIP primers. The primers used are listed in Supplementary Table S1.

ChIP-Seq were performed as previously reported method [54]. The libraries with different indexes were multiplexed and loaded on an Illumina HiSeq instrument according to manufacturer’s instructions (Illumina, San Diego, USA). Sequencing was carried out using a 2 × 150 paired-end (PE) configuration; image analysis and base calling were conducted by the HiSeq Control Software (HCS) + OLB + GAPipeline-1.6 (Illumina) on the HiSeq instrument. The sequences were processed and analyzed by GENEWIZ.

### Immunohistochemistry (IHC)

Tissue sections were sequentially rehydrated with xylene solution and anhydrous ethanol solution of different concentrations, then boiled in sodium citrate antigen recovery solution at 95–100 °C for 10 min for antigen recovery, and endogenous peroxidase was inactivated with 3% hydrogen peroxide. The slices were incubated with anti p-KDM6A overnight at 4 °C, and then incubated with the secondary antibody. Follow the manufacturer’s instructions for using the DAB dyeing kit. After reverse staining with heme, sections were dehydrated with anhydrous ethanol solution and xylene solution of different concentrations in sequence, and then sealed with gum. Microscope analysis was performed using Zeiss AXIO BX61 microscope.

### H&E staining and IF staining

The tissue is stored in 10% buffered formalin phosphate. Paraffin-embedded sections were stained with haematoxylin and eosin according to standard procedure.

For IF staining, sections were incubated with primary antibody and Anti-IgG (Fluorochrome Conjugate) secondary antibody. Sections were mounted with Anti-Fade Mounting Medium and scanned with Digital slice scanner (Pannoramic MIDI, 3DHISTECH).

### Statistical analysis

The investigators were always blinded to the group allocation during the experiments of the study. Statistical analysis was carried out using or GraphPad Prism 6. Data were presented as the mean ± SD. The correlation was determined by Pearson correlation analysis. One-way analysis of variance (ANOVA) was performed to assess the significance of the differences. Student’s *t* test was used for pairwise comparisons between groups. A *p*-value (two-sided) <0.05 was considered as statistically significant. Samples sizes were chosen according to the basis of previous publications without prior power analysis.

## Supplementary information

Supplementary Figures

Supplementary Tables

gel

aj-checklist

## Data Availability

The ChIP-seq and RNA-seq data generated in this study are publicly available in the Genome Sequence Archive, HRA007194 (https://ngdc.cncb.ac.cn/gsa-human/s/cPeMAk9l) and HRA007193 (https://ngdc.cncb.ac.cn/gsa-human/s/X8W4nhOT). Nonprofit research will be approved for access for these data. All other raw data are available upon request from the corresponding author.
