# Supplementary material for: KDM6A phosphorylation suppresses PER2 to confer a glycolytic vulnerability in HNSCC
Source: Cell Death Dis. 2025 Nov 3;16(1):777. doi: 10.1038/s41419-025-08130-w (PMC12583727; doi:10.1038/s41419-025-08130-w)
Supplement: Supplementary file 1 — Supplementary Figures [file 41419_2025_8130_MOESM1_ESM.docx]

**Supplementary Files**


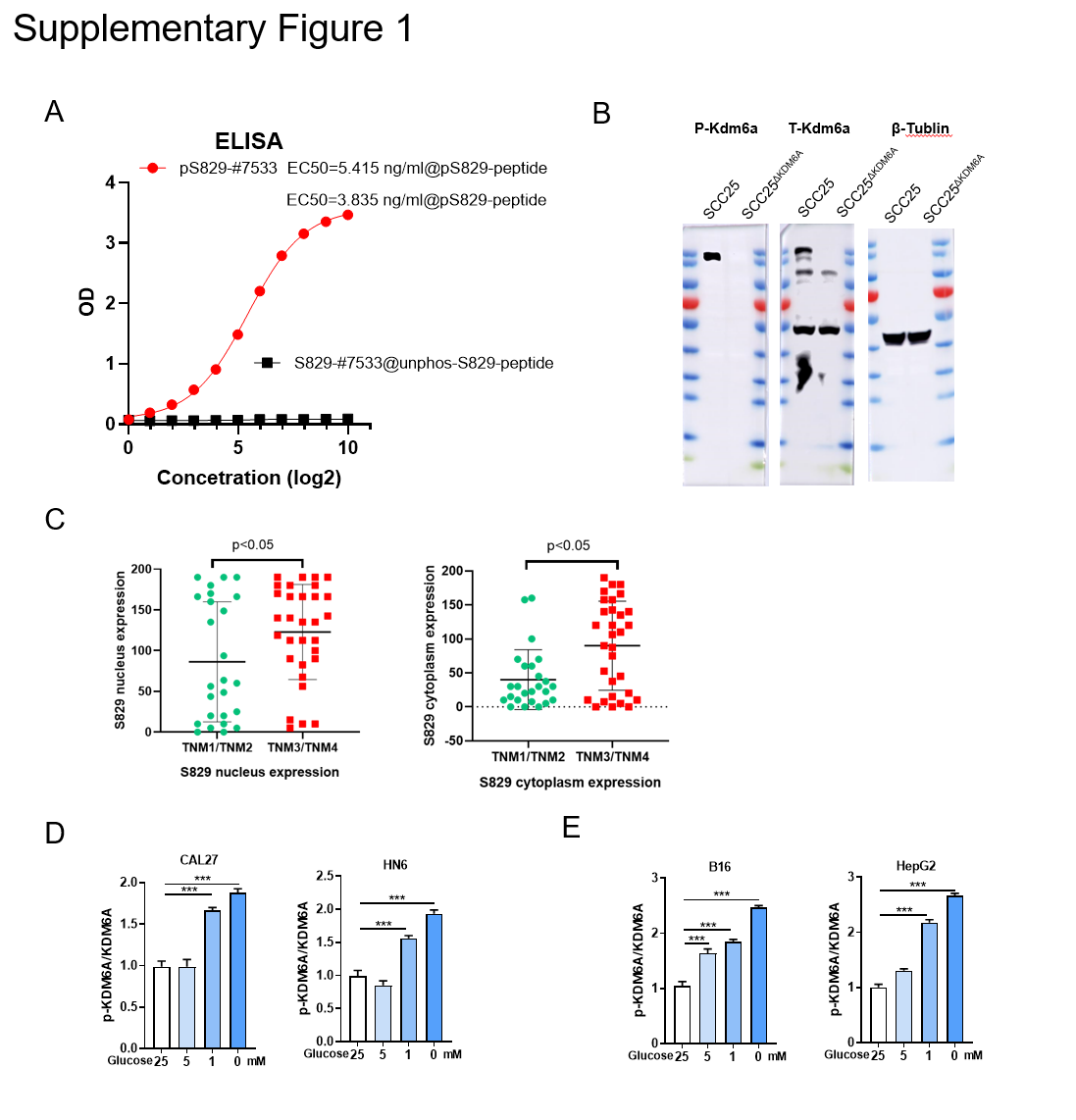


**Figure S1. Generation of rabbit antibody against KDM6A-pSer829 and KDM6A-pSer829 expression in tumor cells**

A. ELISA assays for the generated antibody to bind with phosphor-Ser829-KDM6A-peptide and unphosphor-Ser829-KDM6A-peptide.

B. Immunoblotting against p-Kdm6a, Kdm6a, and β-Tublin in whole cell lysates from SCC25 and SCC25 ^Del KDM6A^ cells.

C. The p^S829^-KDM6A level in nucleus and cytoplasm of tissues at TNM1/TNM2 and TNM3/TNM4 stages of cancer. Data represent mean ± SEM, unpaired two-tailed t test.

D and E. Quantitative Analysis of KDM6A-pSer829 after cells were treated with glucose in the concentration-dependent manner. (n = 3 per group)

Data represent mean ± SEM in C-E. Statistics used unpaired two-tailed t test (C-E). Significance is noted as ∗∗∗p < 0.001.


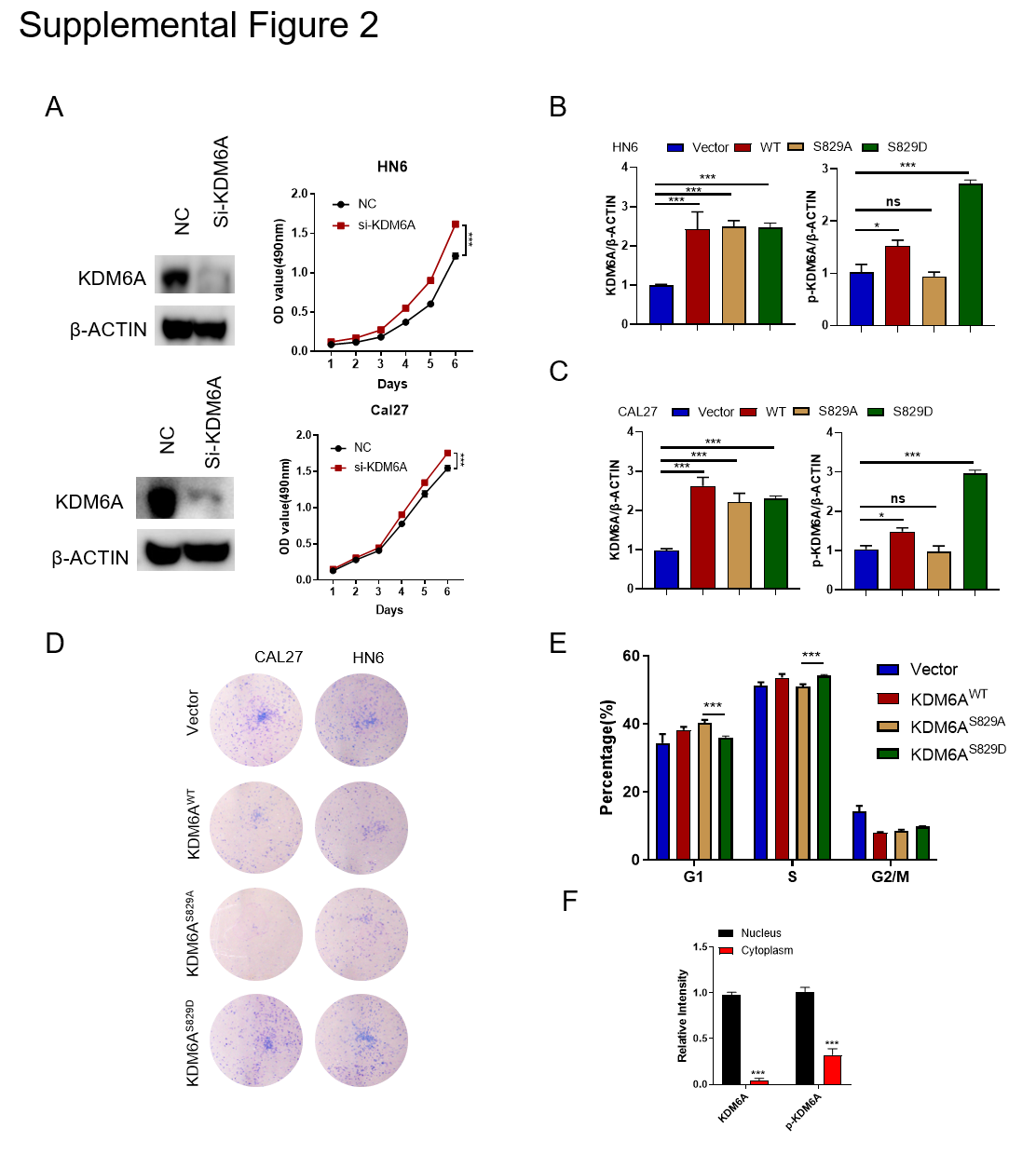


**Figure S2. Knocking down KDM6A or phosphorylation of KDM6A can promote cell proliferation**

A. Left: Immunoblotting against KDM6A and β-ACTIN in whole cell lysates from HN6 or CAL27 cells expressing a control (si-NC) or KDM6A-targeting (si- KDM6A) siRNA. Right: Proliferation of HN6 or CAL27 cells expressing a control (si-NC) or KDM6A-targeting (si- KDM6A) siRNA for 1-6 days (n = 5 per group).

B and C. Quantitative Analysis of p-KDM6A, and KDM6A in whole cell lysates from HN6 or CAL27 cells expressing Vector, KDM6A^WT^, KDM6A^S829A^ or KDM6A^S829D^ for 48 h. (n = 3 per group)

D. Proliferation of HN6 and Cal27 cells expressing Vector, KDM6A-WT, KDM6A-S829A or KDM6A-S829D.

E. The proportion in G1, S, and G2/M phases of HN6 cells expressing Vector, KDM6A^WT^, KDM6A^S829A^ or KDM6A^S829D^.

F. Quantitative Analysis of KDM6A and p-KDM6A in cytoplasm and nucleus fractions of HN6 cells. (n = 3 per group)

Data represent mean ± SEM in A-C and E-F. Statistics used unpaired two-tailed t test (A-C and E-F). Significance is noted as ∗p < 0.05, ∗∗p < 0.01, ∗∗∗p < 0.001.


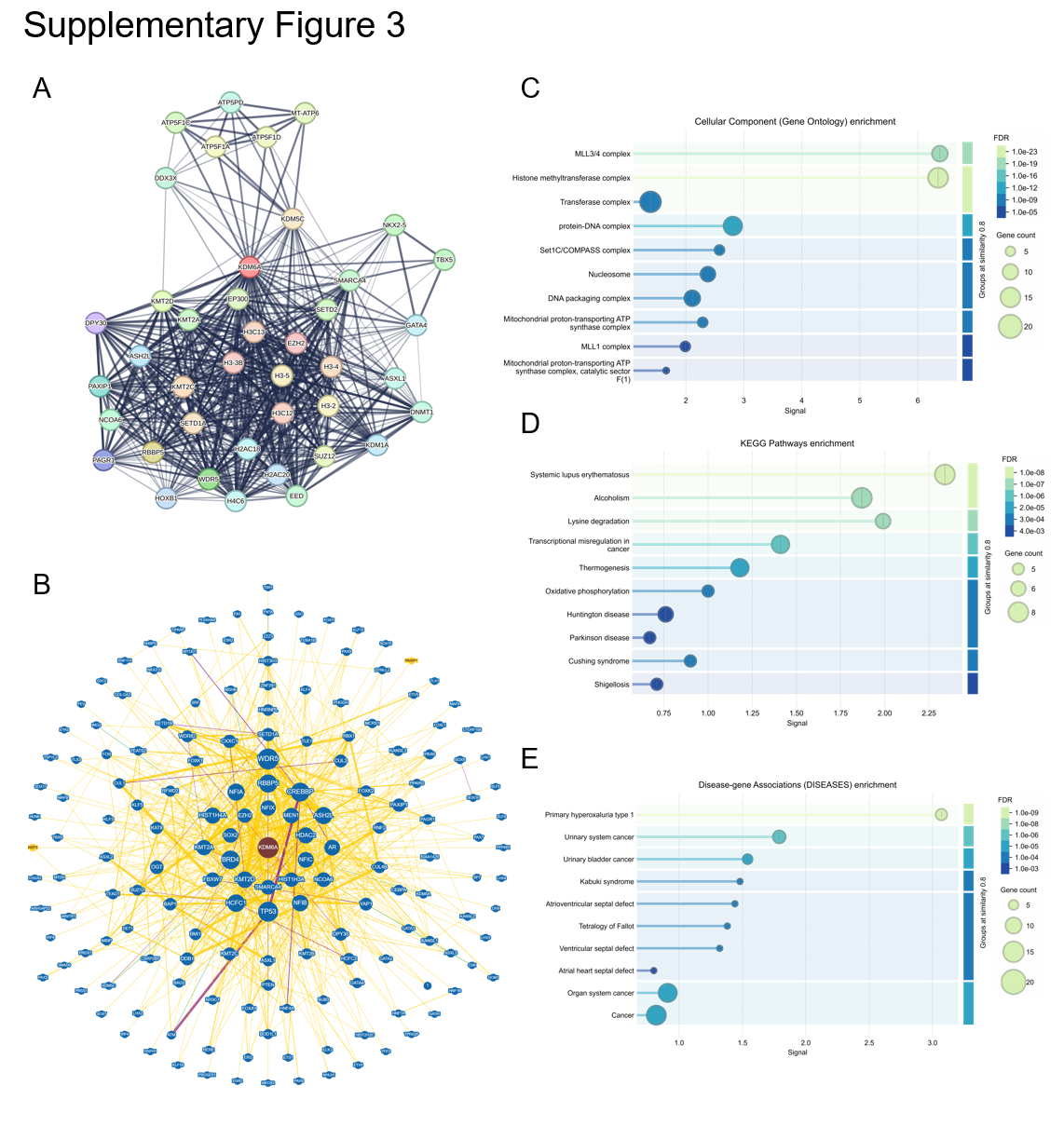


**Figure S3. KDM6A interaction partners and functional analysis**

A. Core KDM6A-associated proteins identified from STRING databases.

B. Core KDM6A-associated proteins identified from BioGRID databases.

C. GO enrichment of KDM6A-associated proteins.

D. KEGG pathway mapping of KDM6A-associated proteins.

E. Disease-gene associations enrichment of KDM6A-associated proteins.


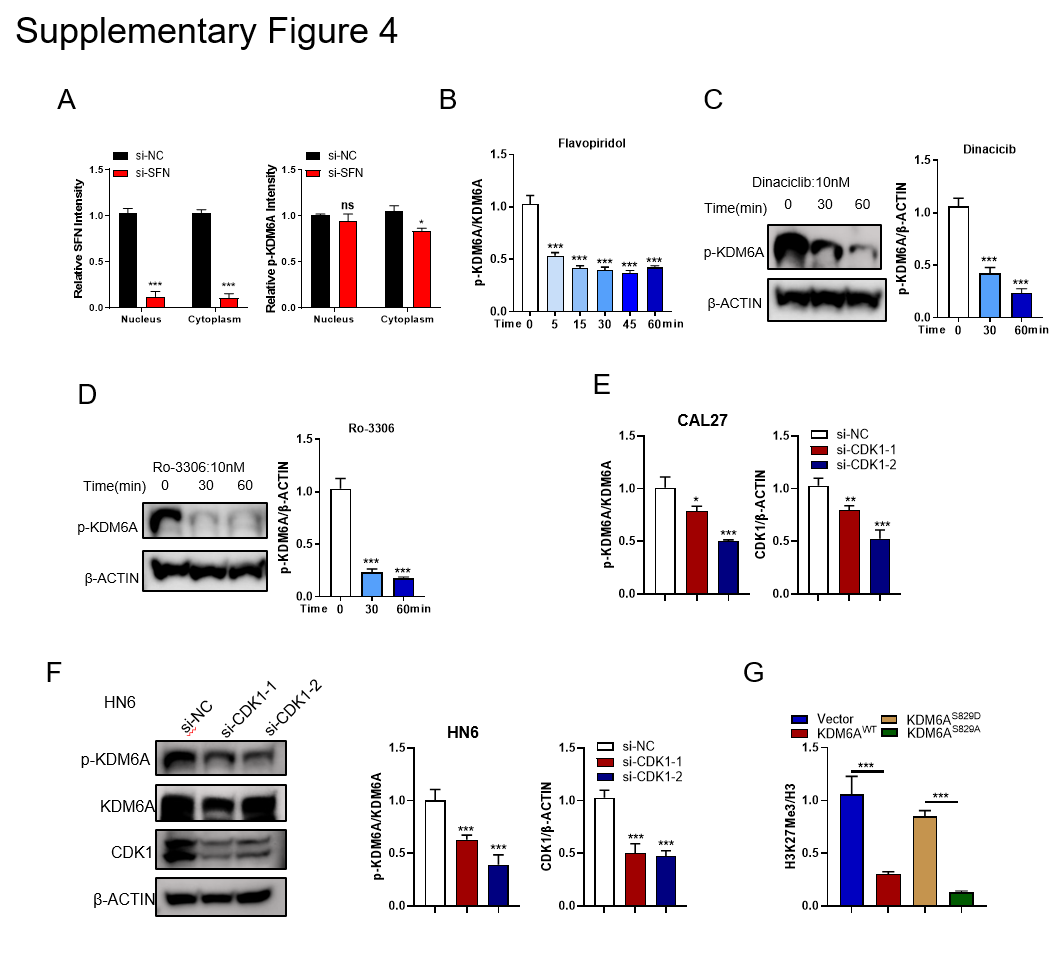


**Figure S4. CDK1-mediated phosphorylation promotes nuclear export of KDM6A**

A. Quantitative Analysis of SFN and p-KDM6A in HN6 cells expressing a control (si-NC) or SFN-targeting (si-SFN) siRNA. (n = 3 per group)

B. Quantitative Analysis of p-KDM6A in whole cell lysates from HN6 cells treated with the CDK1 inhibitor Flavopiridol (10 nM). (n = 3 per group)

C. Left: Immunoblotting against p-KDM6A and TUBLIN in whole cell lysates from HN6 cells treated with Dinaciclib (10 nM). Right: Quantitative Analysis of p-KDM6A level. (n = 3 per group)

D. Left: Immunoblotting against p-KDM6A and TUBLIN in whole cell lysates from HN6 cells treated with Ro-3306 (10 nM). Right: Quantitative Analysis of p-KDM6A level. (n = 3 per group)

E. Quantitative Analysis of p-KDM6A and CDK1 in CAL27 cells expressing a control (si-NC) or CDK1-targeting (CDK1) siRNA. (n = 3 per group)

F. Left: Immunoblotting against p-KDM6A, KDM6A, CDK1, and β-ACTIN in whole cell lysates from HN6 cells expressing a control (si-NC) or CDK1-targeting (si-CDK1) siRNA. Right: Quantitative Analysis of p-KDM6A and CDK1 levels. (n = 3 per group)

G. Quantitative Analysis of H3K27Me3, and H3 in whole cell lysates expressing Vector, KDM6A^WT^, KDM6A^S829A^ or KDM6A^S829D^ for 48 h. (n = 3 per group)

Data represent mean ± SEM in A-G. Statistics used unpaired two-tailed t test (A-G). Significance is noted as ∗p < 0.05, ∗∗p < 0.01, ∗∗∗p < 0.001.


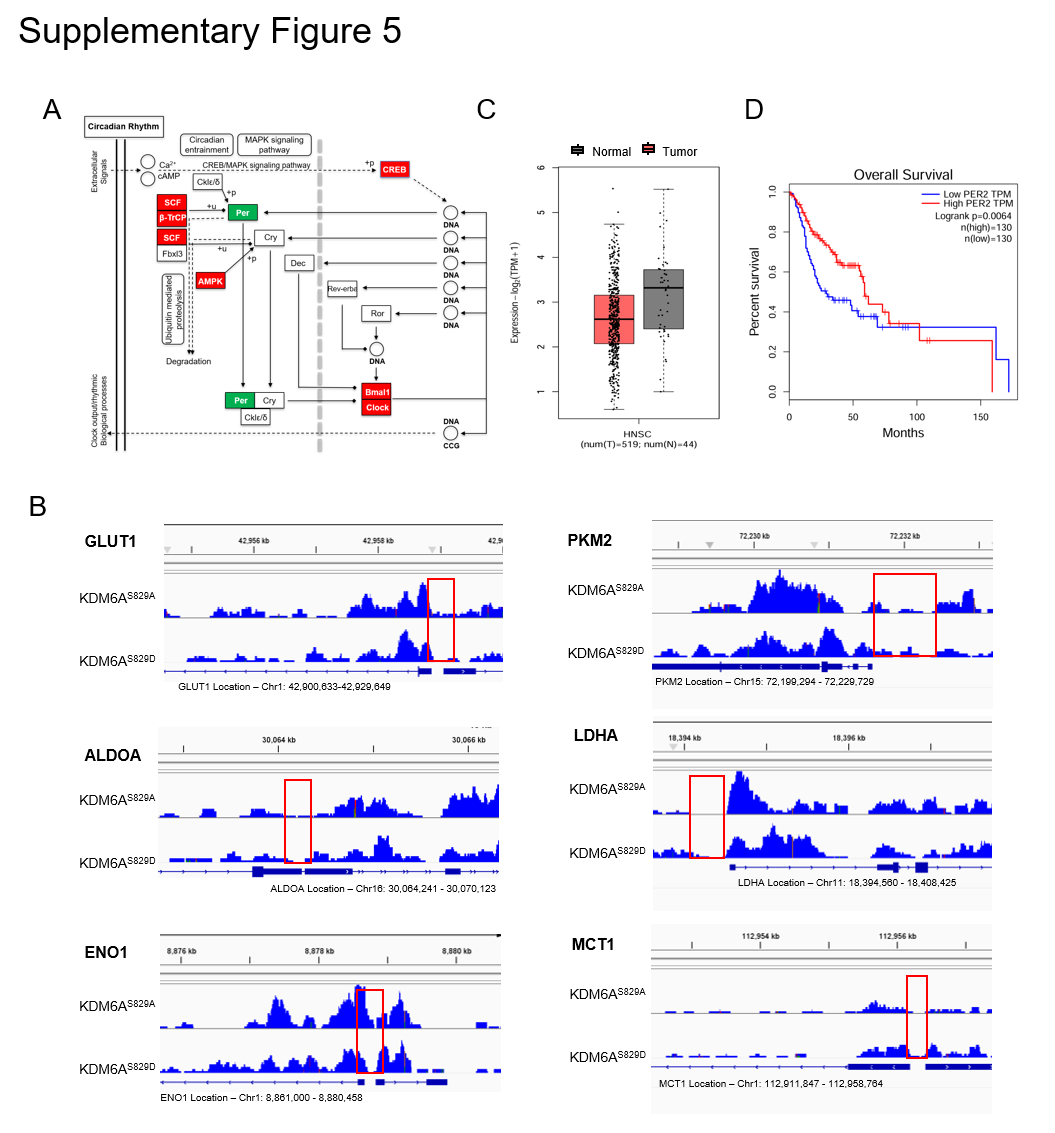


**Figure S5. KDM6A-pSer829 results in differentially gene expression in circadian rhythm**

A. Gene expression in circadian rhythms. Green represents down expression and red represents up expression.

B. Genome browser view of normalized ChIP-Seq signals for H3K27Me3 at *GLUT1*, *ALDOA*, *ENO1*, *PKM2*, *LDHA*, and *MCT1* locus in HN6 cells expressing KDM6A^S829A^ or KDM6A^S829D^.

C. PER2 expression levels in tumor and matched normal tissues from HNSCC patients in the TCGA cohort.

D. Estimated survival showing overall survival between PER2-high and PER2-low expression group from HNSCC patients in the TCGA cohort. The lower quartile was used as a cutoff to divide the samples into low (the lowest 25%) and high (the remaining 75%) PER2 mRNA groups.


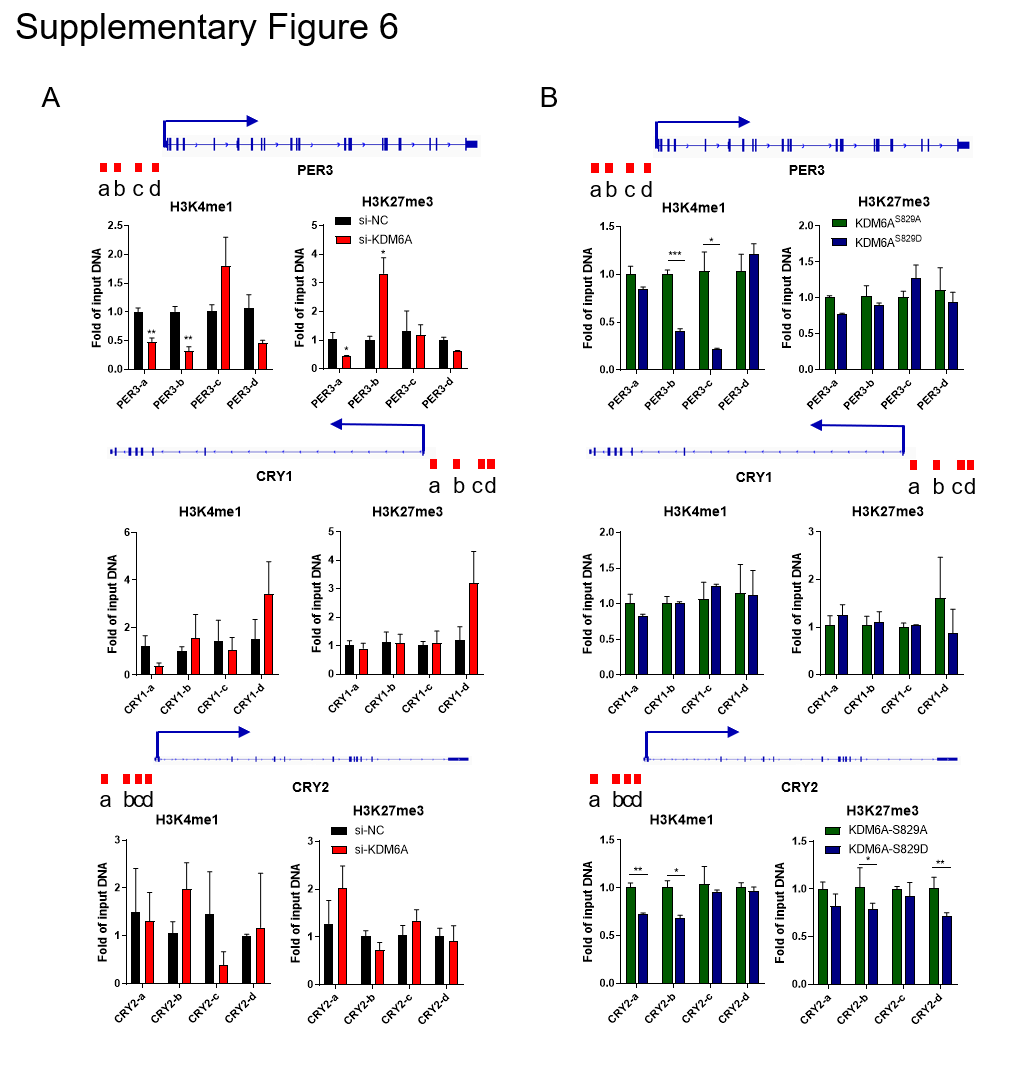


**Figure S6. KDM6A-pSer829 results in differentially gene expression in circadian rhythm**

A. ChIP-qPCR analysis of H3K27Me3 and H3K4Me1 modifications at the PER3, CRY1 and CRY2 loci upon HN6 cells expressing a control (si-NC) or CDK1-targeting (si-CDK1) siRNA (n = 3 per group).

B. ChIP-qPCR analysis of H3K27Me3 and H3K4Me1 modifications at the PER3, CRY1 and CRY2 loci upon ectopic expression of KDM6A^S829A^ or KDM6A^S829D^ in HN6 cells (n = 3 per group). Data represent mean ± SEM, ∗p < 0.05, ∗∗p < 0.01, ∗∗∗p < 0.001, unpaired two-tailed t test.


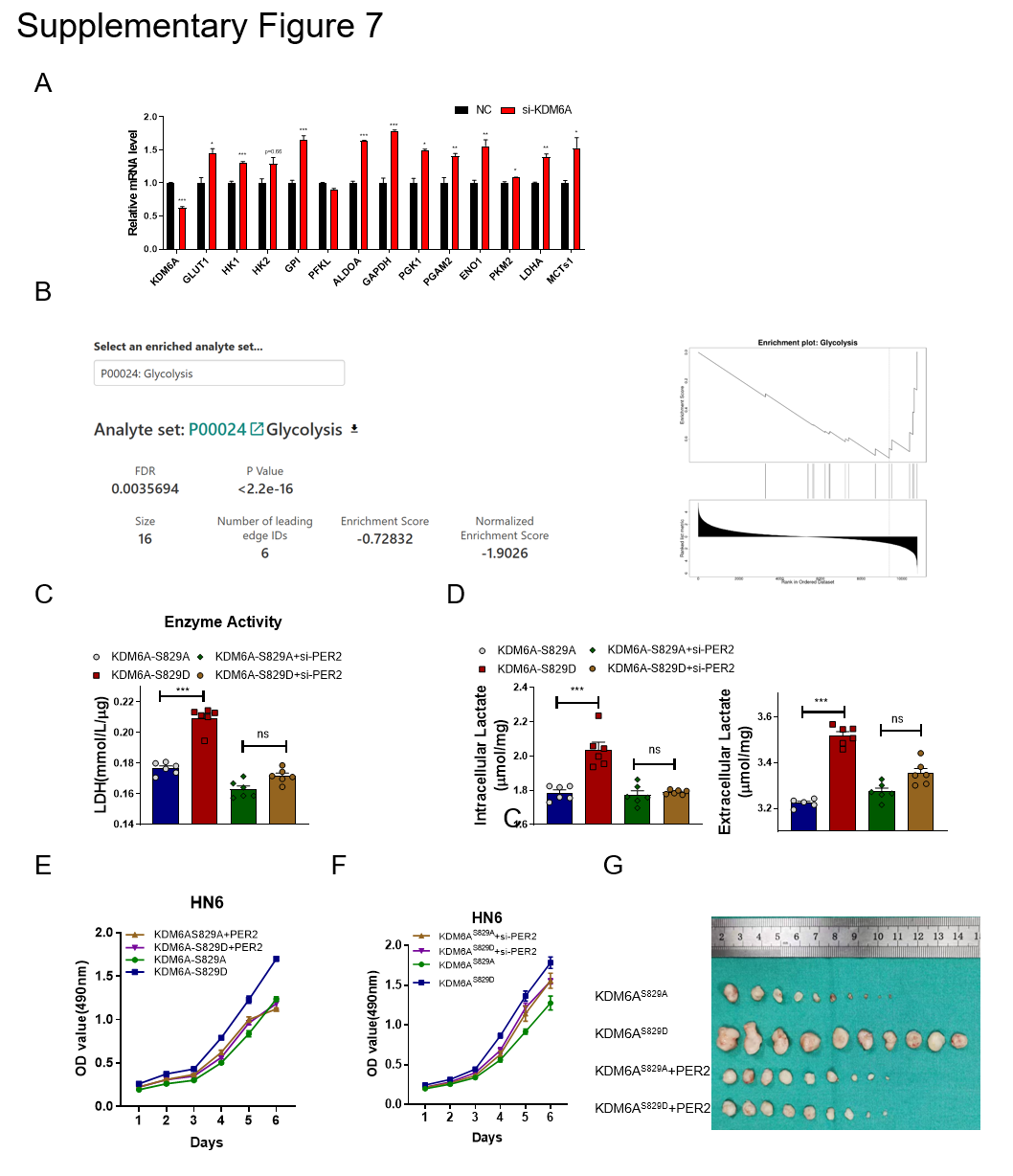


**Figure S7. KDM6A-pSer829 suppresses *PER2* mRNA expression**

A. Relative expression of *KDM6A*, *GLUT1*, *HK1*, *HK2,* *GPI*, *PFKL*, *ALDOA*, *GAPDH*, *PGK1*, *PGAM2*, *ENO1*, *PKM2*, *LDHA*, and *MCTs1* in HN6 cells expressing a control (si-NC) or KDM6A-targeting (si- KDM6A) siRNA for 24 h (n = 3 per group).

B. GSEA analysis to indicate KDM6A protein levels negatively correlated to Glycolysis gene expression in HNSCC tissues.

C. Enzyme activity of LDH in HN6 cells expressing KDM6A^S829A^, KDM6A^S829D^, KDM6A^S829A^+si-PER2 or KDM6A^S829D^ +si-PER2 for 48 h (n = 3 per group).

D. Extracellular lactate and intracellular lactate in the HN6 cells expressing KDM6A^S829A^, KDM6A^S829D^, KDM6A^S829A^+si-PER2 or KDM6A^S829D^ +si-PER2 for 48 h (n = 3 per group).

E. Proliferation of HN6 cells expressing KDM6A^S829A^, KDM6A^S829D^, KDM6A^S829A^+PER2 or KDM6A^S829D^ + PER2 for 1-6 days (n = 5 per group).

F. Proliferation of HN6 cells expressing KDM6A^S829A^, KDM6A^S829D^, KDM6A^S829A^+si-PER2 or KDM6A^S829D^ +si-PER2 for 1-6 days (n = 5 per group).

G. Tumor image of HN6 xenografts (n = 8 mice per group).

Data represent mean ± SEM in A and C-F. Statistics used unpaired two-tailed t test (A and C-F). Significance is noted as ∗p < 0.05, ∗∗p < 0.01, ∗∗∗p < 0.001.
