# Supplementary material for: KDM6A phosphorylation suppresses PER2 to confer a glycolytic vulnerability in HNSCC
Source: Cell Death Dis. 2025 Nov 3;16(1):777. doi: 10.1038/s41419-025-08130-w (PMC12583727; doi:10.1038/s41419-025-08130-w)
Supplement: Supplementary file 2 — Supplementary Tables [file 41419_2025_8130_MOESM2_ESM.docx]

**Table S1. Antibody list**

| **REAGENT or RESOURCE** | **SOURCE** | **IDENTIFIER** |
| --- | --- | --- |
| **IHC, ICC, Immunoblotting** |  | https://www.antibodyregistry.org/ |
| KDM6A | Cell Signaling Technology | 33510 |
| SFN | Cell Signaling Technology | 9635 |
| CDK1 | ABclonal | A11420 |
| β-actin | ABclonal | AC026 |
| FLAG | Cell Signaling Technology | 14793 |
| HA | Cell Signaling Technology | 3724 |
| FLAG-M2-Beads | Cell Signaling Technology | 82103 |
| IgG | Cell Signaling Technology | 2729 |
| Tubulin | ABclonal | AC015 |
| GAPDH | ABclonal | A19056 |
| H3 | Cell Signaling Technology | 4620 |
| Ki67 | ABclonal | A20018 |
| p-KDM6A-S829 | This paper | N/A |
| **ChIP assay** |  |  |
| ChIP Kit | Cell Signaling Technology | 9005 |
| H3K27Me3 | Cell Signaling Technology | 9733 |
| H3K4Me1 | Cell Signaling Technology | 5326 |
| Assay Kits |  |  |
| [Glucose Assay Kit](http://www.njjcbio.com/products.asp?id=812) | Nanjing Jiancheng Bioengineering Institute | A154-1-1 |
| [Lactic Acid assay kit](http://www.njjcbio.com/products.asp?id=319) | Nanjing Jiancheng Bioengineering Institute | [A019-1-1](http://www.njjcbio.com/products.asp?id=319) |
| [Hexokinase assay kit](http://www.njjcbio.com/products.asp?id=3059) | Nanjing Jiancheng Bioengineering Institute | [A077-3-1](http://www.njjcbio.com/products.asp?id=3059) |
| [Phosphofructokinase test kit](http://www.njjcbio.com/products.asp?id=2610) | Nanjing Jiancheng Bioengineering Institute | [A129-1-1](http://www.njjcbio.com/products.asp?id=2610) |
| [Pyruvate kinase assay kit](http://www.njjcbio.com/products.asp?id=405) | Nanjing Jiancheng Bioengineering Institute | [A076-1-1](http://www.njjcbio.com/products.asp?id=405) |
| [Lactate dehydrogenase assay kit](http://www.njjcbio.com/products.asp?id=322) | Nanjing Jiancheng Bioengineering Institute | [A020-1-2](http://www.njjcbio.com/products.asp?id=322) |
| Chemicals, peptides,  and recombinant proteins |  |  |
| FBS | Gibco | A5669701 |
| DMEM | Gibco | 11965092 |
| DMEM, no Glucose, pyruvate | Gibco | 11966025 |
| **Experimental models: Cell lines** |  |  |
| Human cell line: CAL27  Sex: M | ATCC | CRL-2095 |
| Human cell line: HN6  Sex: Unknown | University of Maryland Dental School, USA | N/A |
| Human cell line: SCC25  Sex: M | ATCC | [CRL-1628](https://www.atcc.org/products/CRL-1628) |
| Mouse cell line: B16-F0  Sex: Unknown | ATCC | CRL-6322 |
| Human cell line: Hep G2  Sex: M | ATCC | [HB-8065](https://www.atcc.org/products/CRL-1628) |
| **Experimental models:**  **Organisms/strains** |  |  |
| MOUSE: C57BL/6J | Jackson | Cat#000664; RRID:IMSR_JAX:000664 |
| MOUSE: BALB/C nude | SHANGHAI MODEL ORGANISMS | SM-014 |
| **Oligonucleotides** |  |  |
| b-ACTIN Forward: CATGTACGTTGCTATCCAGGC | Purchased: Sangon Biotech | Design: ncbi.nlm.nih.gov/tools/primer-blast/ |
| b- ACTIN Reverse: CTCCTTAATGTCACGCACGAT | Purchased: Sangon Biotech | Design: ncbi.nlm.nih.gov/tools/primer-blast/ |
| KDM6A Forward: TTCCTCGGAAGGTGCTATTCA | Purchased: Sangon Biotech | Design: ncbi.nlm.nih.gov/tools/primer-blast/ |
| KDM6A Reverse: GAGGCTGGTTGCAGGATTCA | Purchased: Sangon Biotech | Design: ncbi.nlm.nih.gov/tools/primer-blast/ |
| CRY1 Forward: CTCCTCCAATGTGGGCATCAA | Purchased: Sangon Biotech | Design: ncbi.nlm.nih.gov/tools/primer-blast/ |
| CRY1 Reverse: CCACGAATCACAAACAGACGG | Purchased: Sangon Biotech | Design: ncbi.nlm.nih.gov/tools/primer-blast/ |
| CRY2 Forward: TCCCAAGGCTGTTCAAGGAAT | Purchased: Sangon Biotech | Design: ncbi.nlm.nih.gov/tools/primer-blast/ |
| CRY2 Reverse: TGCATCCCGTTCTTTCCCAAA | Purchased: Sangon Biotech | Design: ncbi.nlm.nih.gov/tools/primer-blast/ |
| BMAL1 Forward: AAGGGAAGCTCACAGTCAGAT | Purchased: Sangon Biotech | Design: ncbi.nlm.nih.gov/tools/primer-blast/ |
| BMAL1 Reverse: GGACATTGCGTTGCATGTTGG | Purchased: Sangon Biotech | Design: ncbi.nlm.nih.gov/tools/primer-blast/ |
| CLOCK Forward: TGCGAGGAACAATAGACCCAA | Purchased: Sangon Biotech | Design: ncbi.nlm.nih.gov/tools/primer-blast/ |
| CLOCK Reverse: ATGGCCTATGTGTGCGTTGTA | Purchased: Sangon Biotech | Design: ncbi.nlm.nih.gov/tools/primer-blast/ |
| PER3 Forward: GCAGAGGAAATTGGCGGACA | Purchased: Sangon Biotech | Design: ncbi.nlm.nih.gov/tools/primer-blast/ |
| PER3 Reverse: GGTTTATTGCGTCTCTCCGAG | Purchased: Sangon Biotech | Design: ncbi.nlm.nih.gov/tools/primer-blast/ |
| PER2 Forward: GACATGAGACCAACGAAAACTGC | Purchased: Sangon Biotech | Design: ncbi.nlm.nih.gov/tools/primer-blast/ |
| PER2 Reverse: AGGCTAAAGGTATCTGGACTCTG | Purchased: Sangon Biotech | Design: ncbi.nlm.nih.gov/tools/primer-blast/ |
| PER1 Forward: GCCAACCAGGAATACTACCAGC | Purchased: Sangon Biotech | Design: ncbi.nlm.nih.gov/tools/primer-blast/ |
| PER1 Reverse: GTGTGTACTCAGACGTGATGTG | Purchased: Sangon Biotech | Design: ncbi.nlm.nih.gov/tools/primer-blast/ |
| HK1 Forward: GCTCTCCGATGAAACTCTCATAG | Purchased: Sangon Biotech | Design: ncbi.nlm.nih.gov/tools/primer-blast/ |
| HK1 Reverse: GGACCTTACGAATGTTGGCAA | Purchased: Sangon Biotech | Design: ncbi.nlm.nih.gov/tools/primer-blast/ |
| PFKL Forward: GCTGGGCGGCACTATCATT | Purchased: Sangon Biotech | Design: ncbi.nlm.nih.gov/tools/primer-blast/ |
| PFKL Reverse: TCAGGTGCGAGTAGGTCCG | Purchased: Sangon Biotech | Design: ncbi.nlm.nih.gov/tools/primer-blast/ |
| PKM2 Forward: ATGTCGAAGCCCCATAGTGAA | Purchased: Sangon Biotech | Design: ncbi.nlm.nih.gov/tools/primer-blast/ |
| PKM2 Reverse: TGGGTGGTGAATCAATGTCCA | Purchased: Sangon Biotech | Design: ncbi.nlm.nih.gov/tools/primer-blast/ |
| LDHA Forward: ATGGCAACTCTAAAGGATCAGC | Purchased: Sangon Biotech | Design: ncbi.nlm.nih.gov/tools/primer-blast/ |
| LDHA Reverse: CCAACCCCAACAACTGTAATCT | Purchased: Sangon Biotech | Design: ncbi.nlm.nih.gov/tools/primer-blast/ |
| GAPDH Forward: GGAGCGAGATCCCTCCAAAAT | Purchased: Sangon Biotech | Design: ncbi.nlm.nih.gov/tools/primer-blast/ |
| GAPDH Reverse: GGCTGTTGTCATACTTCTCATGG | Purchased: Sangon Biotech | Design: ncbi.nlm.nih.gov/tools/primer-blast/ |
| GPI Forward: CAAGGACCGCTTCAACCACTT | Purchased: Sangon Biotech | Design: ncbi.nlm.nih.gov/tools/primer-blast/ |
| GPI Reverse: CCAGGATGGGTGTGTTTGACC | Purchased: Sangon Biotech | Design: ncbi.nlm.nih.gov/tools/primer-blast/ |
| ALDOA Forward: ATGCCCTACCAATATCCAGCA | Purchased: Sangon Biotech | Design: ncbi.nlm.nih.gov/tools/primer-blast/ |
| ALDOA Reverse: GCTCCCAGTGGACTCATCTG | Purchased: Sangon Biotech | Design: ncbi.nlm.nih.gov/tools/primer-blast/ |
| GLUT1 Forward: CATCCCATGGTTCATCGTGGCTGAACT | Purchased: Sangon Biotech | Design: ncbi.nlm.nih.gov/tools/primer-blast/ |
| GLUT1 Reverse: GAAGTAGGTGAAGATGAAGAACAGAAC | Purchased: Sangon Biotech | Design: ncbi.nlm.nih.gov/tools/primer-blast/ |
| PGK1 Forward: TGGACGTTAAAGGGAAGCGG | Purchased: Sangon Biotech | Design: ncbi.nlm.nih.gov/tools/primer-blast/ |
| PGK1 Reverse: GCTCATAAGGACTACCGACTTGG | Purchased: Sangon Biotech | Design: ncbi.nlm.nih.gov/tools/primer-blast/ |
| ENO1 Forward: AAAGCTGGTGCCGTTGAGAA | Purchased: Sangon Biotech | Design: ncbi.nlm.nih.gov/tools/primer-blast/ |
| ENO1 Reverse: GGTTGTGGTAAACCTCTGCTC | Purchased: Sangon Biotech | Design: ncbi.nlm.nih.gov/tools/primer-blast/ |
| PGAM2 Forward: AGAAGCACCCCTACTACAACTC | Purchased: Sangon Biotech | Design: ncbi.nlm.nih.gov/tools/primer-blast/ |
| PGAM2 Reverse: TCTGGGGAACAATCTCCTCGT | Purchased: Sangon Biotech | Design: ncbi.nlm.nih.gov/tools/primer-blast/ |
| GLUT4 Forward: TGGGCGGCATGATTTCCTC | Purchased: Sangon Biotech | Design: ncbi.nlm.nih.gov/tools/primer-blast/ |
| GLUT4 Reverse: GCCAGGACATTGTTGACCAG | Purchased: Sangon Biotech | Design: ncbi.nlm.nih.gov/tools/primer-blast/ |
| PDHA1 Forward: TGGTAGCATCCCGTAATTTTGC | Purchased: Sangon Biotech | Design: ncbi.nlm.nih.gov/tools/primer-blast/ |
| PDHA1 Reverse: ATTCGGCGTACAGTCTGCATC | Purchased: Sangon Biotech | Design: ncbi.nlm.nih.gov/tools/primer-blast/ |
| GPT2 Forward: GTGATGGCACTATGCACCTAC | Purchased: Sangon Biotech | Design: ncbi.nlm.nih.gov/tools/primer-blast/ |
| GPT2 Reverse: TTCACGGATGCAGTTGACACC | Purchased: Sangon Biotech | Design: ncbi.nlm.nih.gov/tools/primer-blast/ |
| MCT1 Forward: AGGTCCAGTTGGATACACCCC | Purchased: Sangon Biotech | Design: ncbi.nlm.nih.gov/tools/primer-blast/ |
| MCT1 Reverse: GCATAAGAGAAGCCGATGGAAAT | Purchased: Sangon Biotech | Design: ncbi.nlm.nih.gov/tools/primer-blast/ |
| Si-PER2-1 sense: UCUUAUUUCUUUGAACUUCUG | Purchased: RiboBio | Design: sidirect2.rnai.jp |
| Si-PER2-1 antisense: GAAGUUCAAAGAAAUAAGAAA | Purchased: RiboBio | Design: sidirect2.rnai.jp |
| Si-PER2-2 sense: ACCAUUUUUACAAAUUUCGGC | Purchased: RiboBio | Design: sidirect2.rnai.jp |
| Si-PER2-2 antisense: CGAAAUUUGUAAAAAUGGUAA | Purchased: RiboBio | Design: sidirect2.rnai.jp |
| Si-PER1-1 sense: ACUCAUUGCCACUUGAACCAU | Purchased: RiboBio | Design: sidirect2.rnai.jp |
| Si-PER1-1 antisense: GGUUCAAGUGGCAAUGAGUCC | Purchased: RiboBio | Design: sidirect2.rnai.jp |
| Si-PER1-2 sense: UAUCAAAAACACAAAUGCCAU | Purchased: RiboBio | Design: sidirect2.rnai.jp |
| Si-PER1-2 antisense: GGCAUUUGUGUUUUUGAUAUU | Purchased: RiboBio | Design: sidirect2.rnai.jp |
| Si-CDK1-1 sense: UAAUCUUCCAUAGUUAGUCAA | Purchased: RiboBio | Design: sidirect2.rnai.jp |
| Si- CDK1-1 antisense: GACUAACUAUGGAAGAUUAUA | Purchased: RiboBio | Design: sidirect2.rnai.jp |
| Si- CDK1-2 sense: UAUUUGGAUGACGAAGUUCCU | Purchased: RiboBio | Design: sidirect2.rnai.jp |
| Si- CDK1-2 antisense: GAACUUCGUCAUCCAAAUAUA | Purchased: RiboBio | Design: sidirect2.rnai.jp |
| d-PER3 Forward: CCTATCATTTCTGCGCTGGC | Purchased: Sangon Biotech | Design: ncbi.nlm.nih.gov/tools/primer-blast/ |
| d-PER3 Reverse: GACTCCGCAGGCCTCCATT | Purchased: Sangon Biotech | Design: ncbi.nlm.nih.gov/tools/primer-blast/ |
| c-PER3 Forward: AAAACCGTGGGAACCCAGAAA | Purchased: Sangon Biotech | Design: ncbi.nlm.nih.gov/tools/primer-blast/ |
| c-PER3 Reverse: GAGTCCTGGAGTTTGTAGGTGG | Purchased: Sangon Biotech | Design: ncbi.nlm.nih.gov/tools/primer-blast/ |
| b-PER3 Forward: CTCACCGAAAGAGTTAGCGGA | Purchased: Sangon Biotech | Design: ncbi.nlm.nih.gov/tools/primer-blast/ |
| b-PER3 Reverse: TCGTGCTTTGGCCGTCTTT | Purchased: Sangon Biotech | Design: ncbi.nlm.nih.gov/tools/primer-blast/ |
| a-PER3 Forward: AAGGGCTCTACAACTTCTTTTCTGA | Purchased: Sangon Biotech | Design: ncbi.nlm.nih.gov/tools/primer-blast/ |
| a-PER3 Reverse: ACACTTCCCTGCACTTCCTAA | Purchased: Sangon Biotech | Design: ncbi.nlm.nih.gov/tools/primer-blast/ |
| d-PER1 Forward: CTCACGTGTATCTCTCCCGC | Purchased: Sangon Biotech | Design: ncbi.nlm.nih.gov/tools/primer-blast/ |
| d-PER1 Reverse: CCCCCACGCTTATTGGCTTA | Purchased: Sangon Biotech | Design: ncbi.nlm.nih.gov/tools/primer-blast/ |
| c-PER1 Forward: CTGGGTAAACAAGTTGCCGC | Purchased: Sangon Biotech | Design: ncbi.nlm.nih.gov/tools/primer-blast/ |
| c-PER1 Reverse: GGGAAATAACCCCCGACCTG | Purchased: Sangon Biotech | Design: ncbi.nlm.nih.gov/tools/primer-blast/ |
| b-PER1 Forward: CATCGTCACCAAGACACCGT | Purchased: Sangon Biotech | Design: ncbi.nlm.nih.gov/tools/primer-blast/ |
| b-PER1 Reverse: ACGACACTCTTAACCACGGC | Purchased: Sangon Biotech | Design: ncbi.nlm.nih.gov/tools/primer-blast/ |
| a-PER1 Forward: AGGCAGTGCTCAGGATTTCG | Purchased: Sangon Biotech | Design: ncbi.nlm.nih.gov/tools/primer-blast/ |
| a-PER1 Reverse: CGCCTGTAACACGCTAGTCA | Purchased: Sangon Biotech | Design: ncbi.nlm.nih.gov/tools/primer-blast/ |
| a-CRY1 Forward: AGCATTTGCCTATGTCAGGCT | Purchased: Sangon Biotech | Design: ncbi.nlm.nih.gov/tools/primer-blast/ |
| a-CRY1 Reverse: ACCTCCCTGGGTTCTCCAAG | Purchased: Sangon Biotech | Design: ncbi.nlm.nih.gov/tools/primer-blast/ |
| b-CRY1 Forward: GCAGTTCACTGTCGCTGGAA | Purchased: Sangon Biotech | Design: ncbi.nlm.nih.gov/tools/primer-blast/ |
| b-CRY1 Reverse: AGGGTAATTGGCAGGACGGA | Purchased: Sangon Biotech | Design: ncbi.nlm.nih.gov/tools/primer-blast/ |
| c-CRY1 Forward: GTTCTCTGGCCAAAGCAGGA | Purchased: Sangon Biotech | Design: ncbi.nlm.nih.gov/tools/primer-blast/ |
| c-CRY1 Reverse: GGCCTGGAAATTGTCCGGTT | Purchased: Sangon Biotech | Design: ncbi.nlm.nih.gov/tools/primer-blast/ |
| d-CRY1 Forward: AAATCCCACGGGAGGAATCG | Purchased: Sangon Biotech | Design: ncbi.nlm.nih.gov/tools/primer-blast/ |
| d-CRY1 Reverse: CTGAGACCCGGATGAGCAC | Purchased: Sangon Biotech | Design: ncbi.nlm.nih.gov/tools/primer-blast/ |
| d-CRY2 Forward: CAGCTTTTTCACTGGGGTTCG | Purchased: Sangon Biotech | Design: ncbi.nlm.nih.gov/tools/primer-blast/ |
| d-CRY2 Reverse: GGTAGGGGTTGCTGTGACTAC | Purchased: Sangon Biotech | Design: ncbi.nlm.nih.gov/tools/primer-blast/ |
| c-CRY2 Forward: TCCTTCCCCGTCCTTAGACT | Purchased: Sangon Biotech | Design: ncbi.nlm.nih.gov/tools/primer-blast/ |
| c-CRY2 Reverse: TGAAGAAAACGGAGGGTGCC | Purchased: Sangon Biotech | Design: ncbi.nlm.nih.gov/tools/primer-blast/ |
| b-CRY2 Forward: GTCCAGGAAGGGTTCCAAGC | Purchased: Sangon Biotech | Design: ncbi.nlm.nih.gov/tools/primer-blast/ |
| b-CRY2 Reverse: GCACTGTACTGGATTCCGGG | Purchased: Sangon Biotech | Design: ncbi.nlm.nih.gov/tools/primer-blast/ |
| a-CRY2 Forward: GGAACAGCTCGTGAAAAGCC | Purchased: Sangon Biotech | Design: ncbi.nlm.nih.gov/tools/primer-blast/ |
| a-CRY2 Reverse: TGGACCTGGGACAGAGTGAT | Purchased: Sangon Biotech | Design: ncbi.nlm.nih.gov/tools/primer-blast/ |
| a-CLOCK Forward: GGGAATGAATGCTGAACAGGC | Purchased: Sangon Biotech | Design: ncbi.nlm.nih.gov/tools/primer-blast/ |
| a-CLOCK Reverse: TTGCTGTATGCTCTGGGCAA | Purchased: Sangon Biotech | Design: ncbi.nlm.nih.gov/tools/primer-blast/ |
| b-CLOCK Forward: ACACGTTGTTCCAACGAGGA | Purchased: Sangon Biotech | Design: ncbi.nlm.nih.gov/tools/primer-blast/ |
| b-CLOCK Reverse: CAGAGGAGGGAAGCGAAAGG | Purchased: Sangon Biotech | Design: ncbi.nlm.nih.gov/tools/primer-blast/ |
| c-CLOCK Forward: ACTCTTCTCTCCCAGCACCA | Purchased: Sangon Biotech | Design: ncbi.nlm.nih.gov/tools/primer-blast/ |
| c-CLOCK Reverse: AAAGCAAAACACTGCCGACG | Purchased: Sangon Biotech | Design: ncbi.nlm.nih.gov/tools/primer-blast/ |
| d-CLOCK Forward: GGAGAGCGGGTTCCTTTTCT | Purchased: Sangon Biotech | Design: ncbi.nlm.nih.gov/tools/primer-blast/ |
| d-CLOCK Reverse: GCTAGCCTTAGGTCCTGGAG | Purchased: Sangon Biotech | Design: ncbi.nlm.nih.gov/tools/primer-blast/ |
| a-BMAL1 Forward: GAACAGGGACACCACAGAGAG | Purchased: Sangon Biotech | Design: ncbi.nlm.nih.gov/tools/primer-blast/ |
| a-BMAL1 Reverse: AATTGCCCCAACTTTTCCCG | Purchased: Sangon Biotech | Design: ncbi.nlm.nih.gov/tools/primer-blast/ |
| b-BMAL1 Forward: CAGTGTGGCAATGCTAGTCG | Purchased: Sangon Biotech | Design: ncbi.nlm.nih.gov/tools/primer-blast/ |
| b-BMAL1 Reverse: TGCAAGAGGGTAATCGCAGC | Purchased: Sangon Biotech | Design: ncbi.nlm.nih.gov/tools/primer-blast/ |
| c-BMAL1 Forward: CCTTTCCACGTGCGGAGTAA | Purchased: Sangon Biotech | Design: ncbi.nlm.nih.gov/tools/primer-blast/ |
| c-BMAL1 Reverse: GACGAGGGCGTTACGATCC | Purchased: Sangon Biotech | Design: ncbi.nlm.nih.gov/tools/primer-blast/ |
| d-BMAL1 Forward: GATCGTAACGCCCTCGTCTC | Purchased: Sangon Biotech | Design: ncbi.nlm.nih.gov/tools/primer-blast/ |
| d-BMAL1 Reverse: GGAGTTTTGATTCCGCCCCC | Purchased: Sangon Biotech | Design: ncbi.nlm.nih.gov/tools/primer-blast/ |
| a-PER2 Forward: TTCCGTGGGCGATTCTCTTT | Purchased: Sangon Biotech | Design: ncbi.nlm.nih.gov/tools/primer-blast/ |
| a-PER2 Reverse: AAAGCTCCTAGATCAGCGCC | Purchased: Sangon Biotech | Design: ncbi.nlm.nih.gov/tools/primer-blast/ |
| b-PER2 Forward: TGAGGGCGTAGTGAATGGAAG | Purchased: Sangon Biotech | Design: ncbi.nlm.nih.gov/tools/primer-blast/ |
| b-PER2 Reverse: TCACCTCGTCGGTTCCTCAA | Purchased: Sangon Biotech | Design: ncbi.nlm.nih.gov/tools/primer-blast/ |
| c-PER2 Forward: TCCCCATGAGAGCCTACACA | Purchased: Sangon Biotech | Design: ncbi.nlm.nih.gov/tools/primer-blast/ |
| c-PER2 Reverse: CCCTGCATCACCTTGTTTCG | Purchased: Sangon Biotech | Design: ncbi.nlm.nih.gov/tools/primer-blast/ |
| d-PER2 Forward: TTGCTTGGCATTTGTGTGTGT | Purchased: Sangon Biotech | Design: ncbi.nlm.nih.gov/tools/primer-blast/ |
| d-PER2 Reverse: ATACCAAGTTCATCGGGGCAG | Purchased: Sangon Biotech | Design: ncbi.nlm.nih.gov/tools/primer-blast/ |
|  |  |  |
| **Software and algorithms** |  |  |
| Prism | Graphpad | www.graphpad.com |
| Excel | Microsoft | www.microsoft.com |
| ImageJ | NIH | imagej.nih.gov/ij/ |
|  |  |  |
