# Supplementary material for: KDM6A phosphorylation suppresses PER2 to confer a glycolytic vulnerability in HNSCC
Source: Cell Death Dis. 2025 Nov 3;16(1):777. doi: 10.1038/s41419-025-08130-w (PMC12583727; doi:10.1038/s41419-025-08130-w)
Supplement: Supplementary file 3 — gel [file 41419_2025_8130_MOESM3_ESM.docx]

**Fig. 2C**


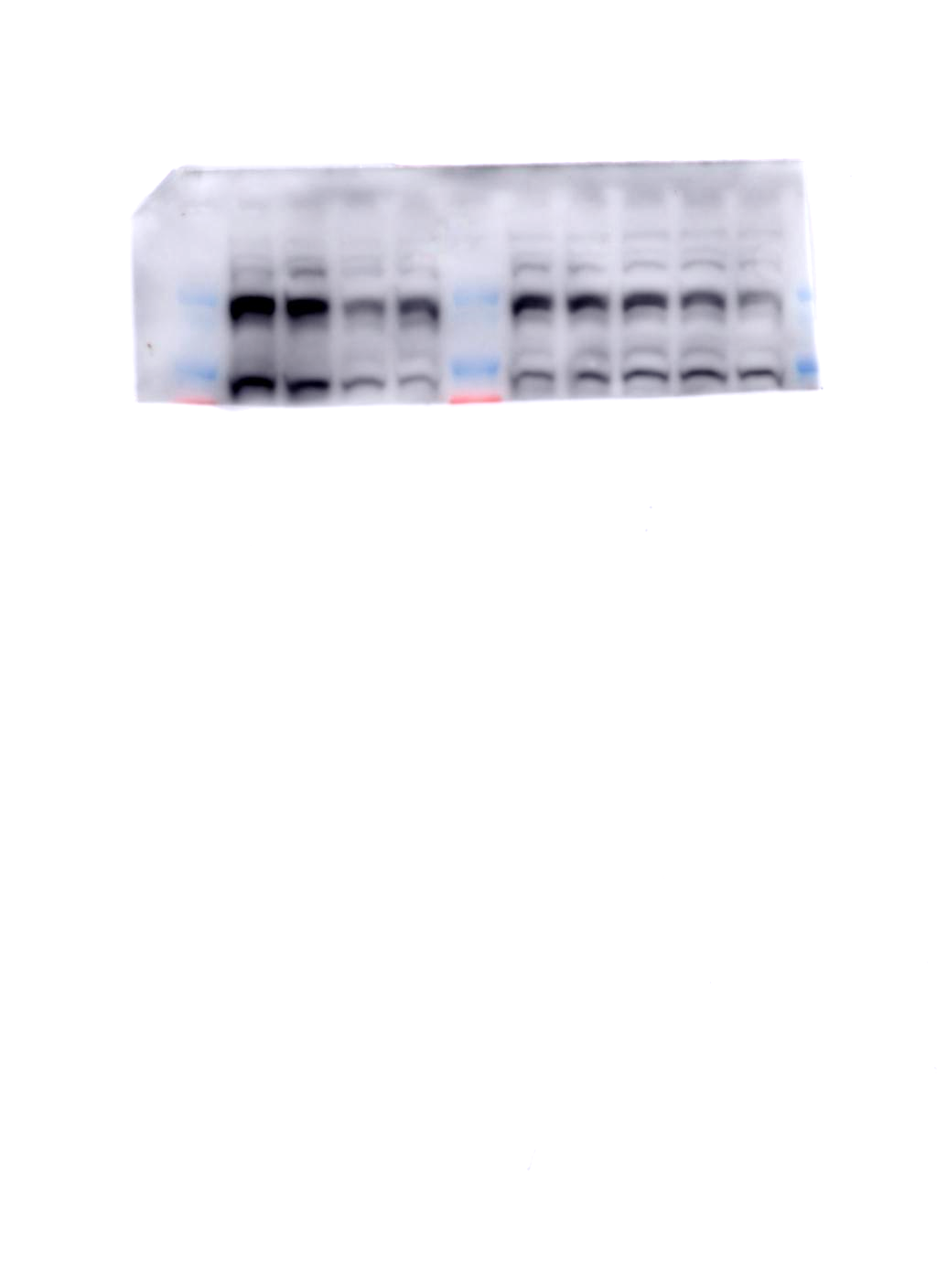


**CAL27**

**Glucose**

**(mM)**

**Glucose**

**(mM)**

**HN6**

**25 5 1 0**

**25 5 1 0**


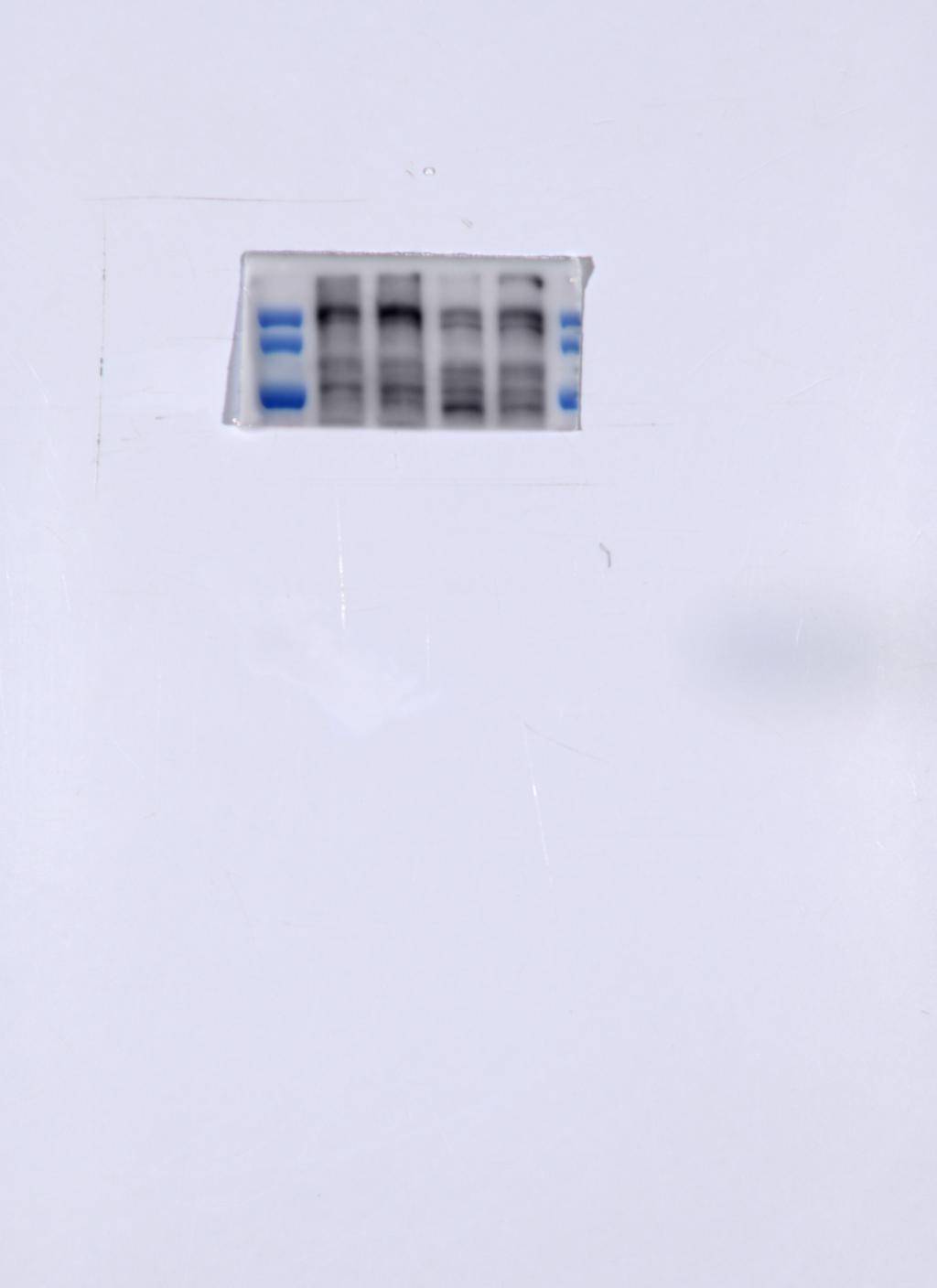


**p-KDM6A**


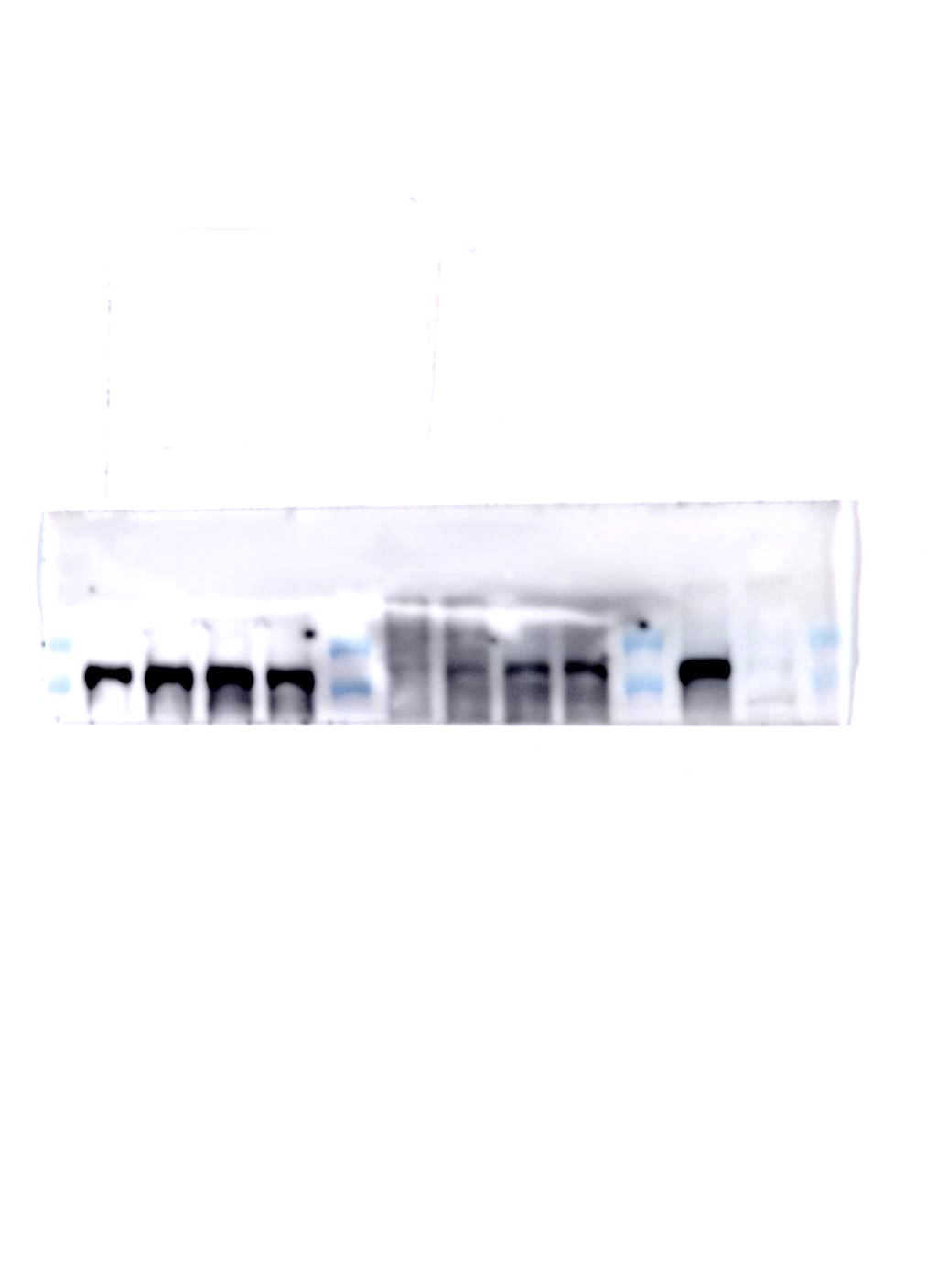


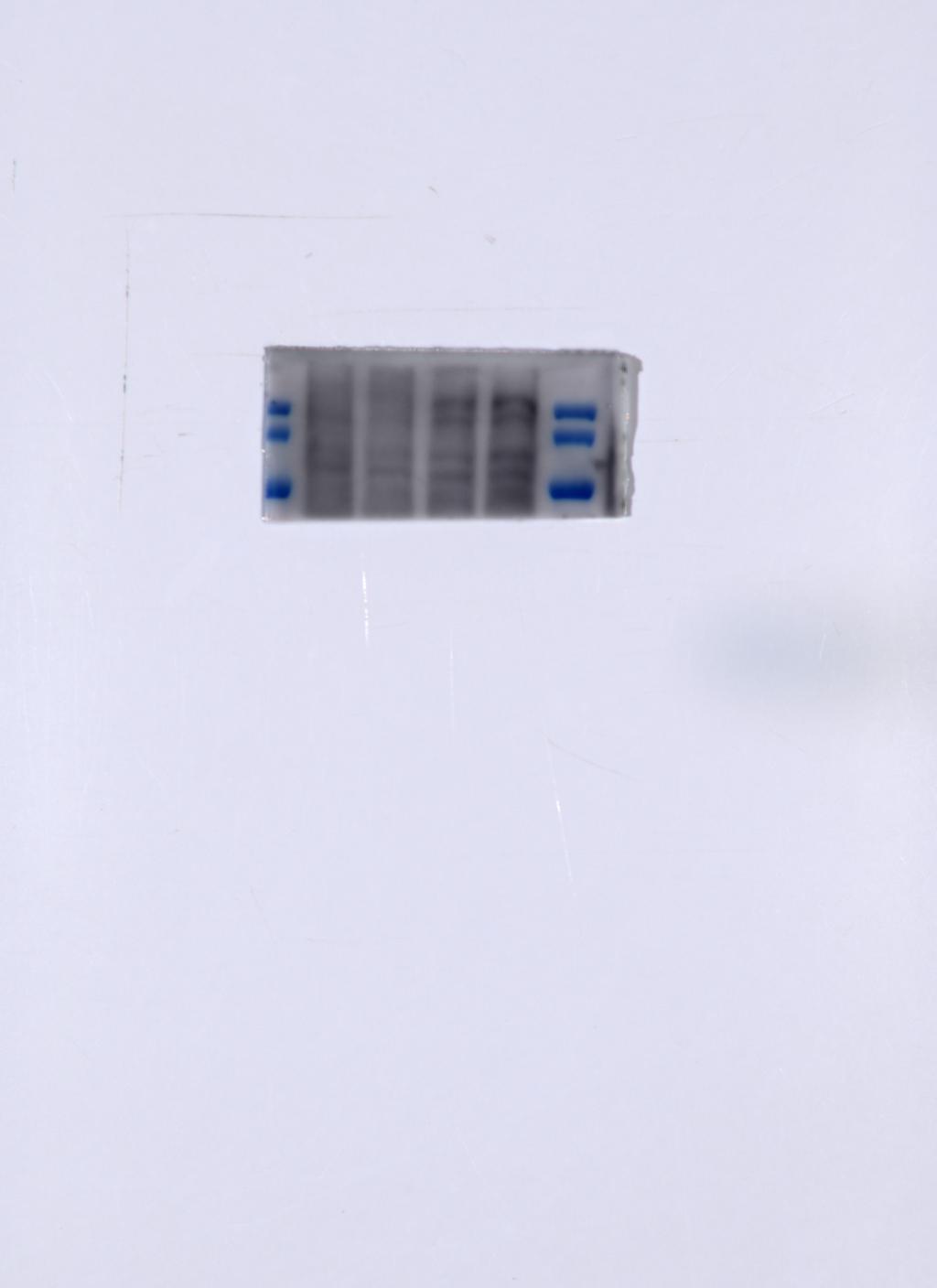


**KDM6A**


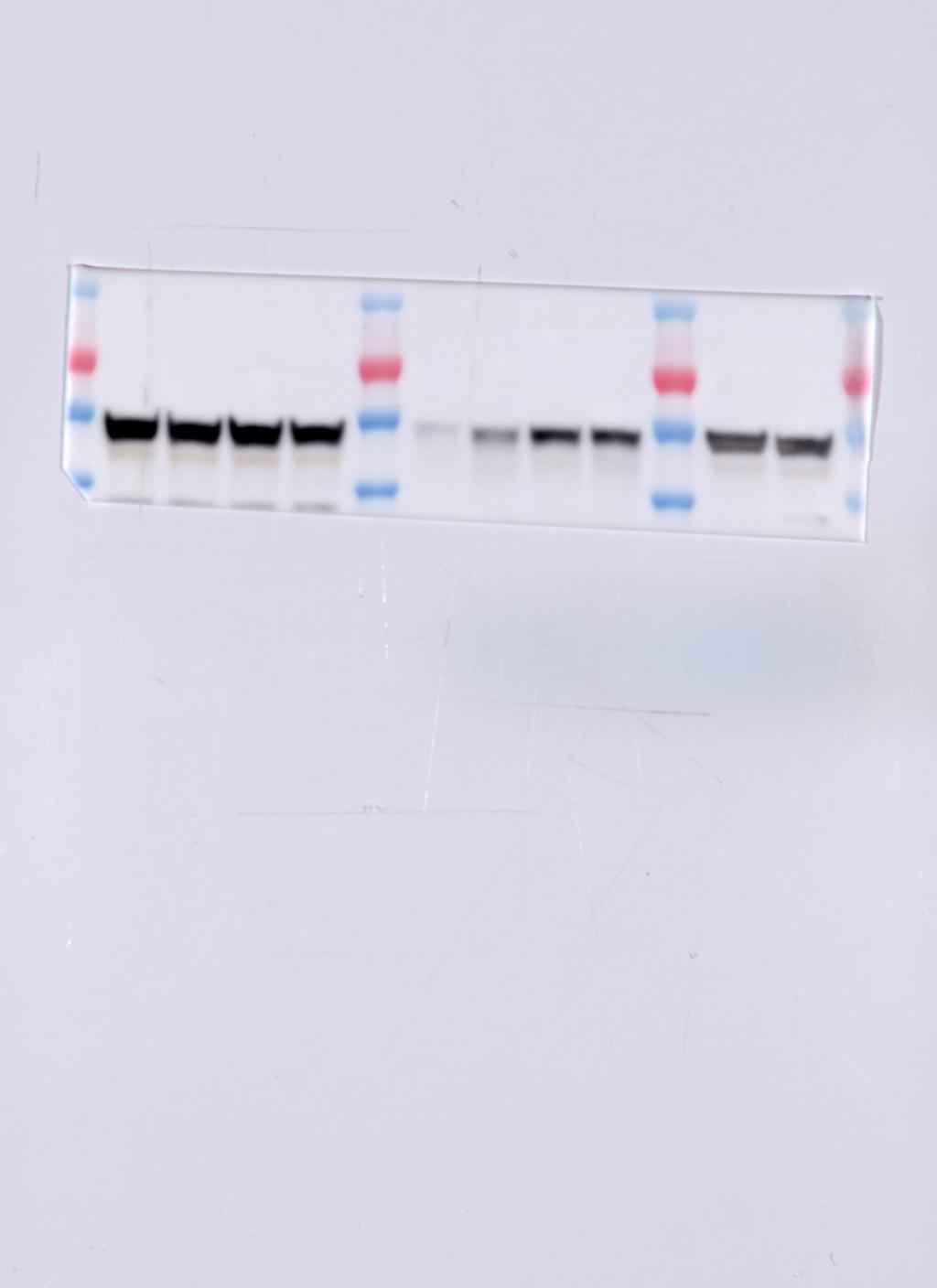


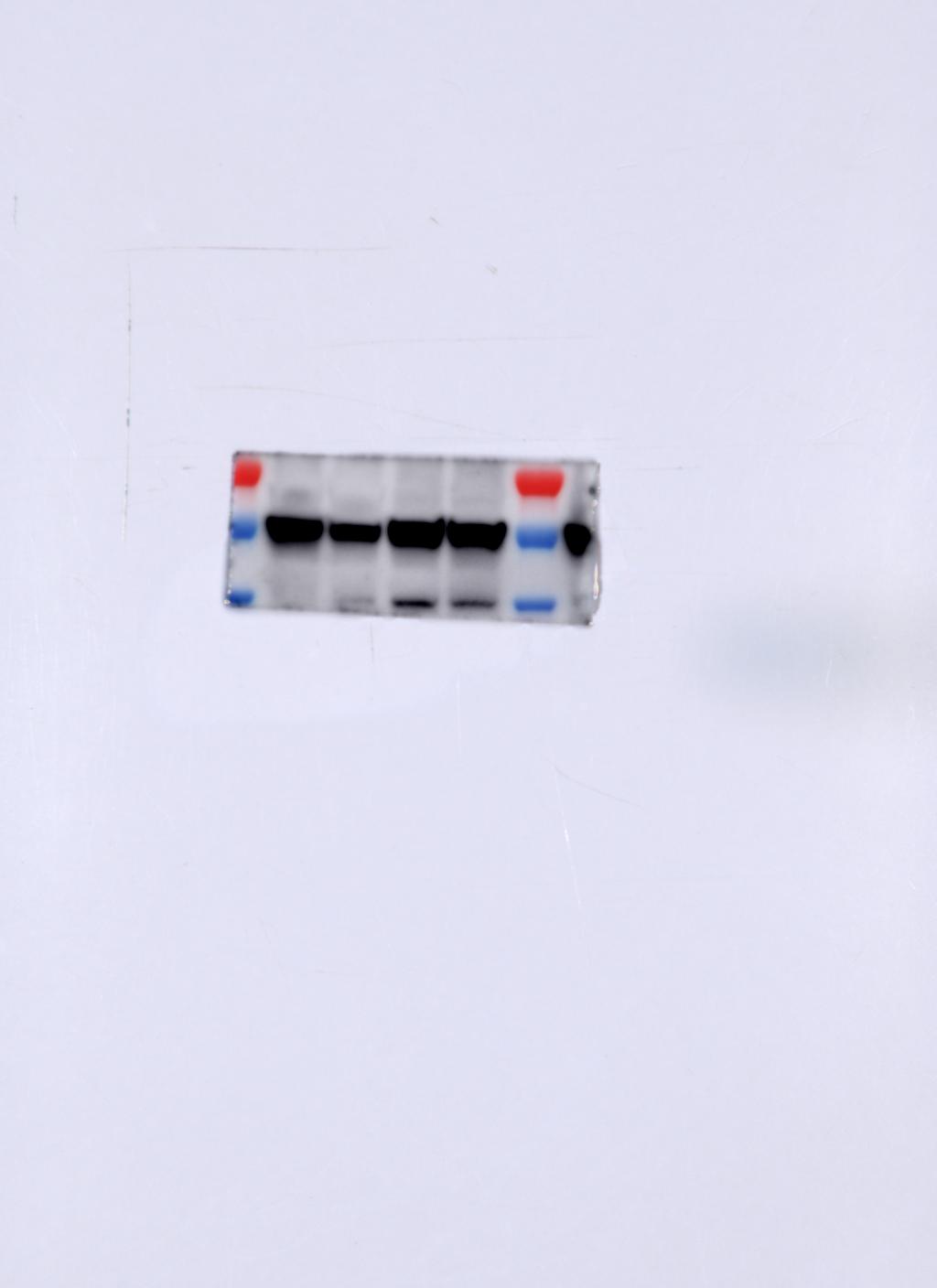


**β-Tubulin**

**Fig. 2D**

**HepG2**

**Glucose**

**(mM)**

**B16**

**25 5 1 0**

**Glucose**

**(mM)**

**p-KDM6A**

**KDM6A**

**β-Tubulin**

**25 5 1 0**


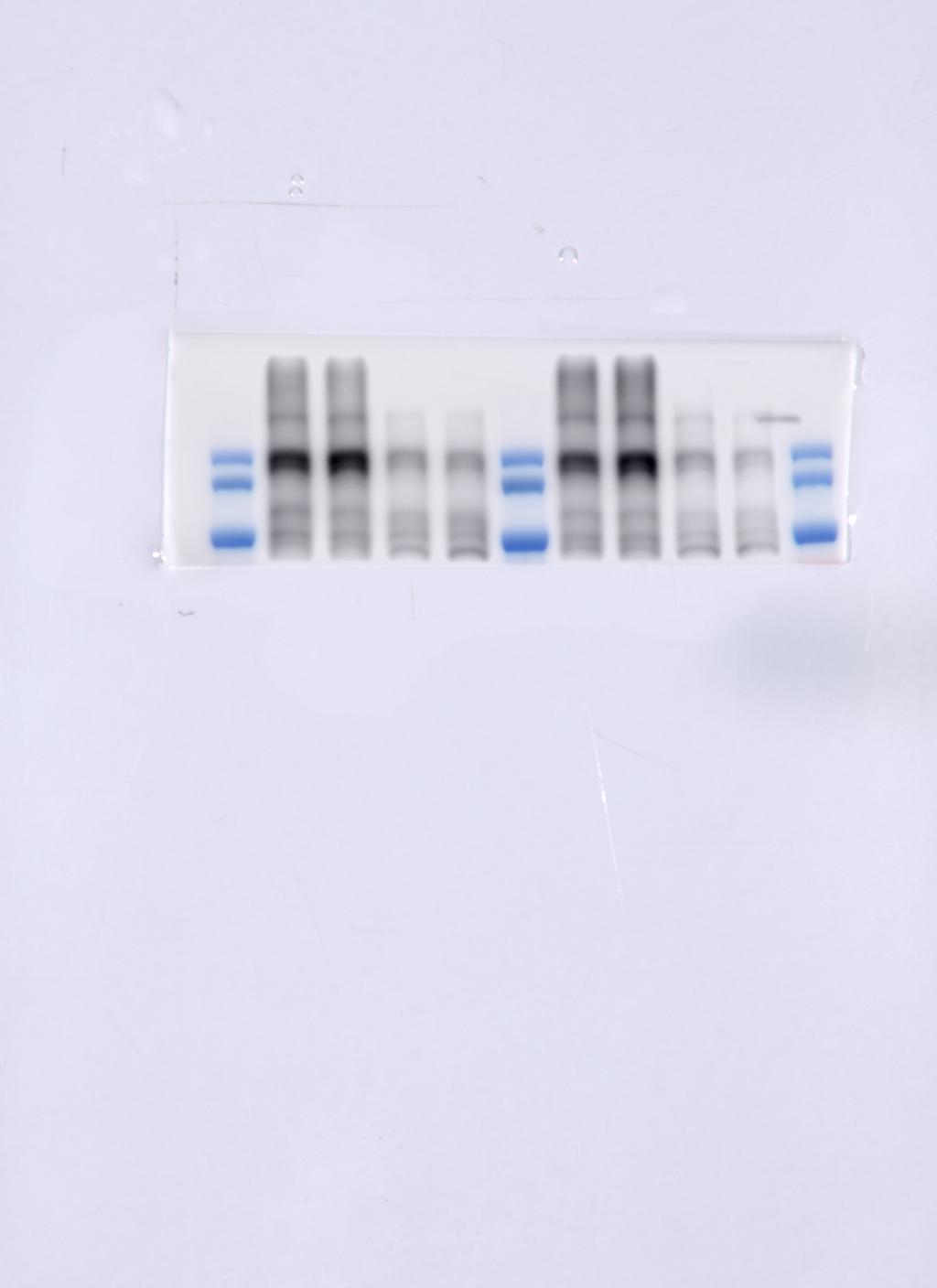

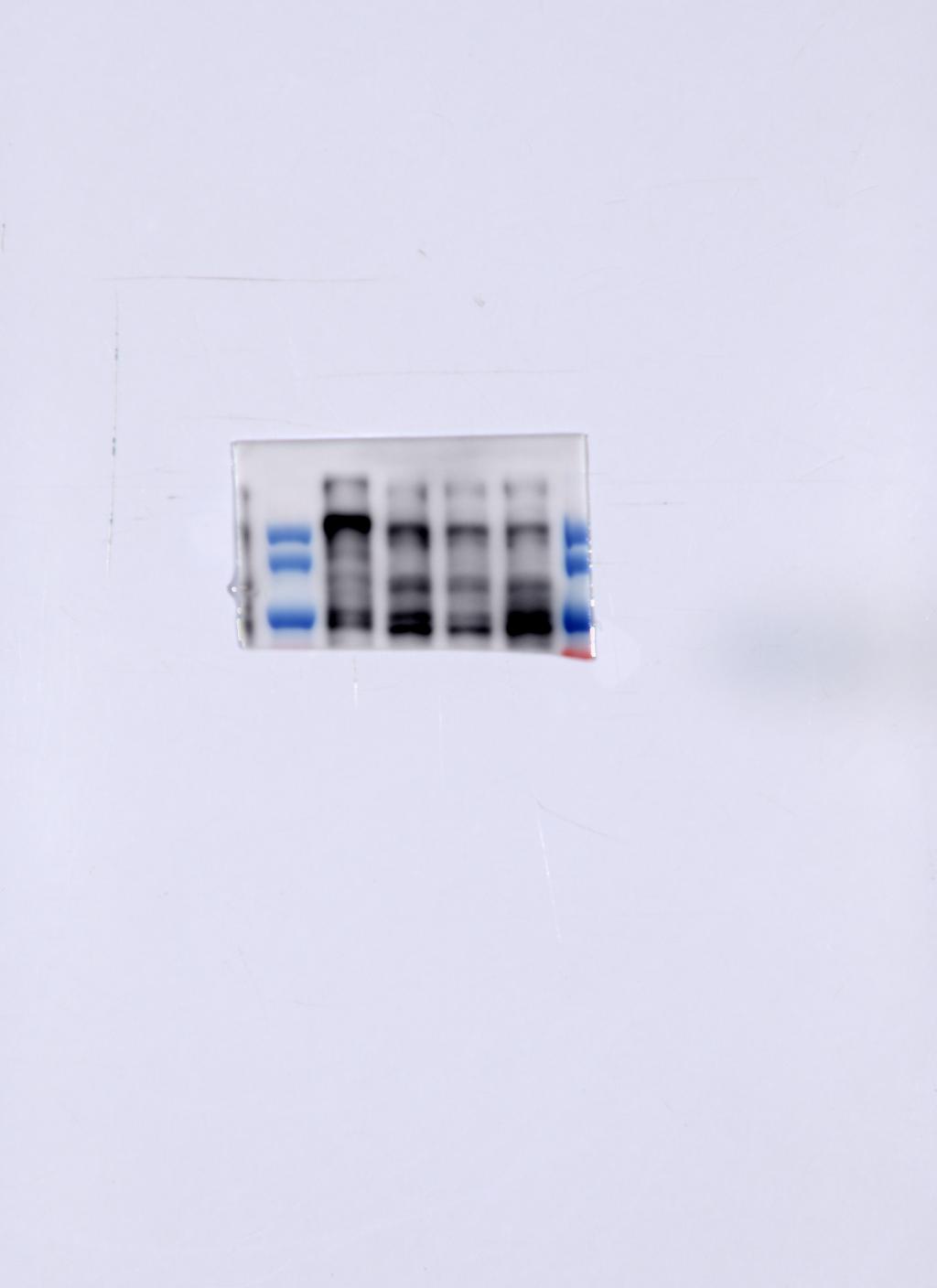


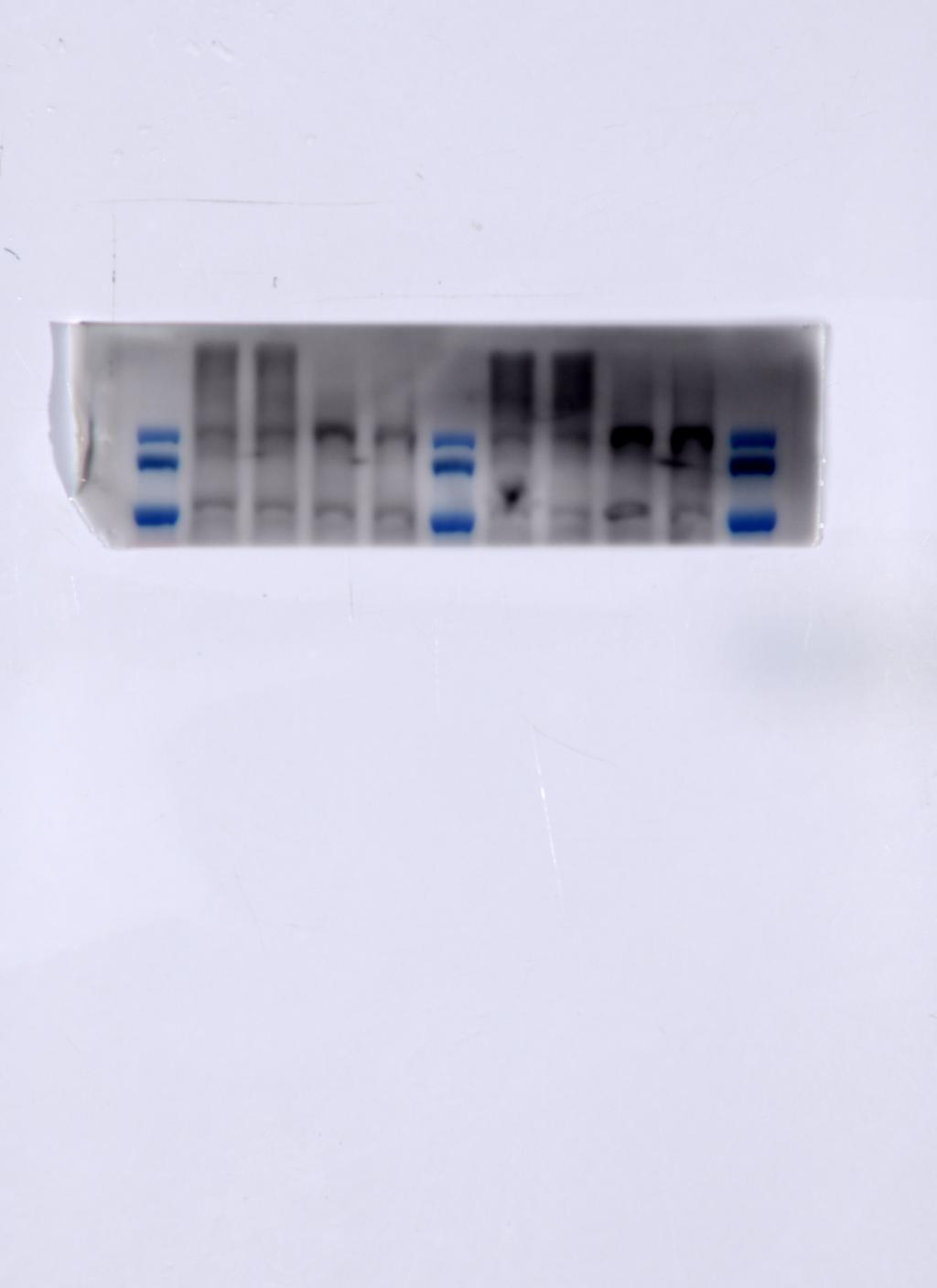

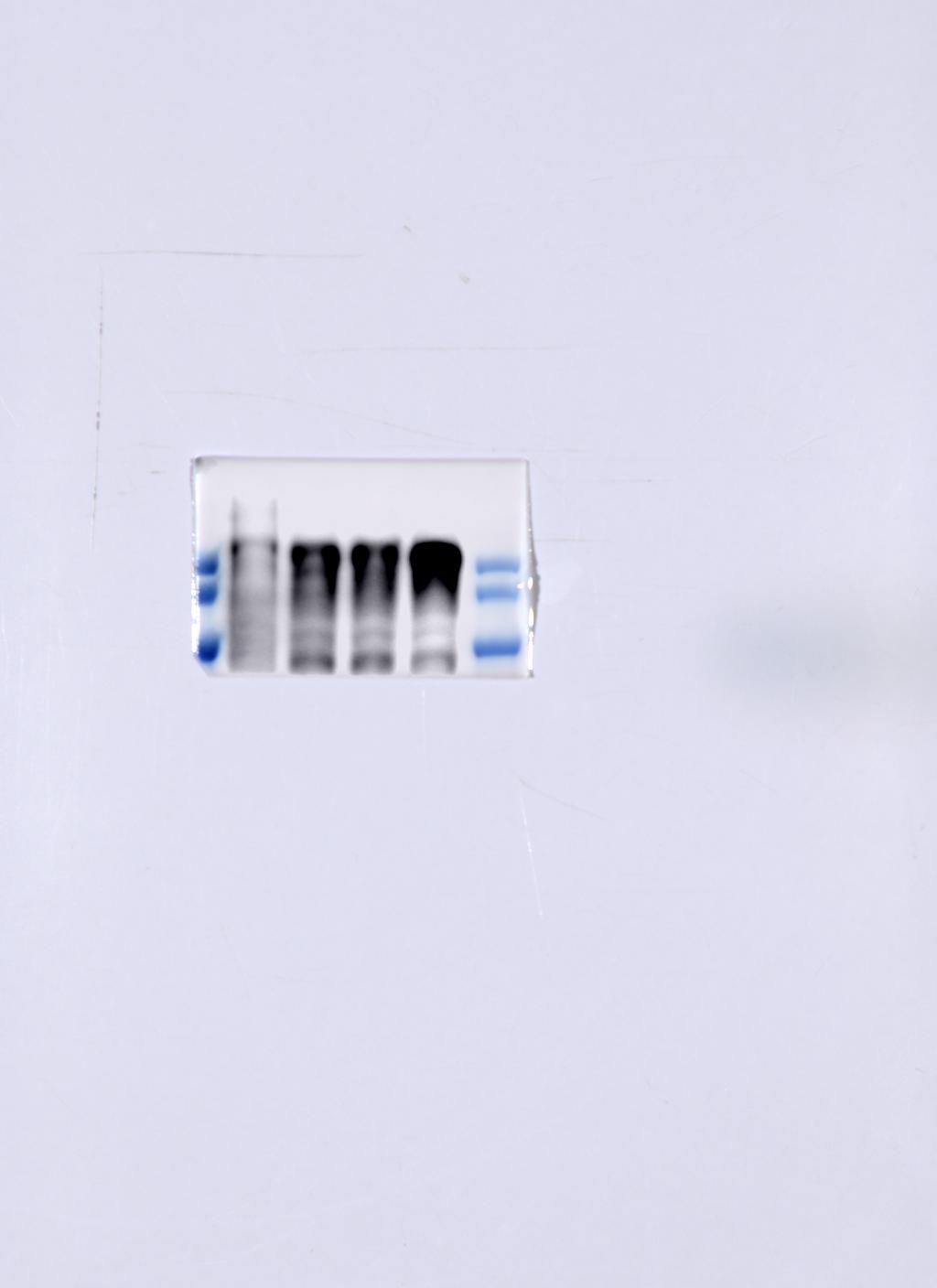


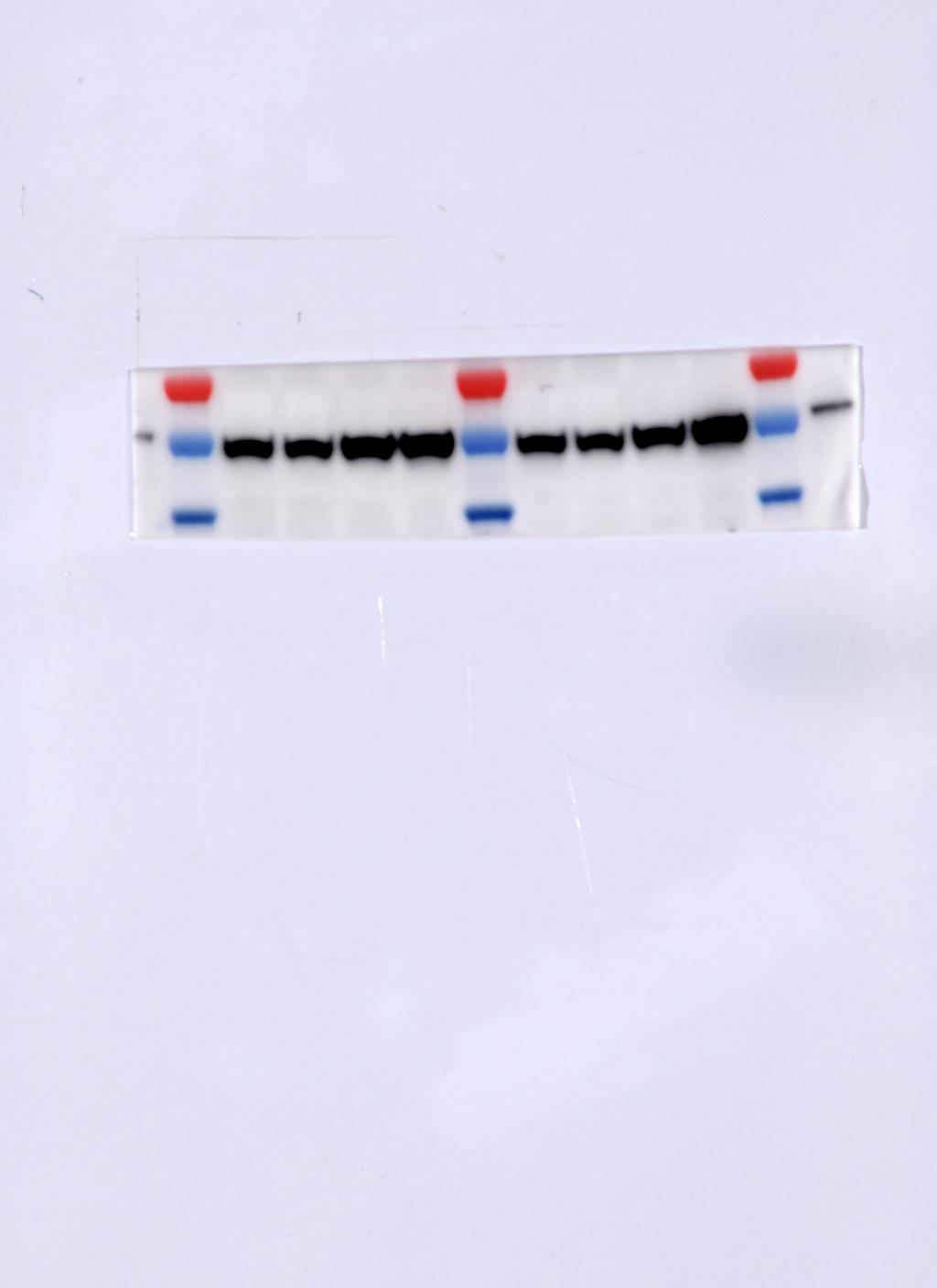

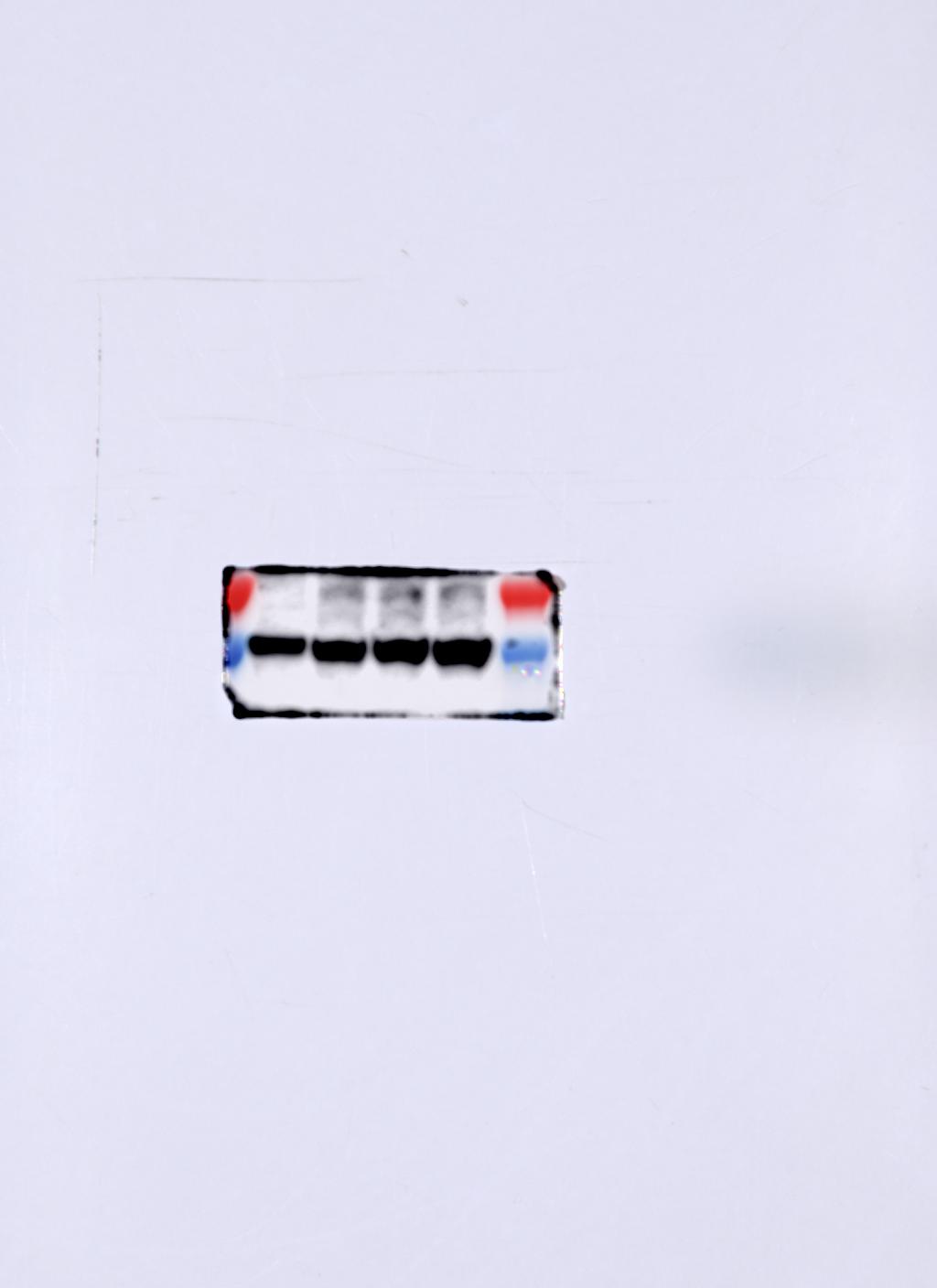


**Fig. 2F**

HN6

Vector

WT

S829A

S829D

β-ACTIN

**
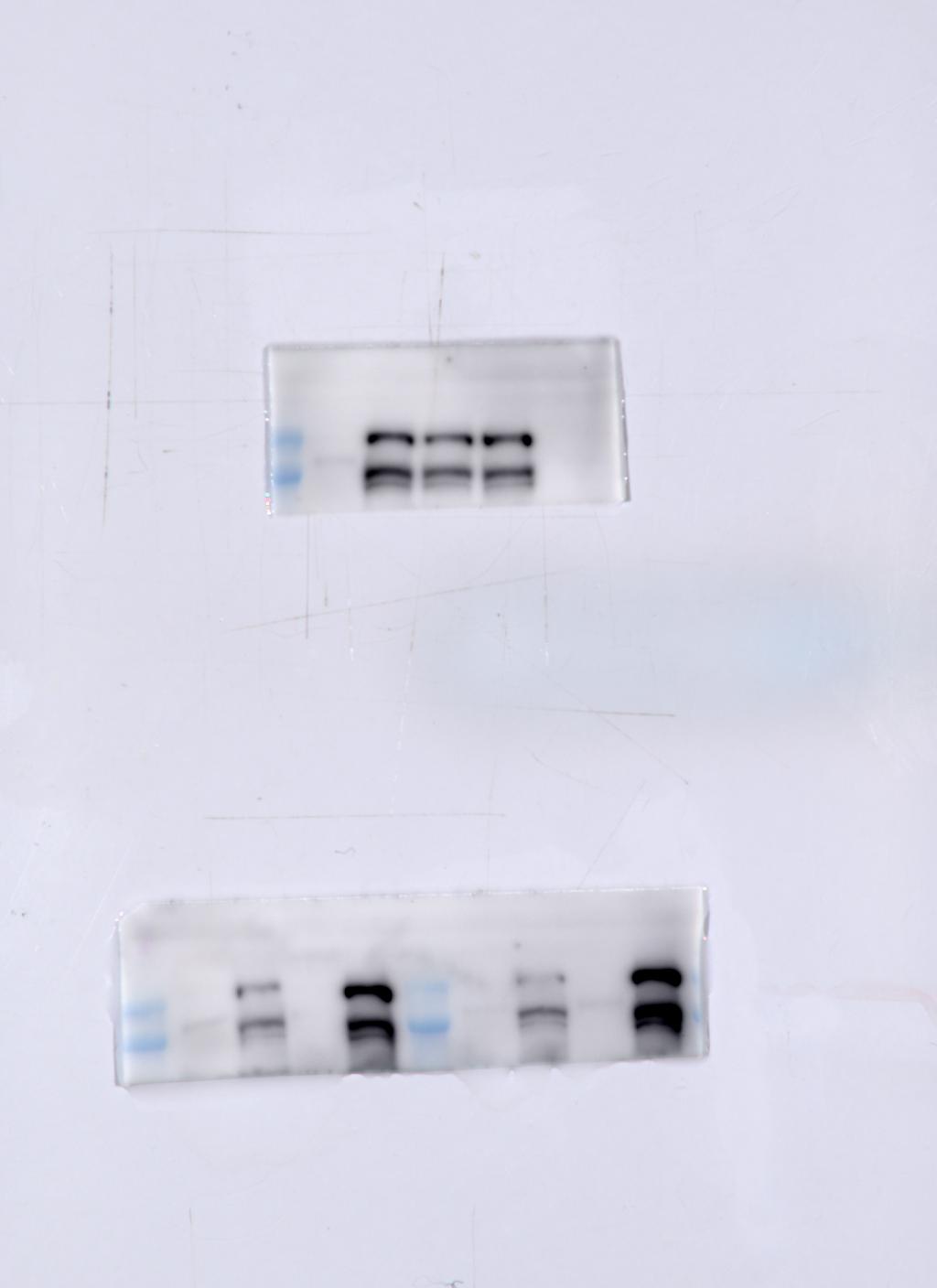
**

p-KDM6A

**
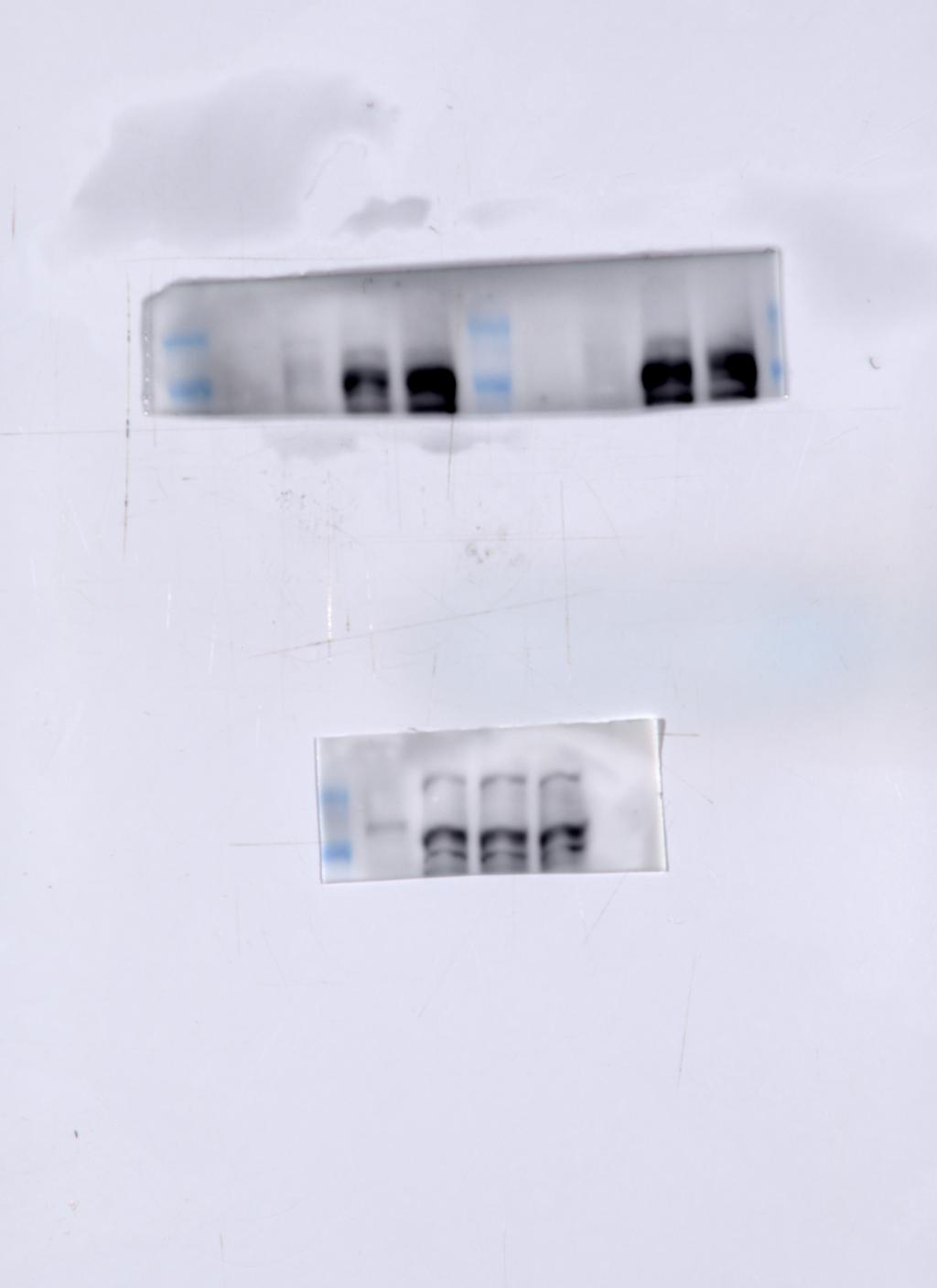
**

KDM6A


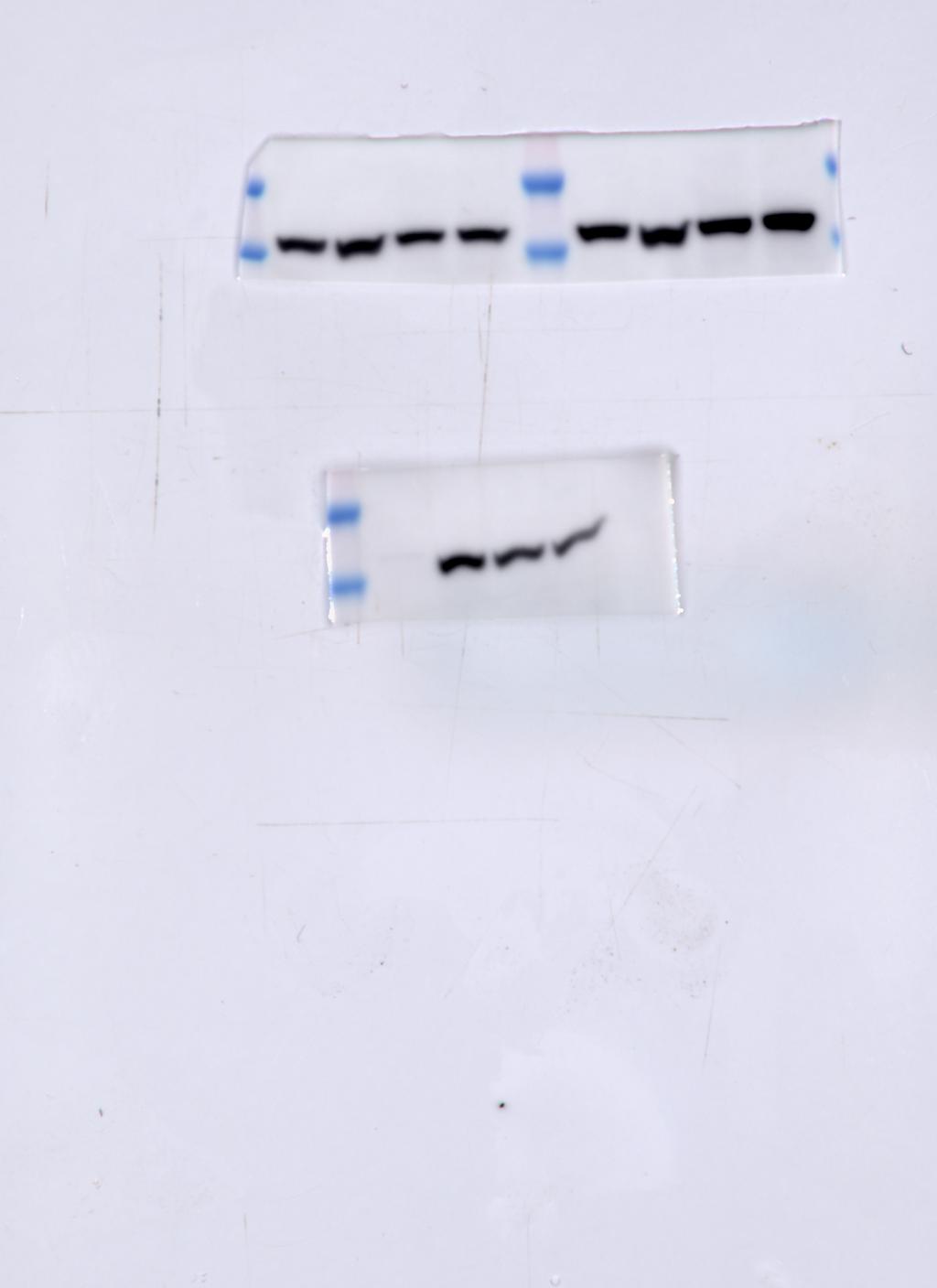


p-KDM6A

KDM6A

Cal27

β-ACTIN

Vector

WT

S829A

S829D

**
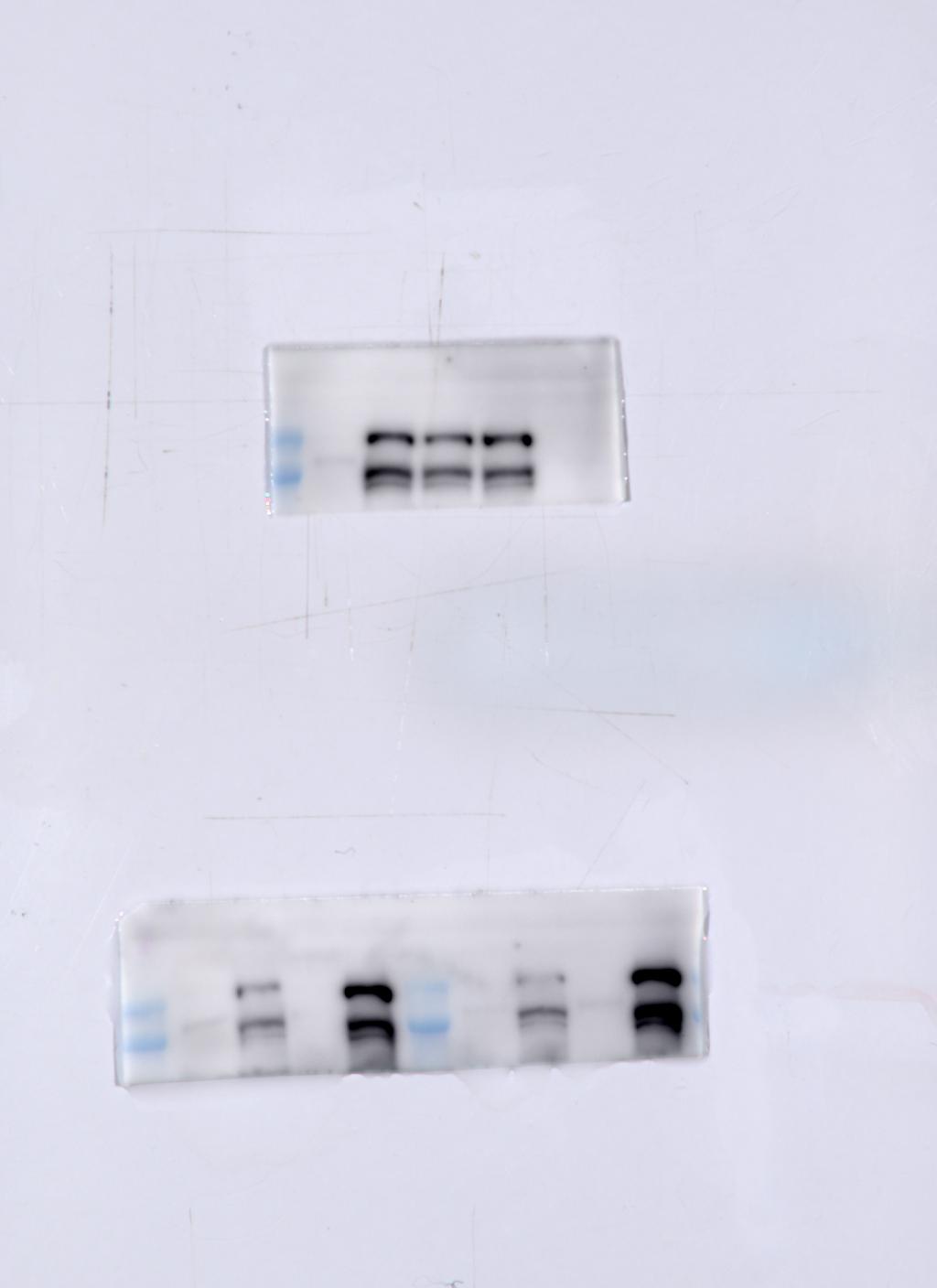
**


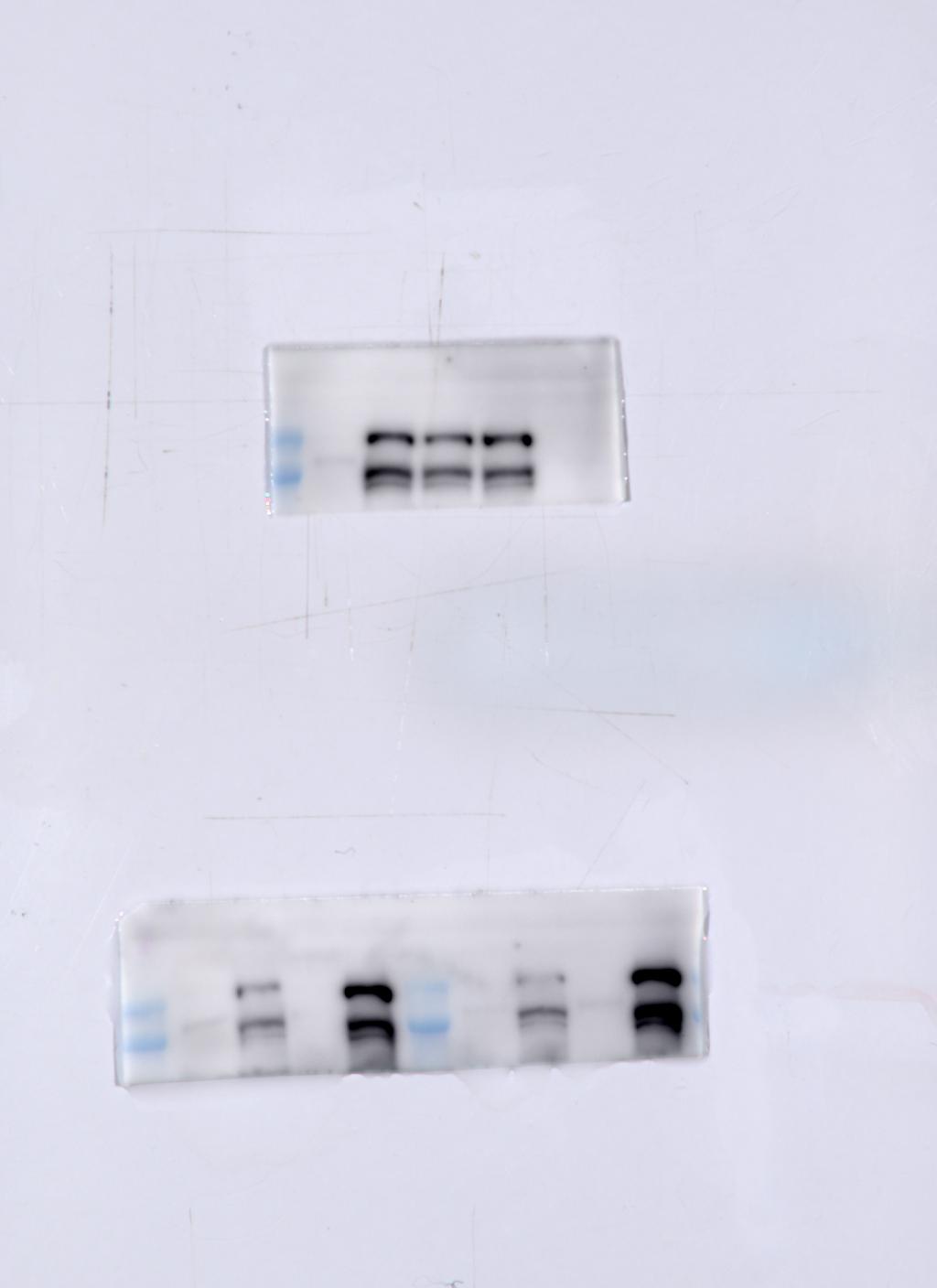


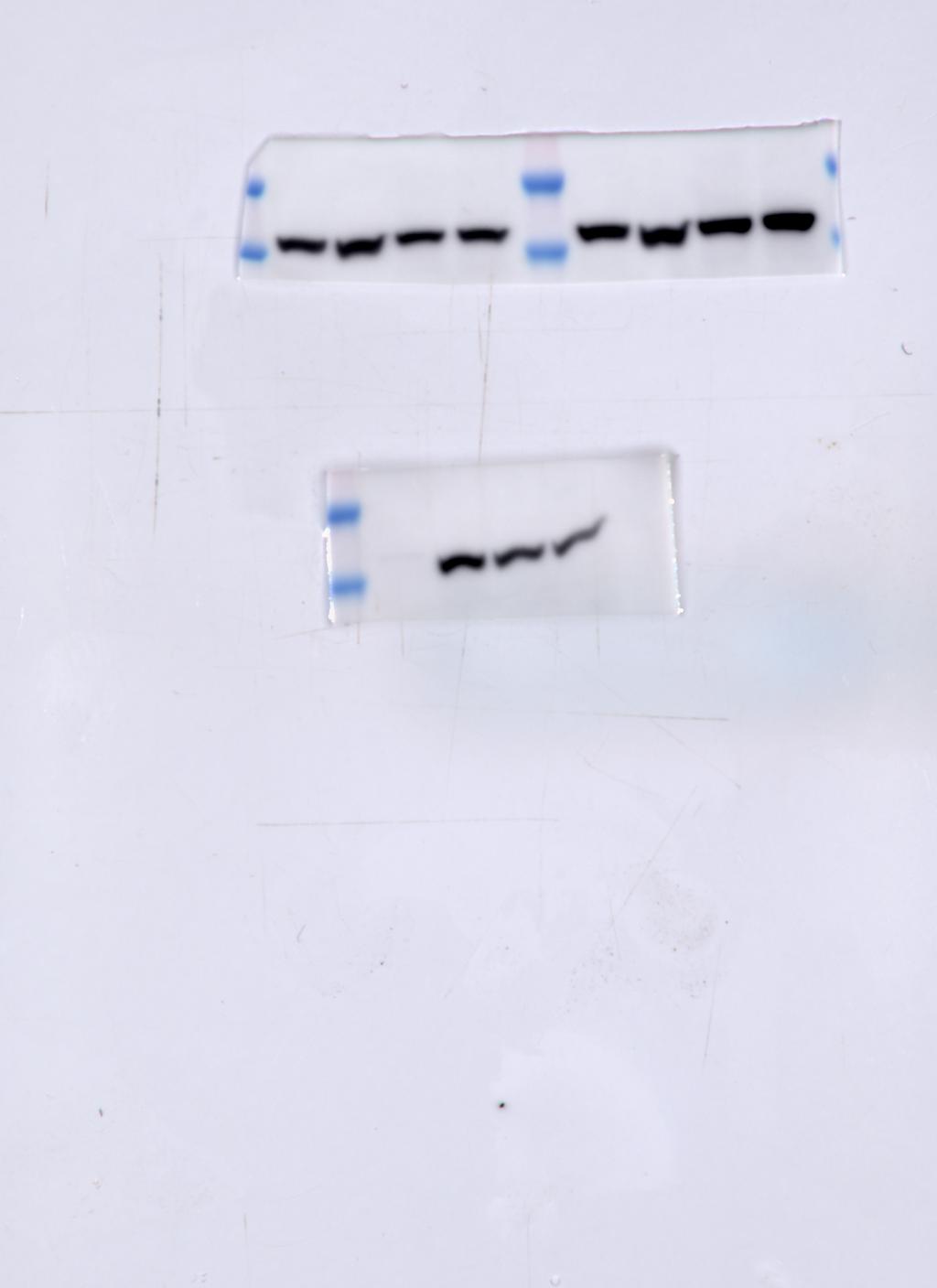


**Fig. 3B**

Cytoplasm

Nucleus

KDM6A

**
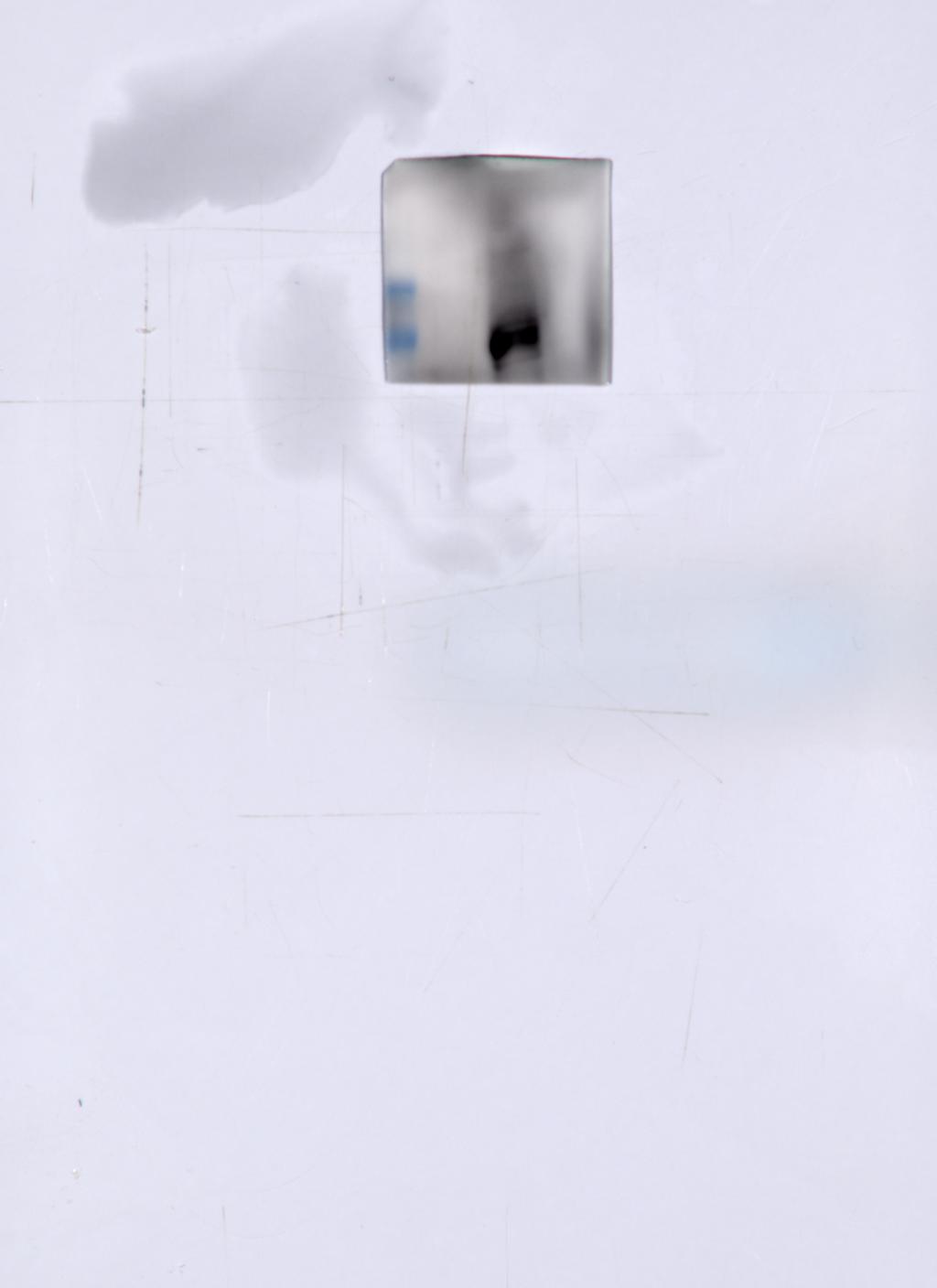
**

**
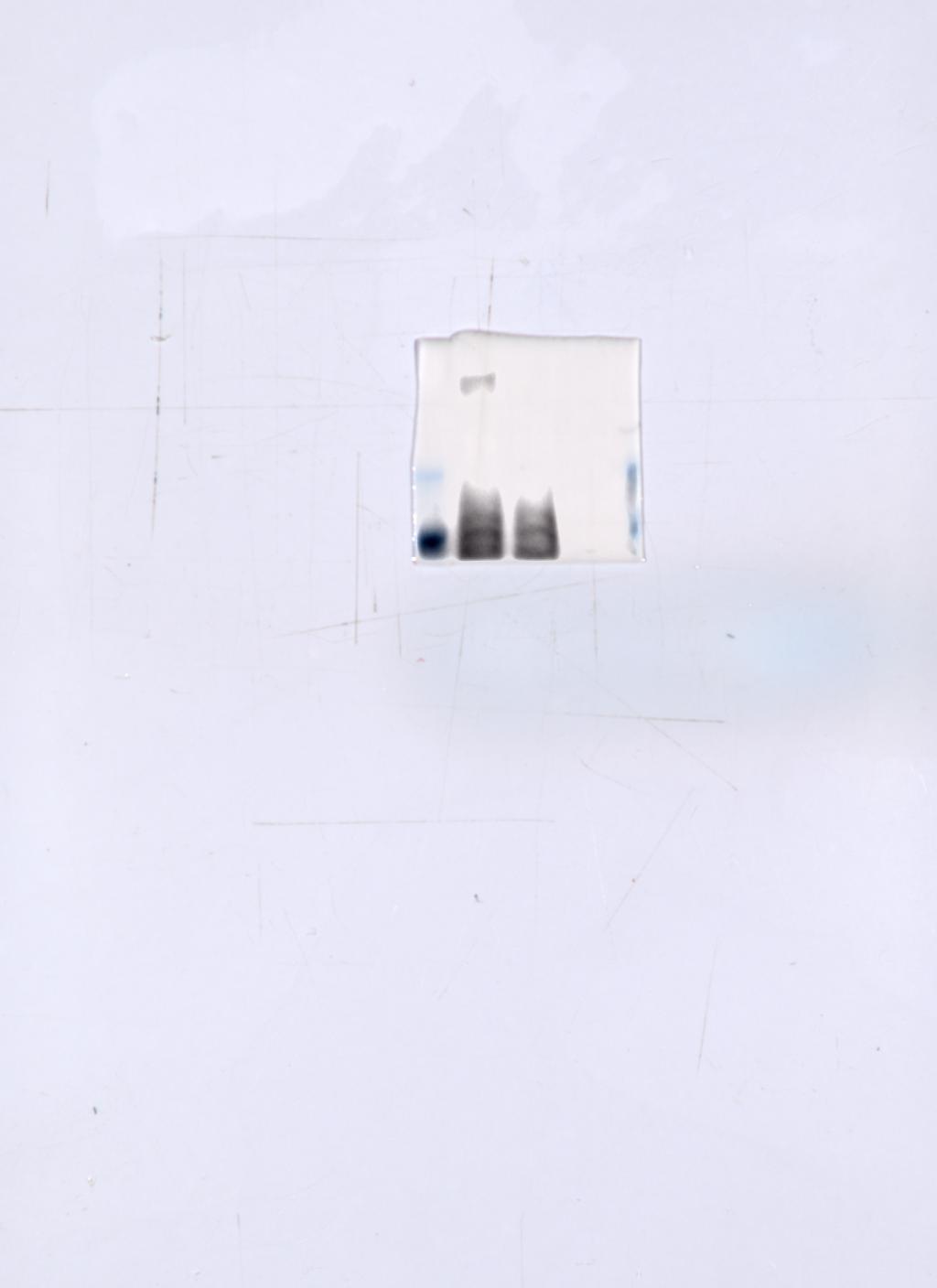
**

p-KDM6A

**
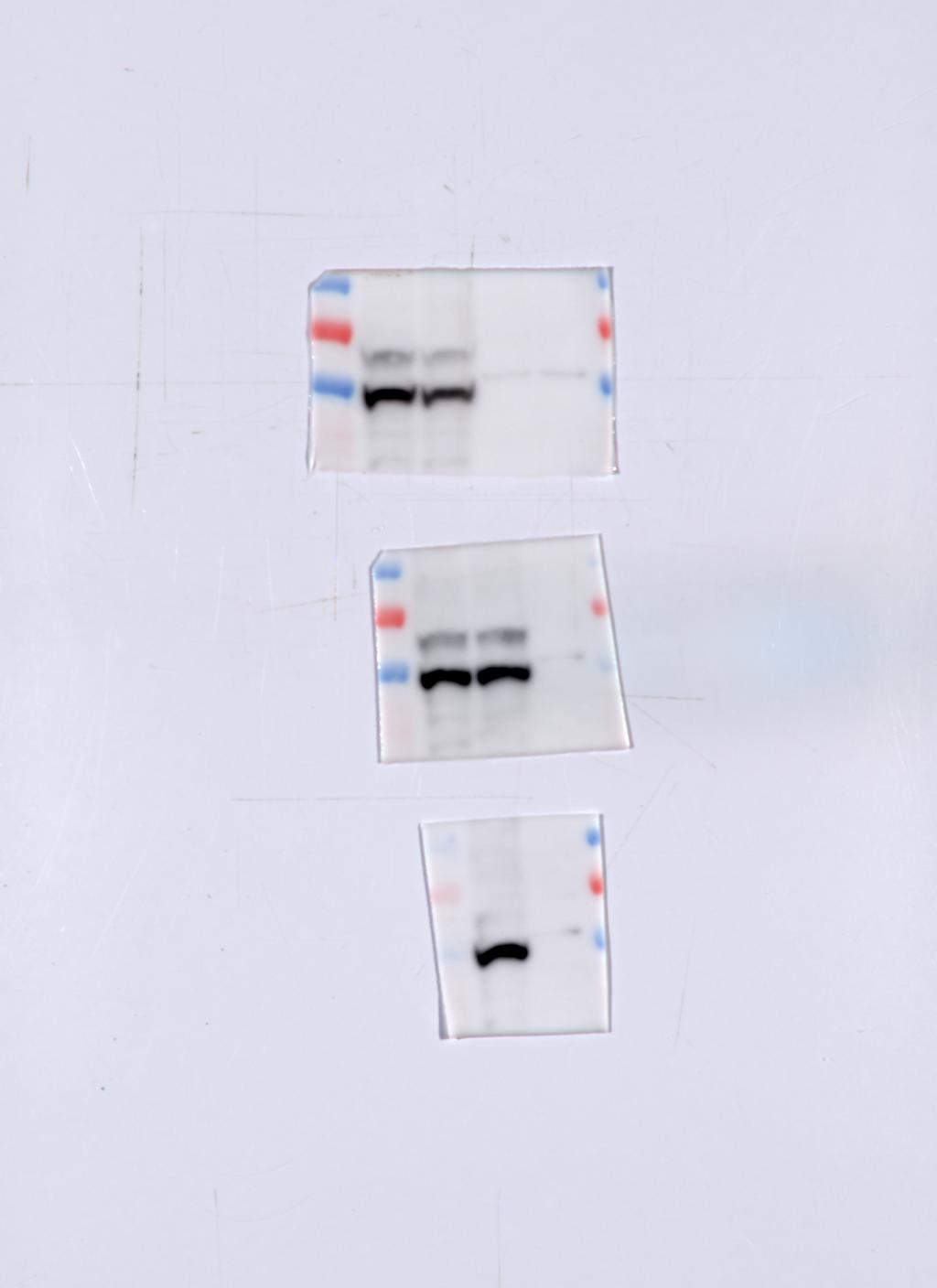
**

TUBLIN

**
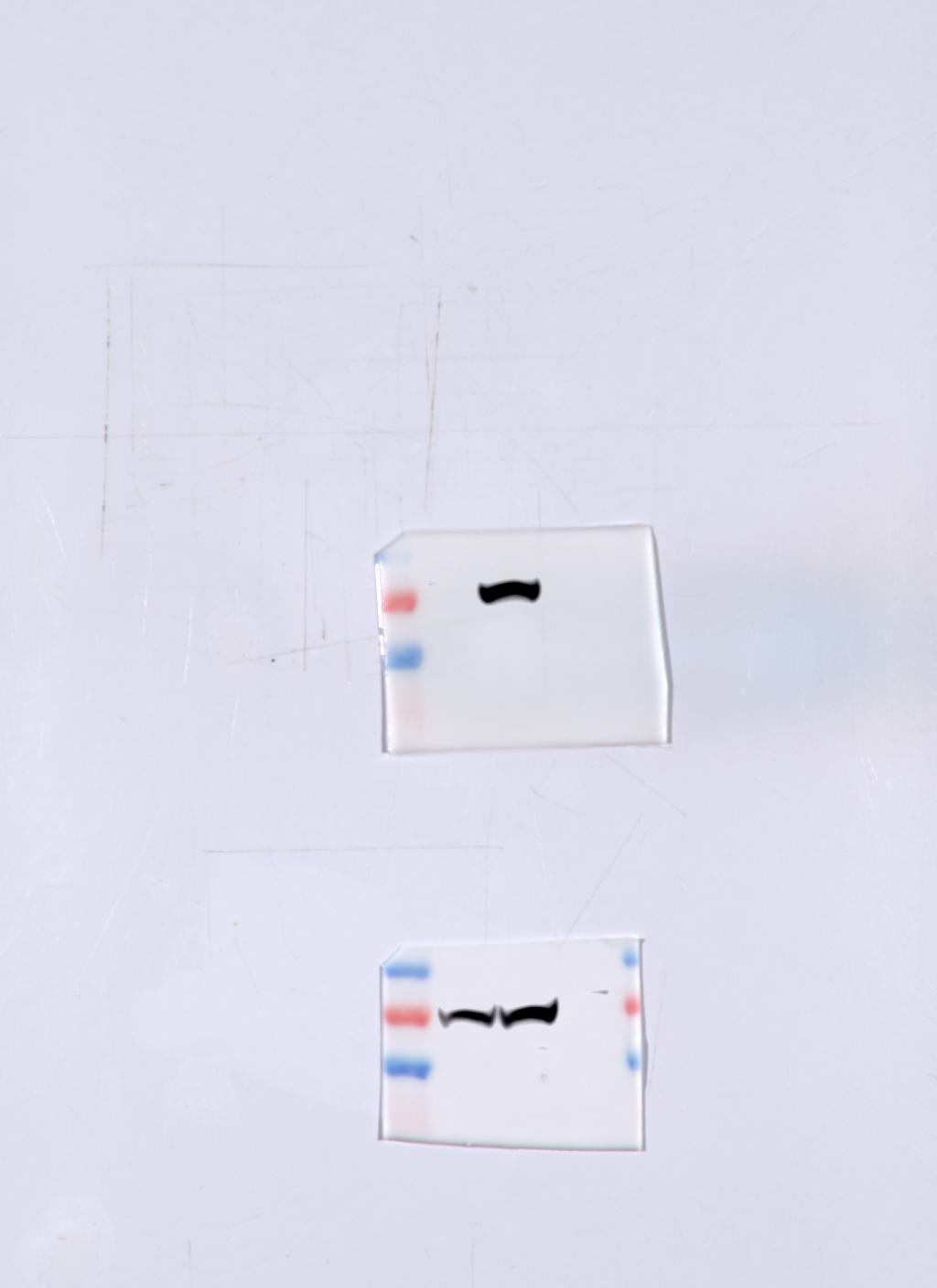
**

LAMIN B1

**Fig. 3D**

IP:KDM6A

IP:SFN

**
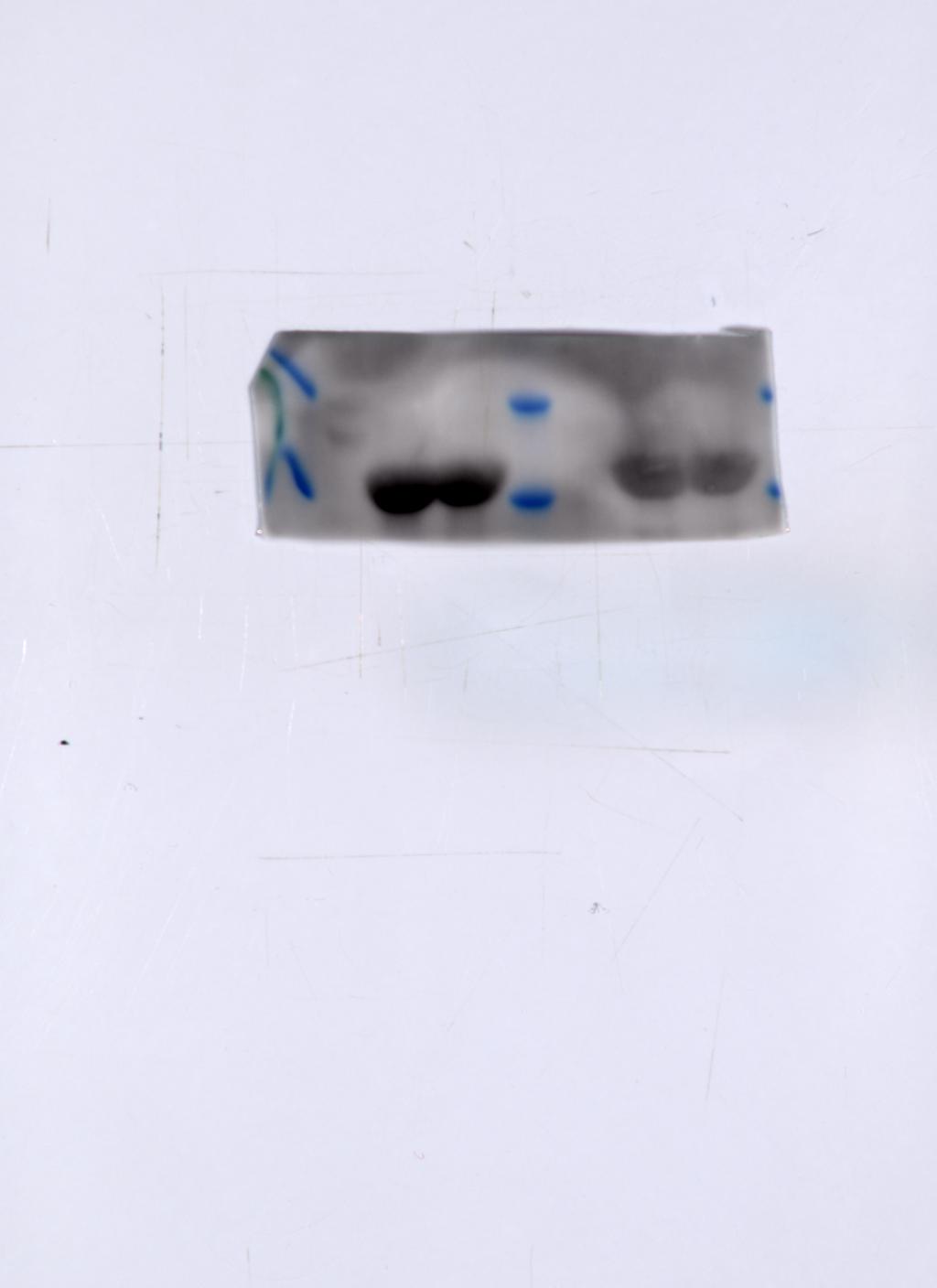
**

IP:SFN

IgG

IP:KDM6A

Input

**
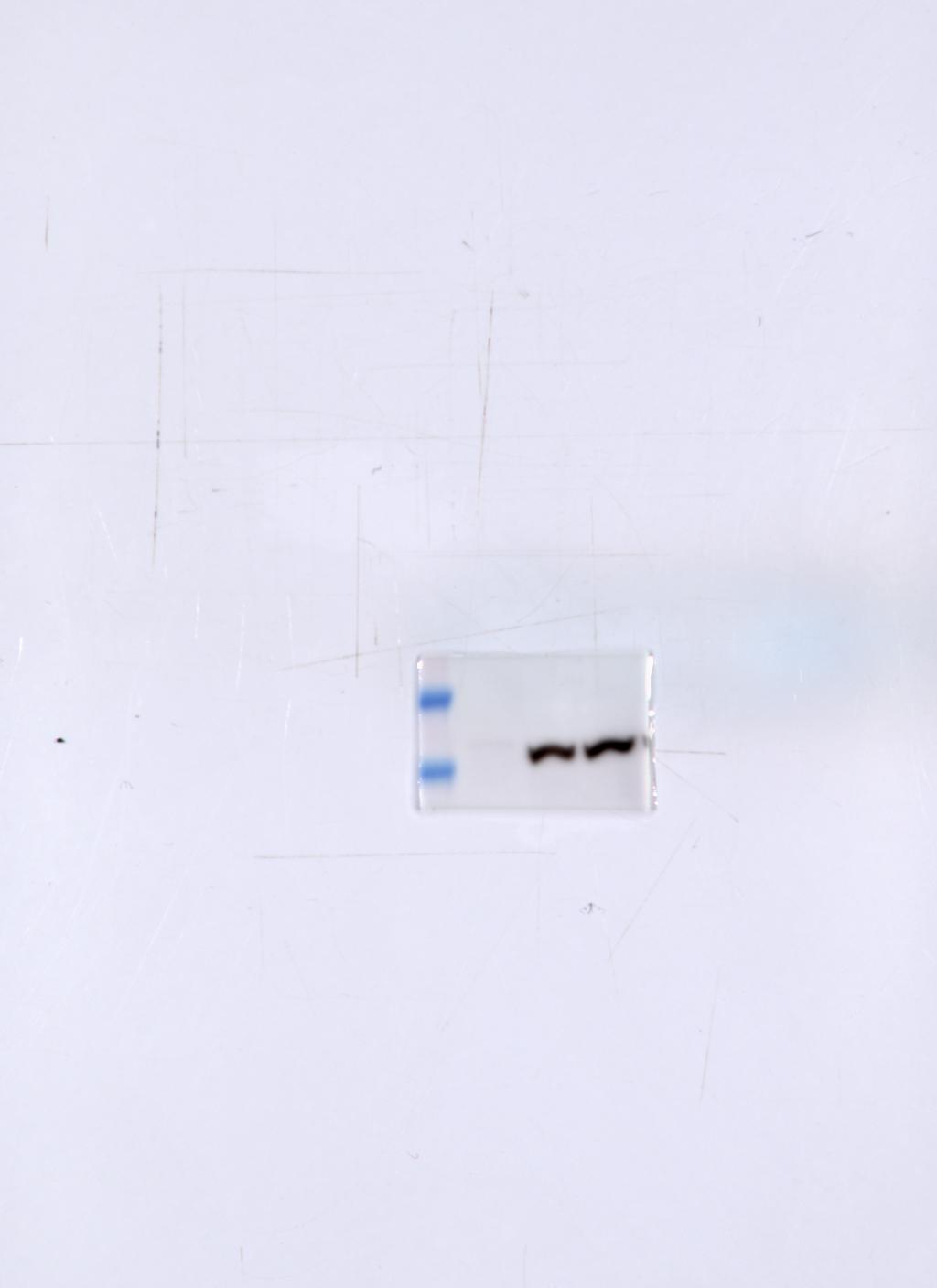
**

IgG

Input

**
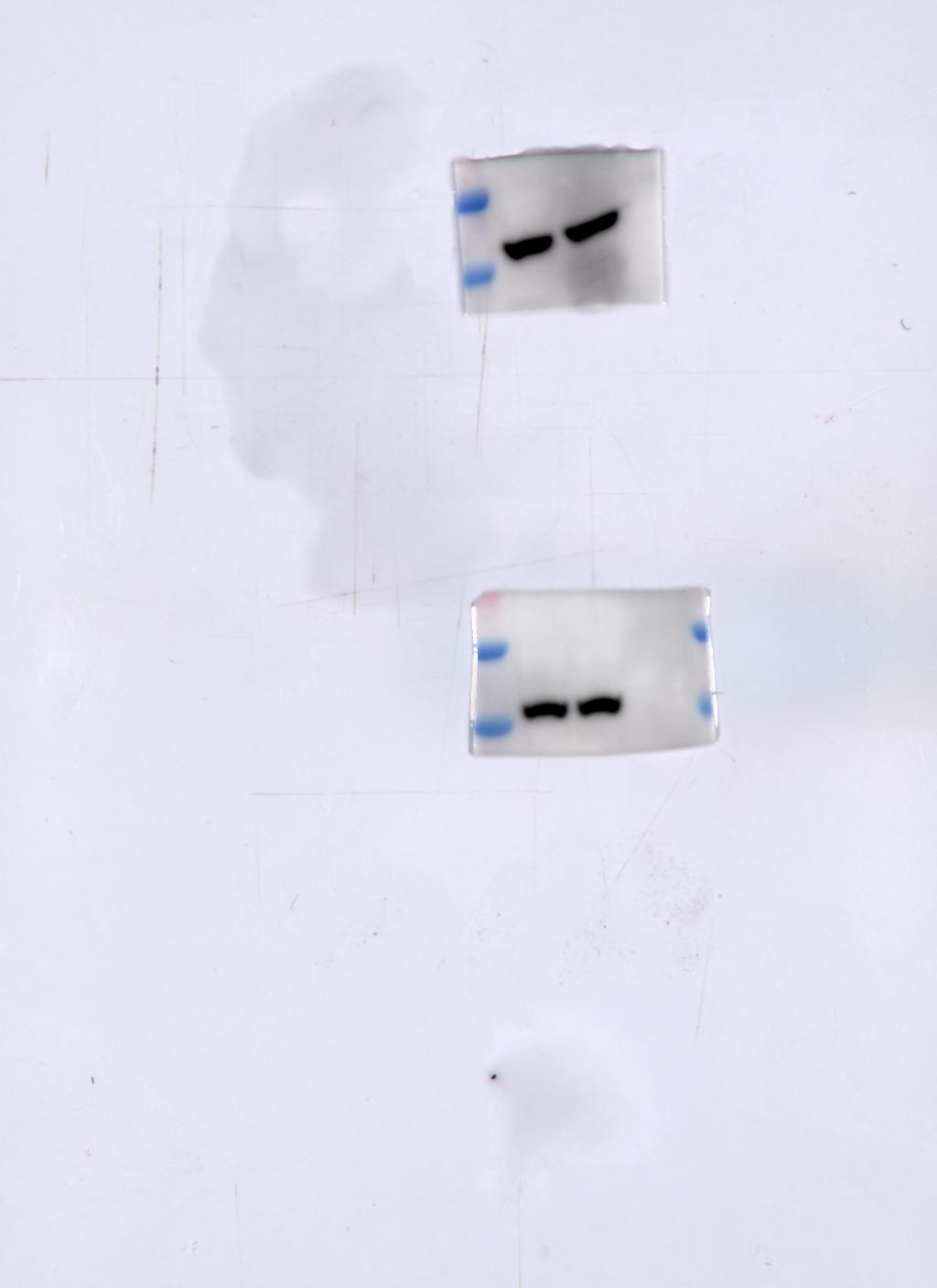
**

IgG

IgG

**
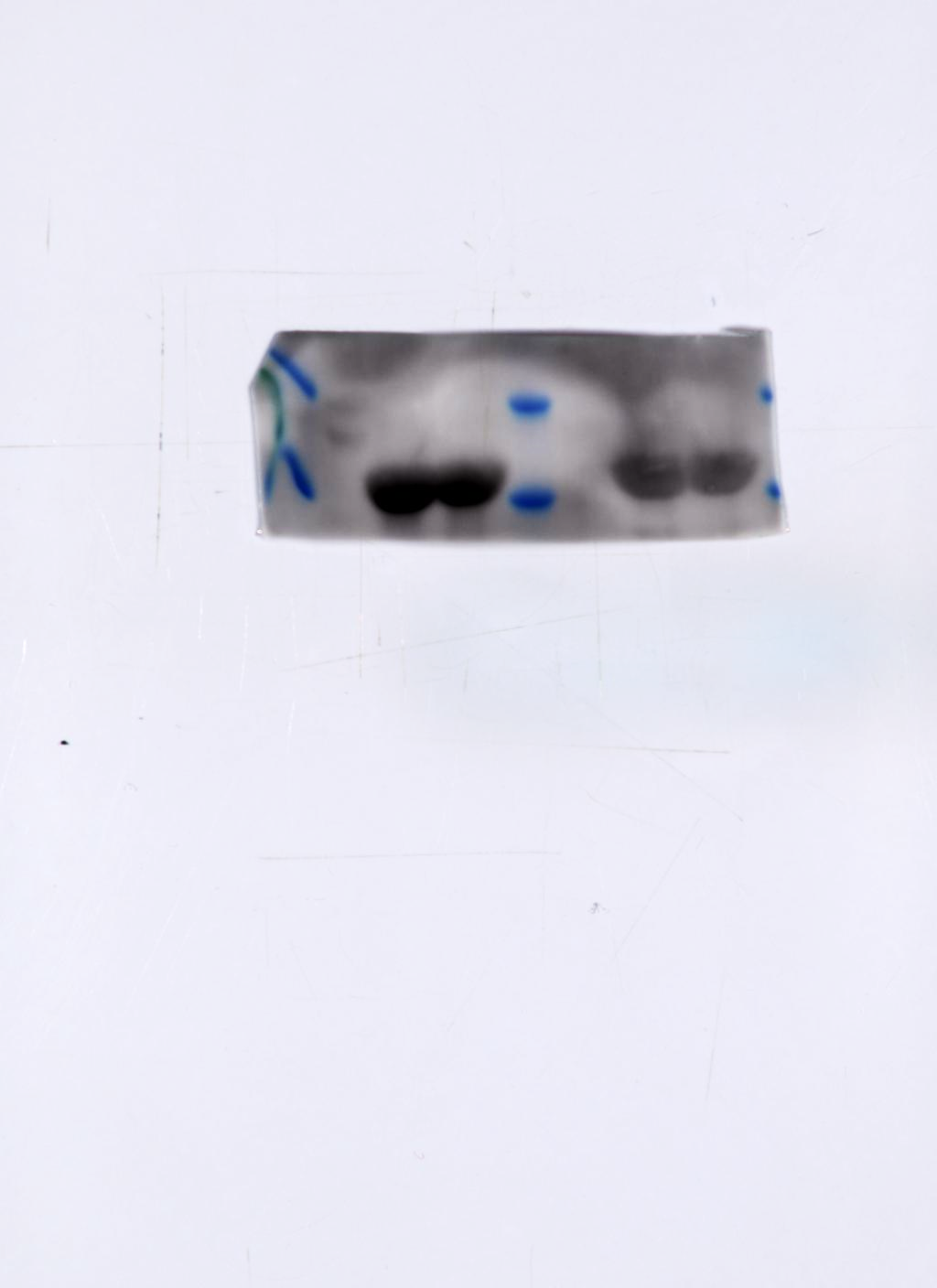
**

**
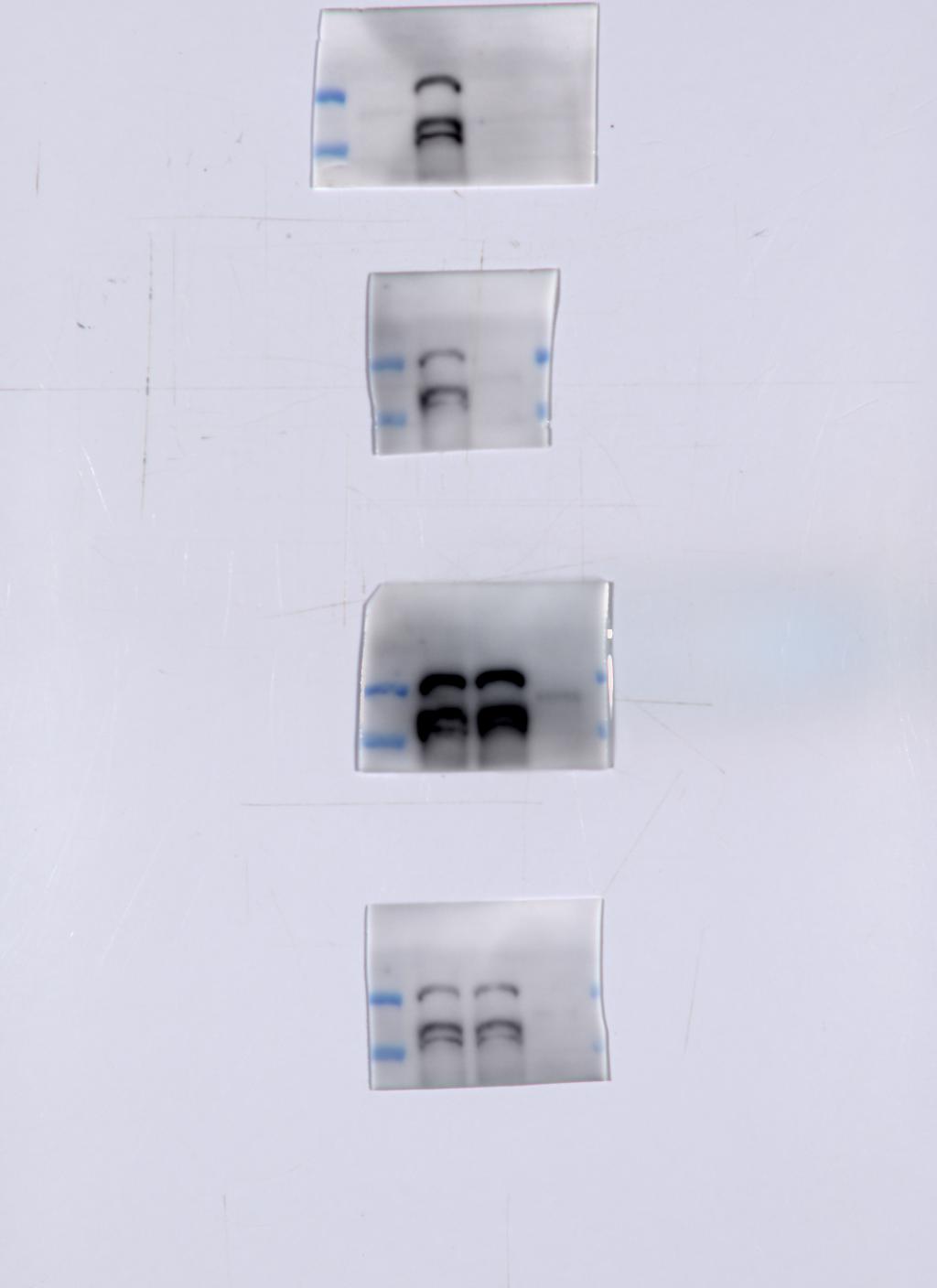
**

SFN

KDM6A

**
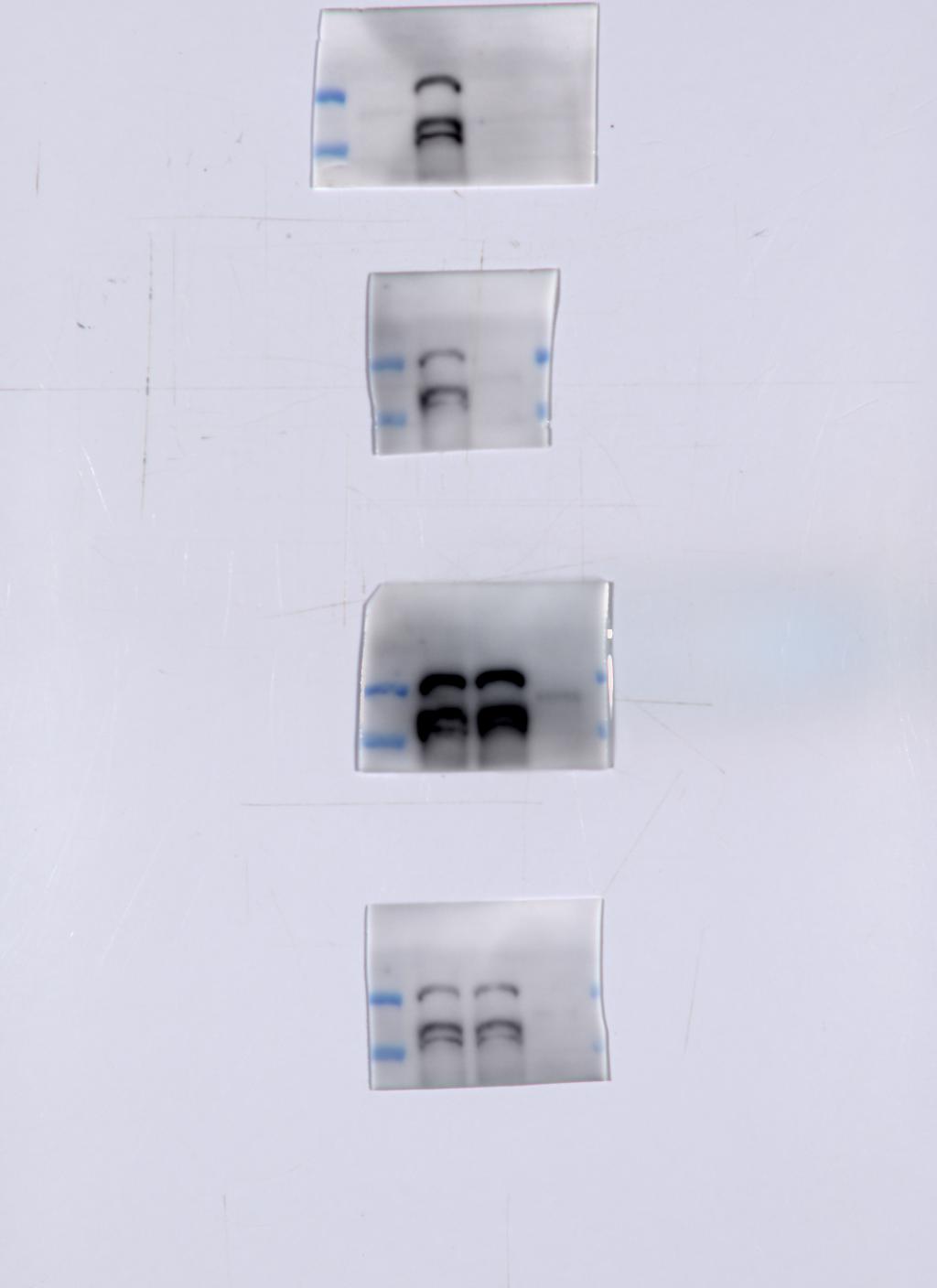
**

KDM6A

SFN

**Fig. 3E**


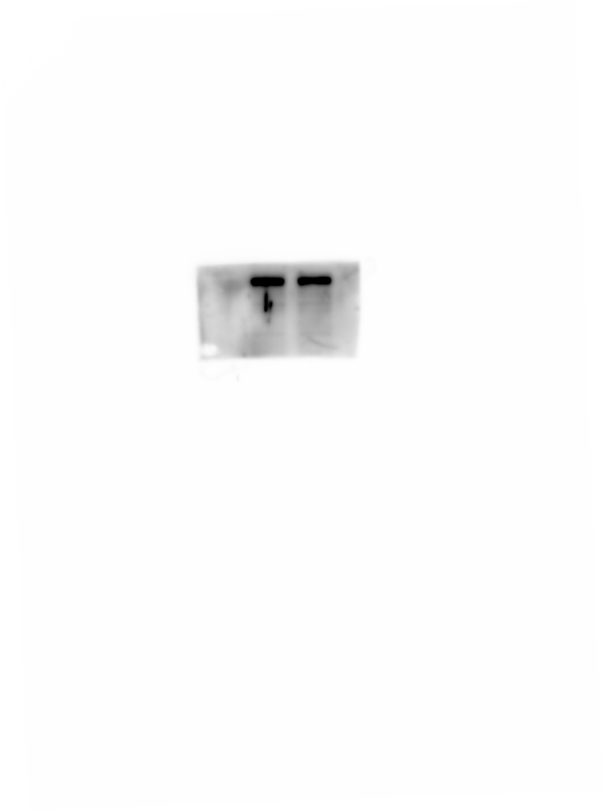

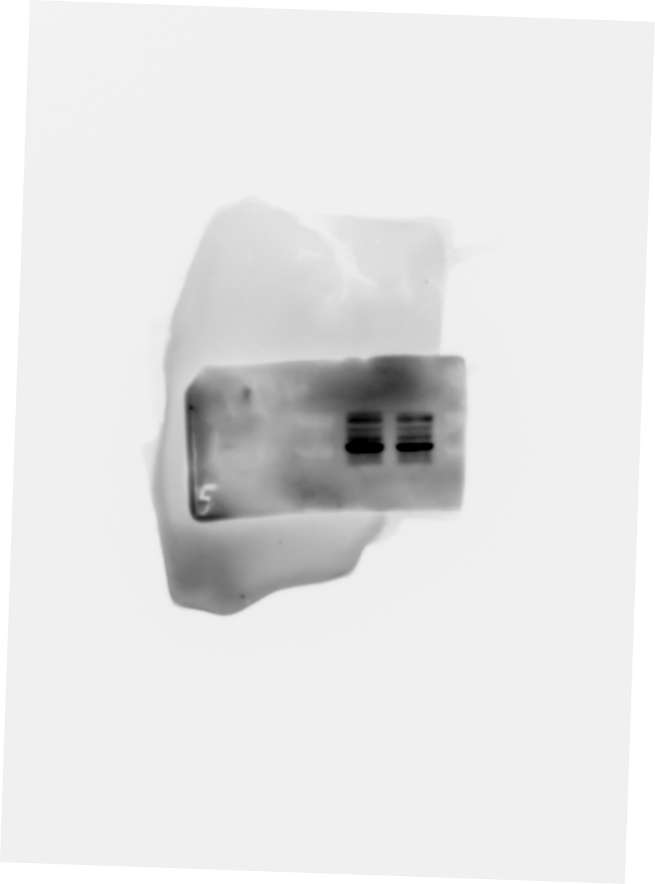

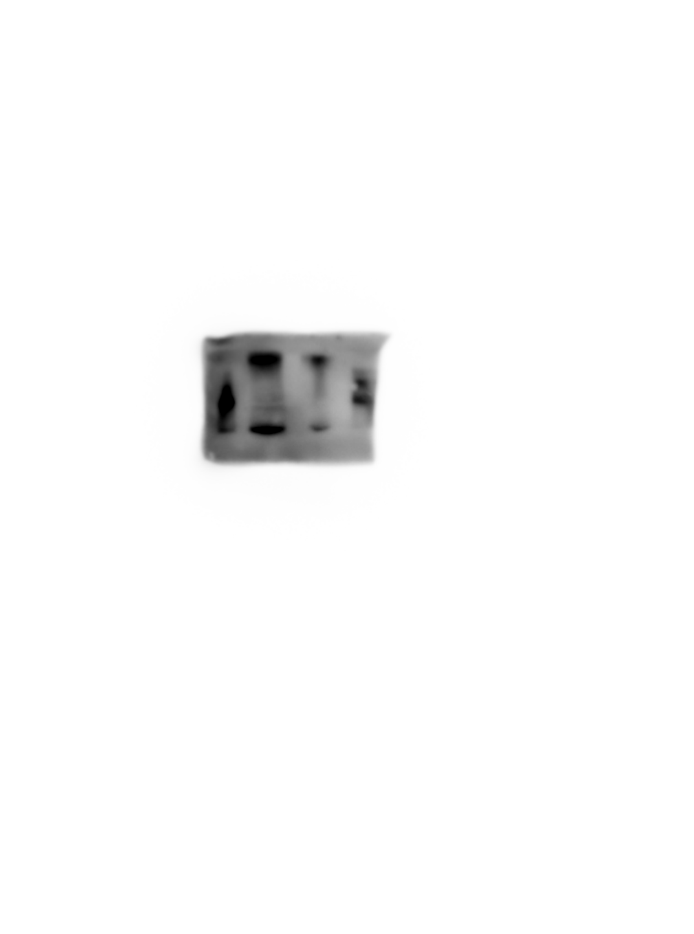


**HEK293**

**S829A**

**S829E**

**FLAG-KDM6A**

**β-ACTIN**

**SFN**

**SFN**

**FLAG**

**FLAG**

**IP**

**WCL**


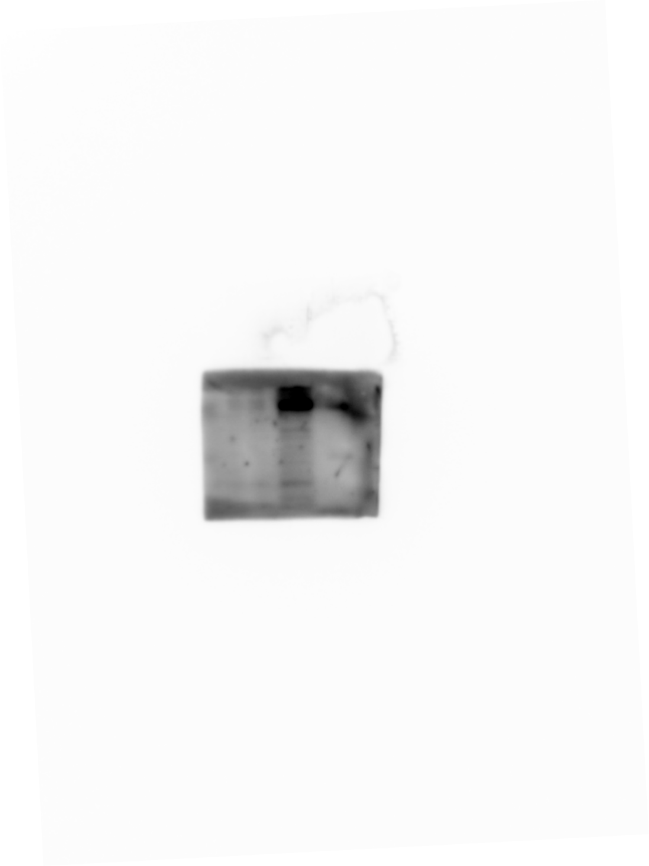


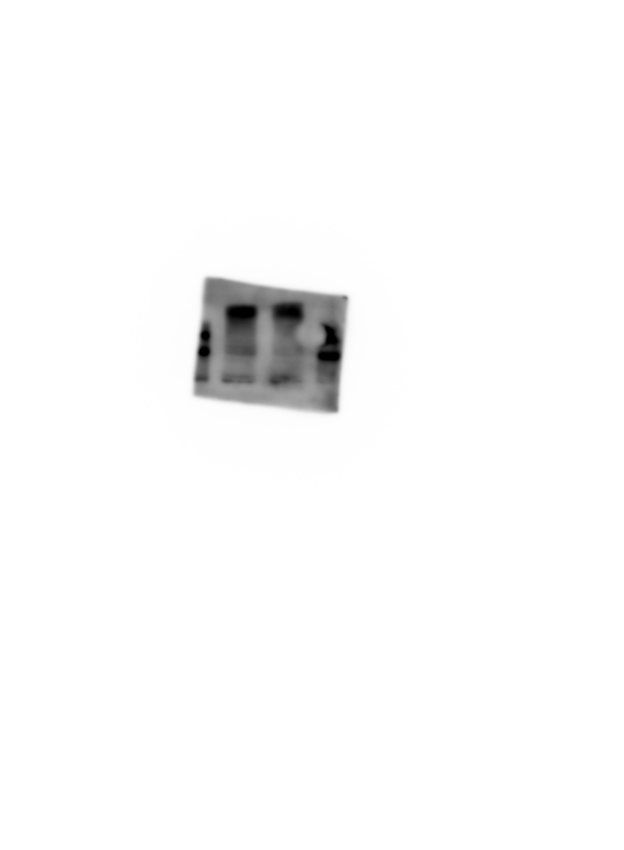


**Fig. 3F**


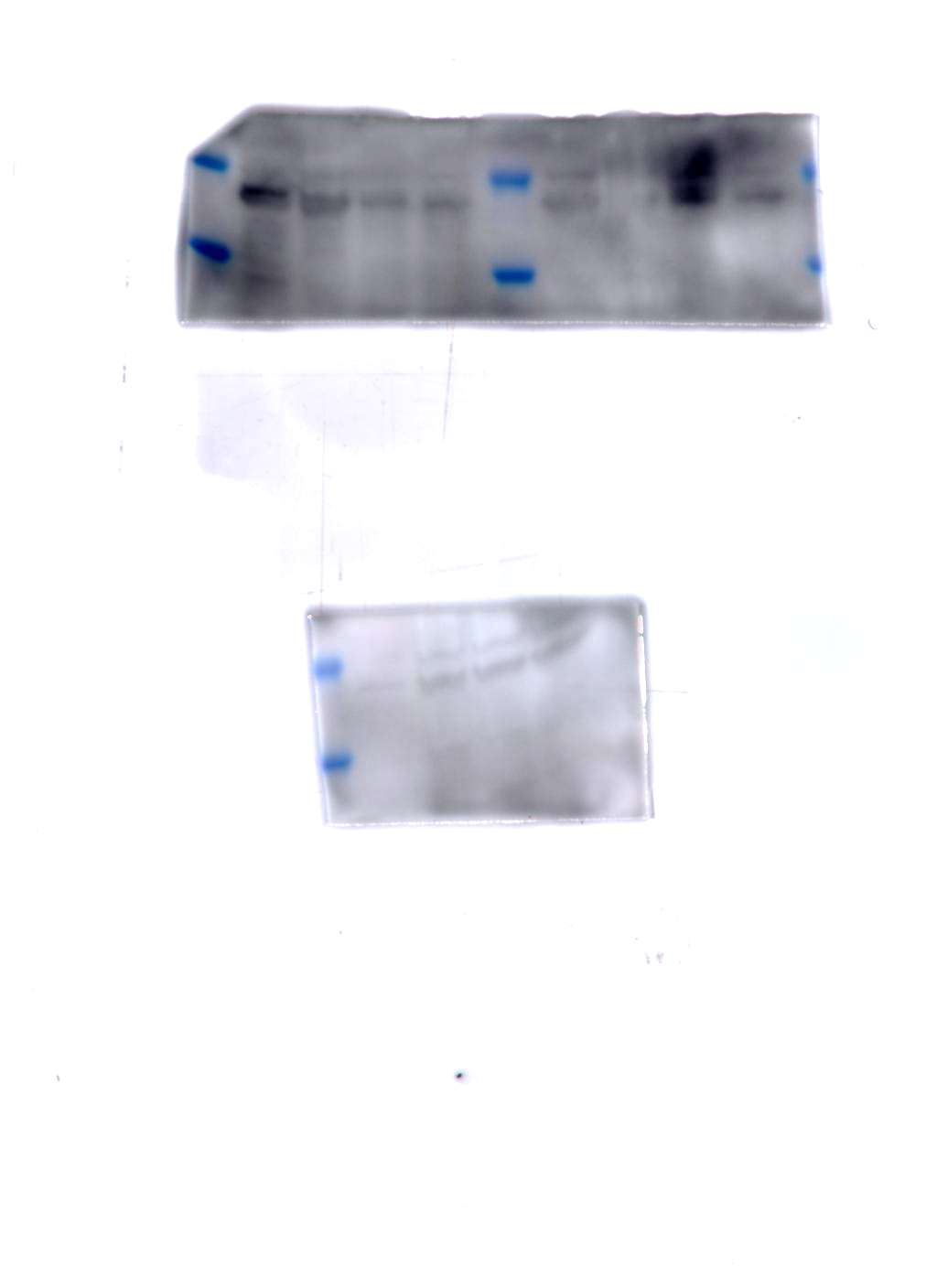


SFN

si-NC

si-SFN

si-NC

si-SFN

Cytoplasm

Nucleus


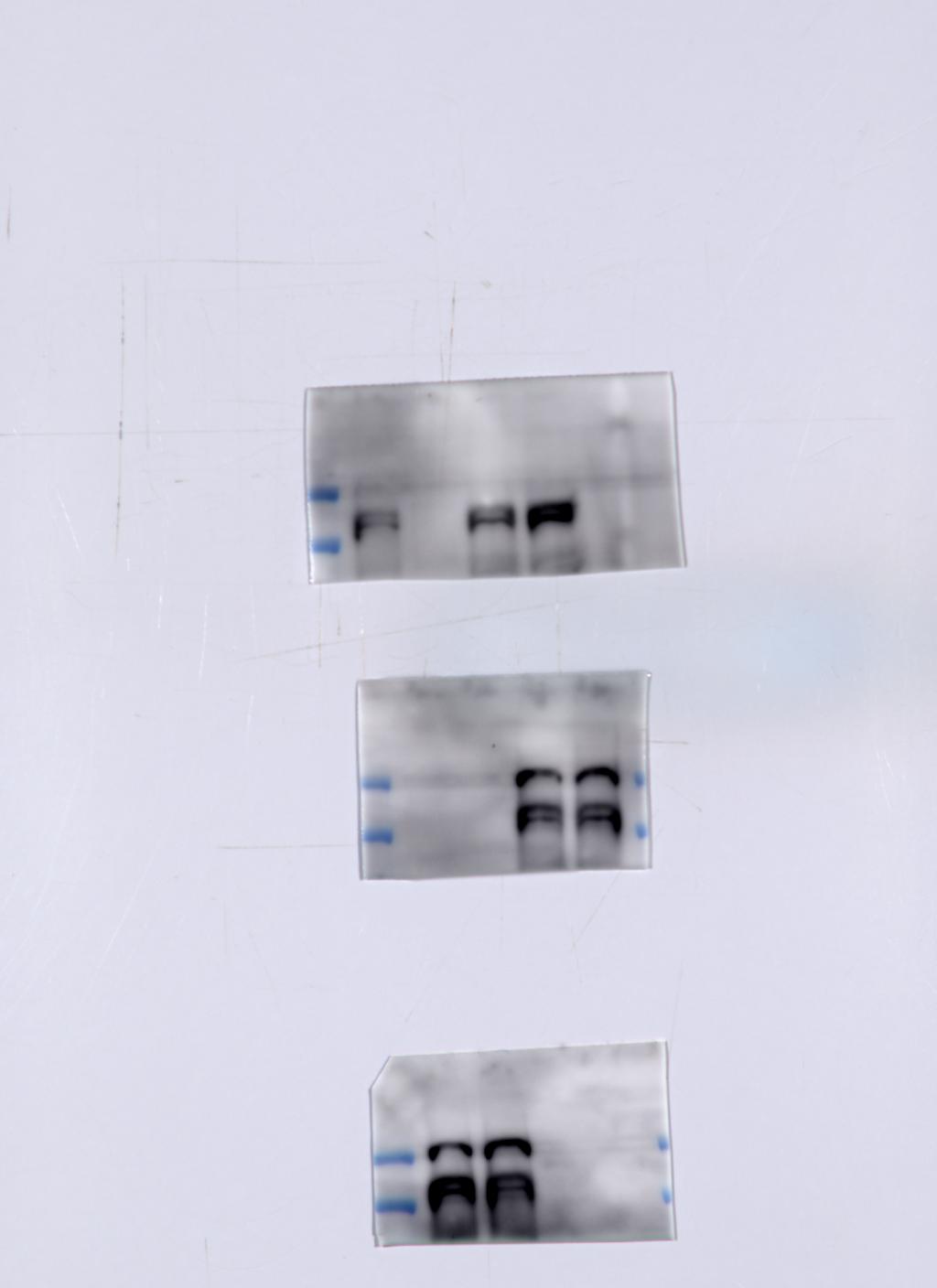


p-KDM6A


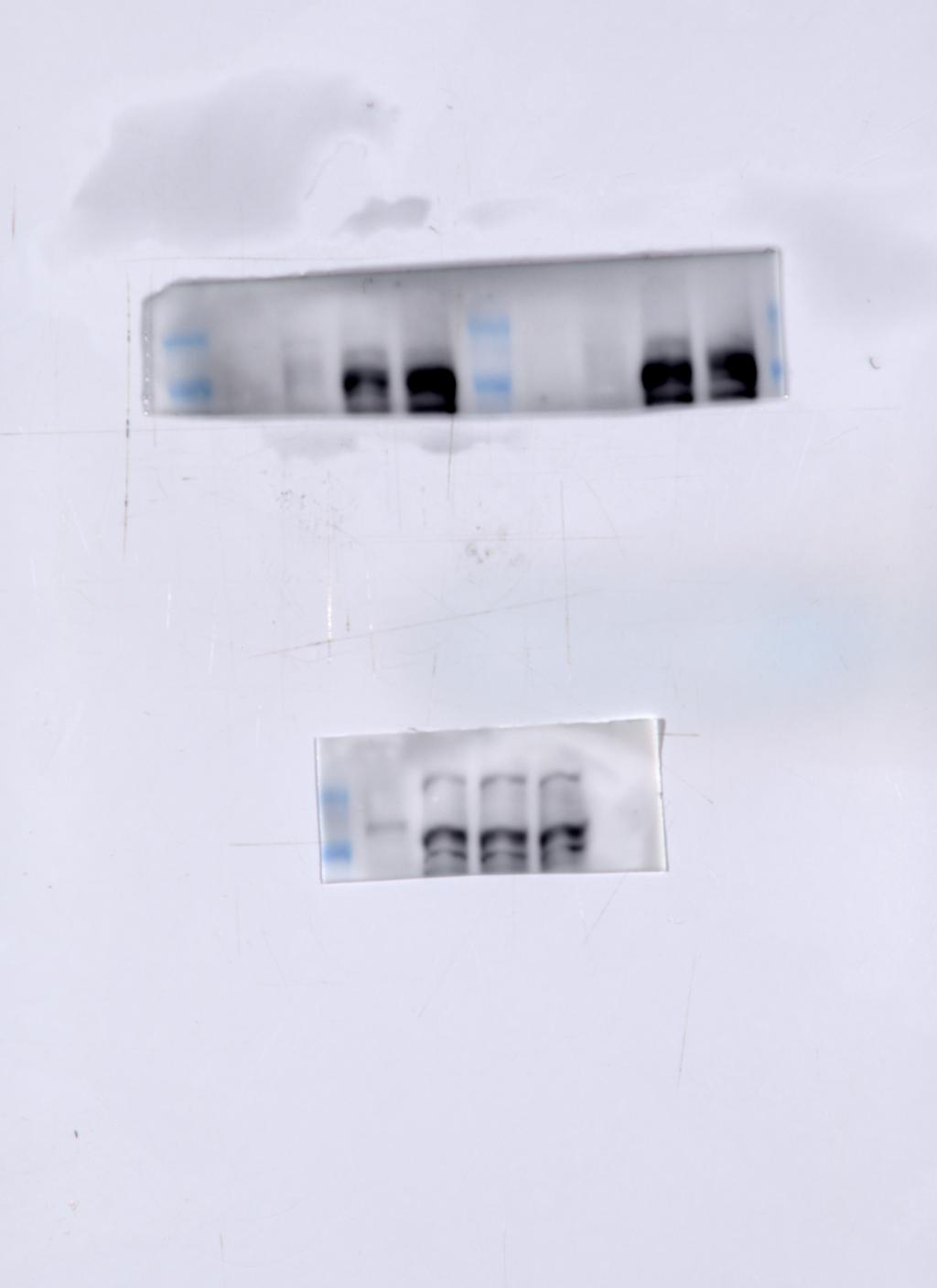


KDM6A


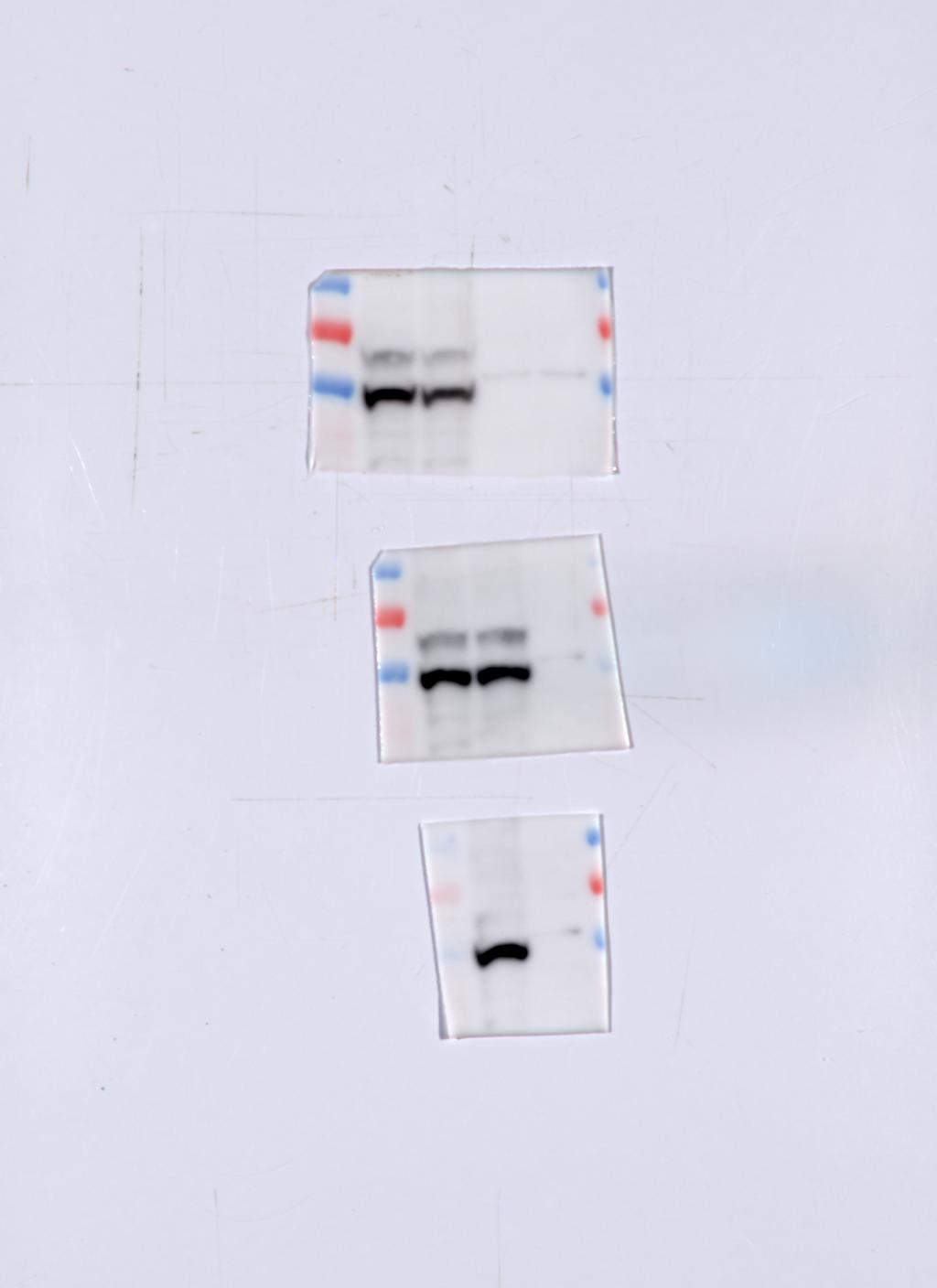


TUBLIN


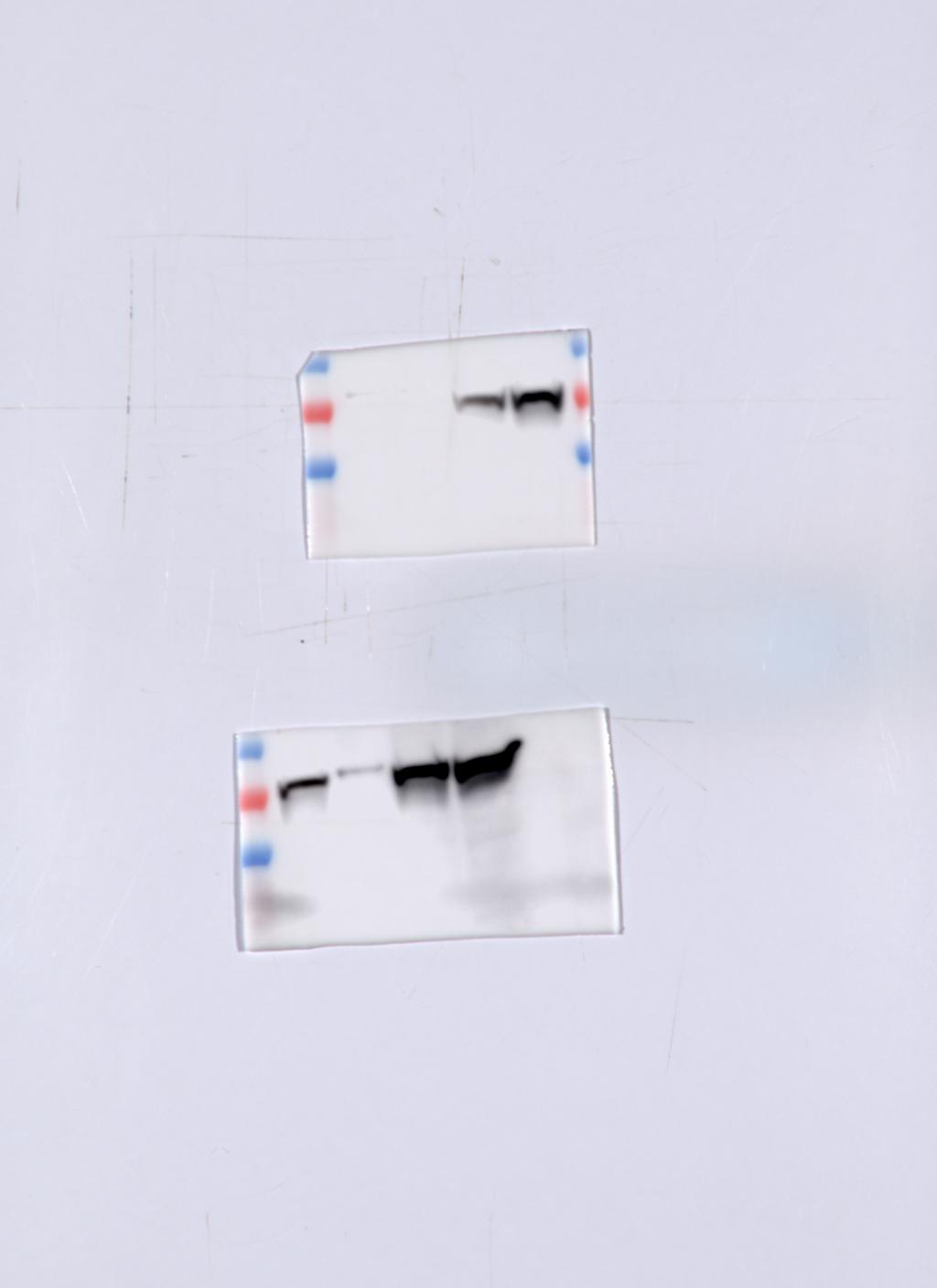


LAMIN B1

**
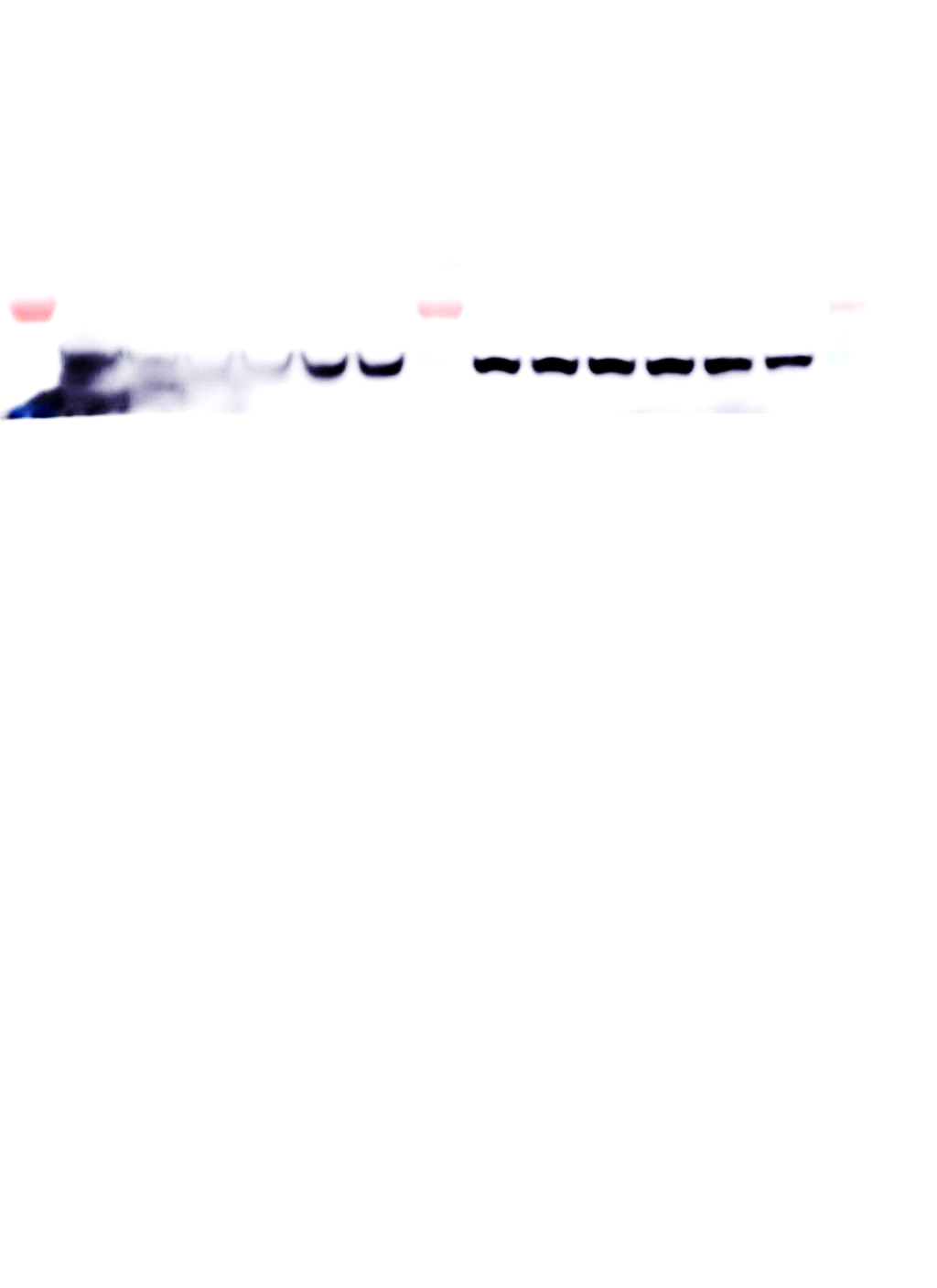

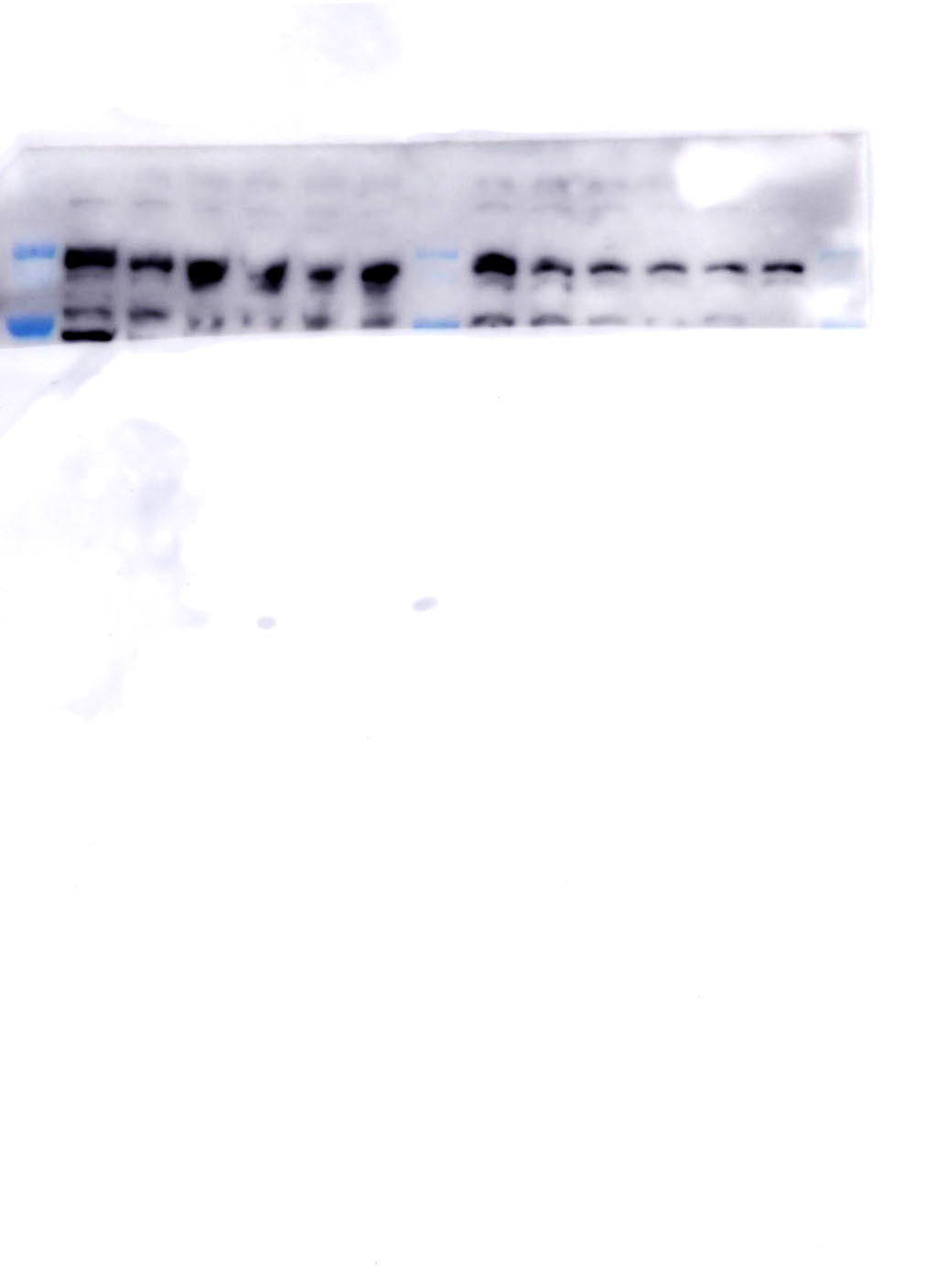

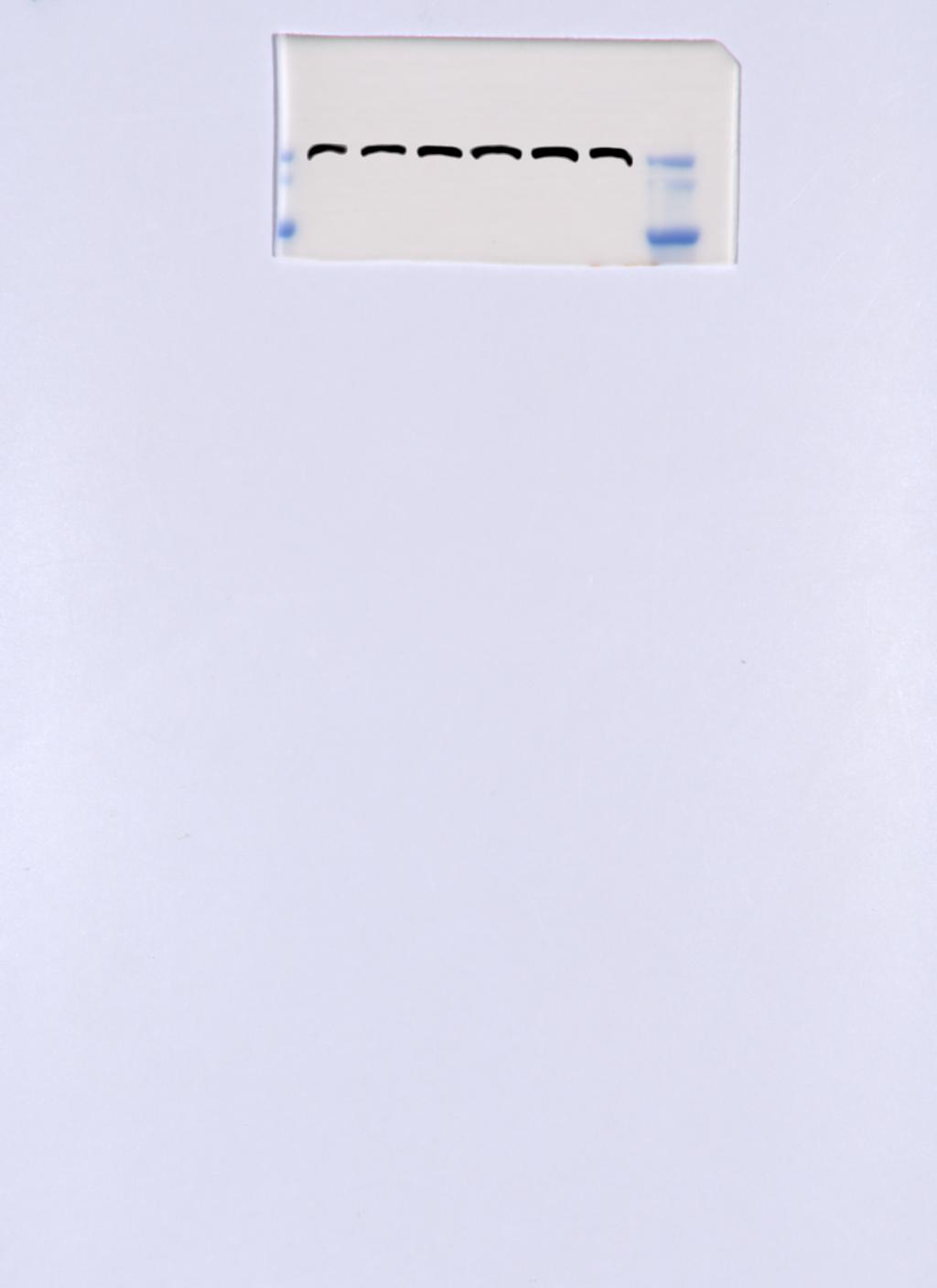
Fig. 3I**

KDM6A

p-KDM6A

Time(min)

0

5

15

30

45

60

Flavopiridol (10nM)

TUBLIN

**Fig. 3J**


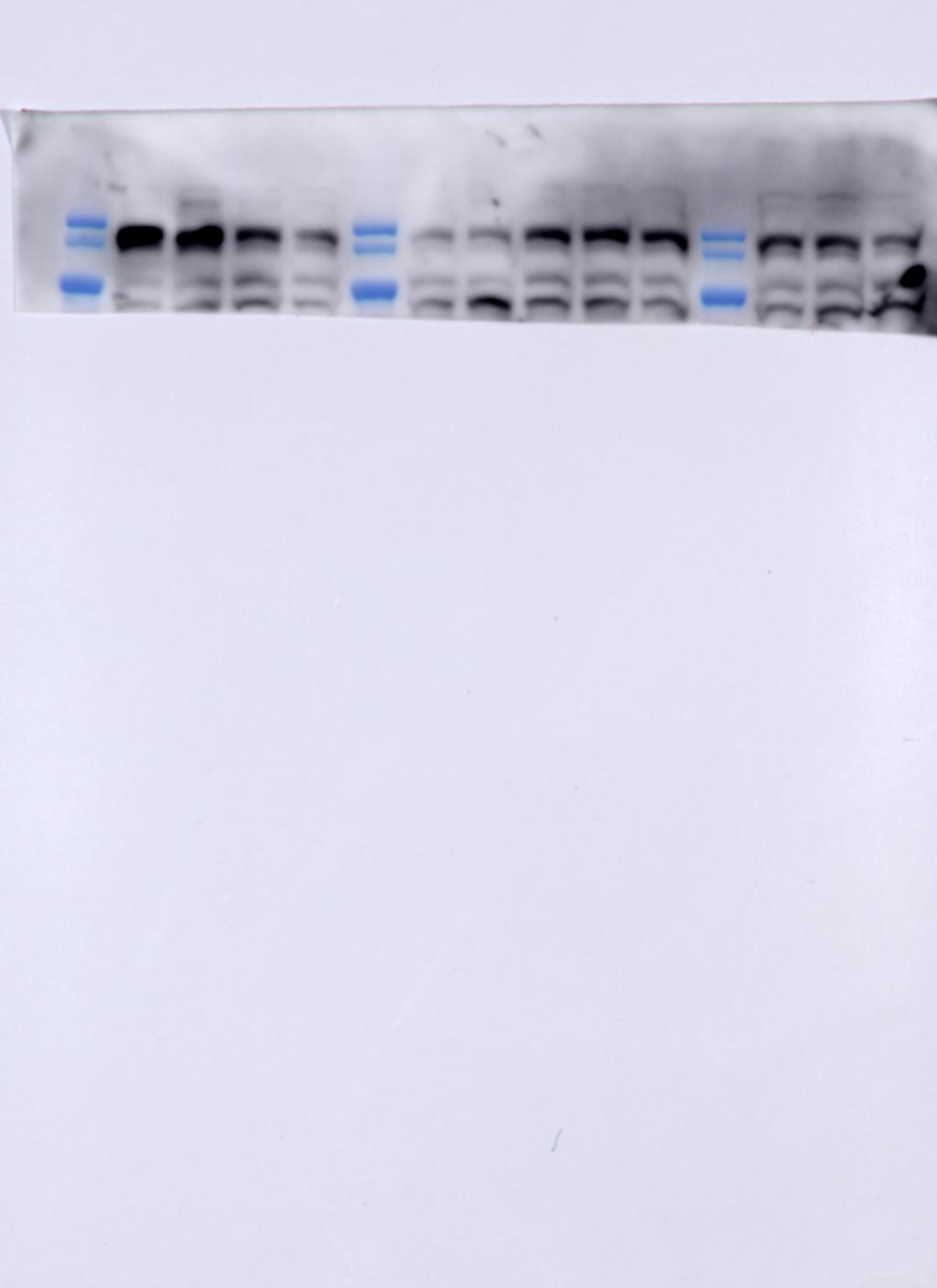

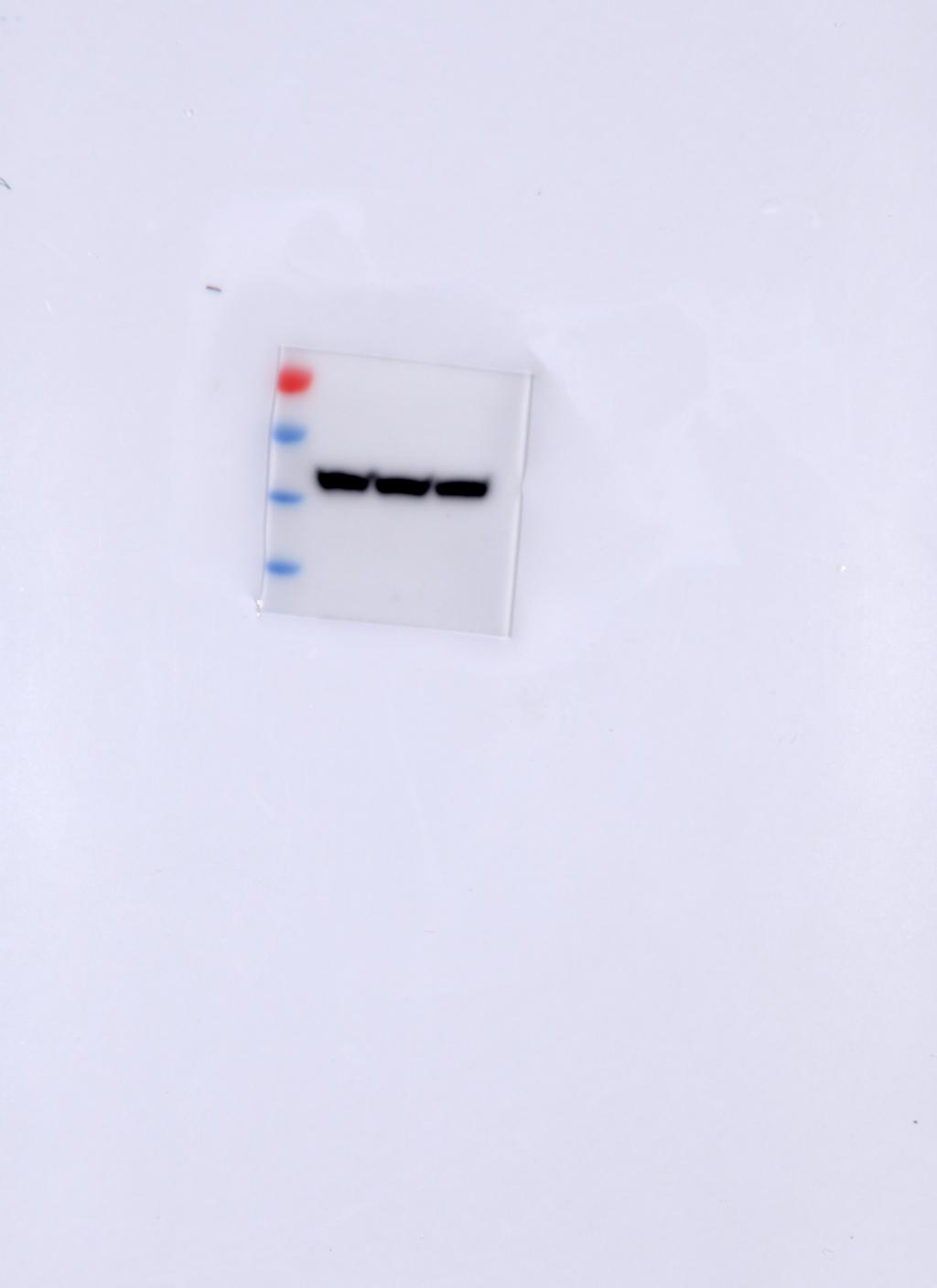

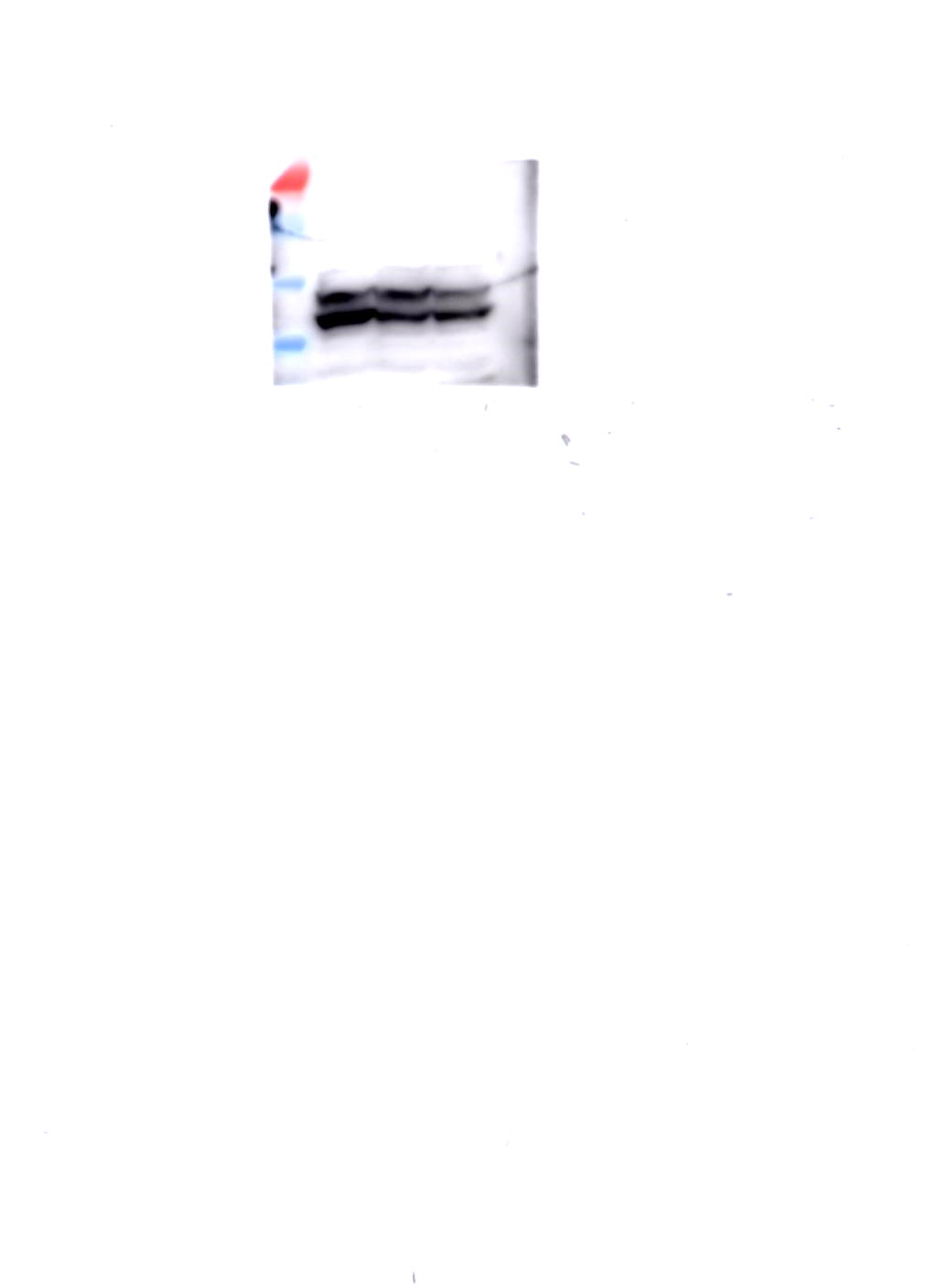

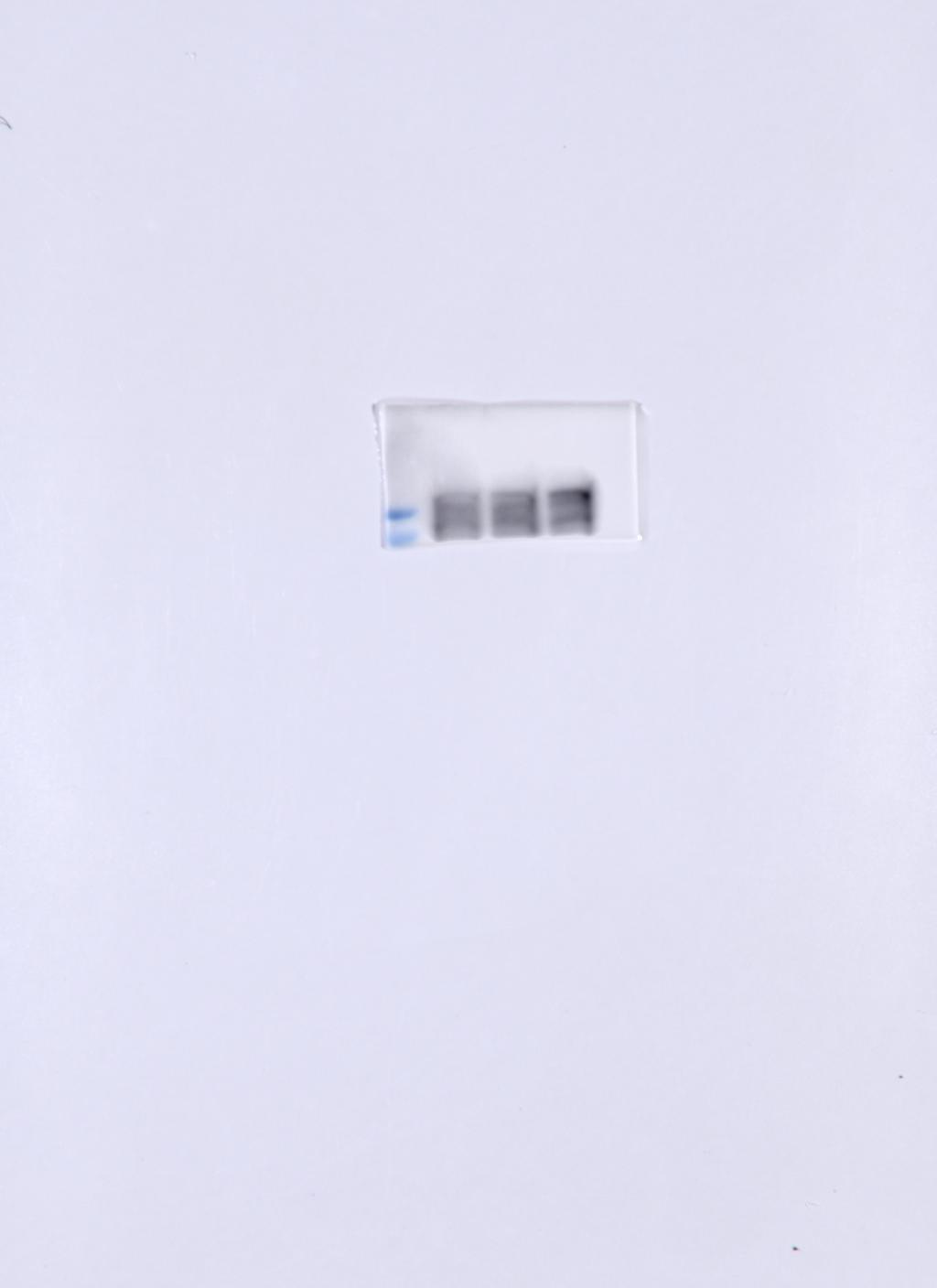


CAL27

p-KDM6A

β-ACTIN

si-NC

si-CDK1-1

si-CDK1-2

KDM6A

CDK1

**Fig. 3K**


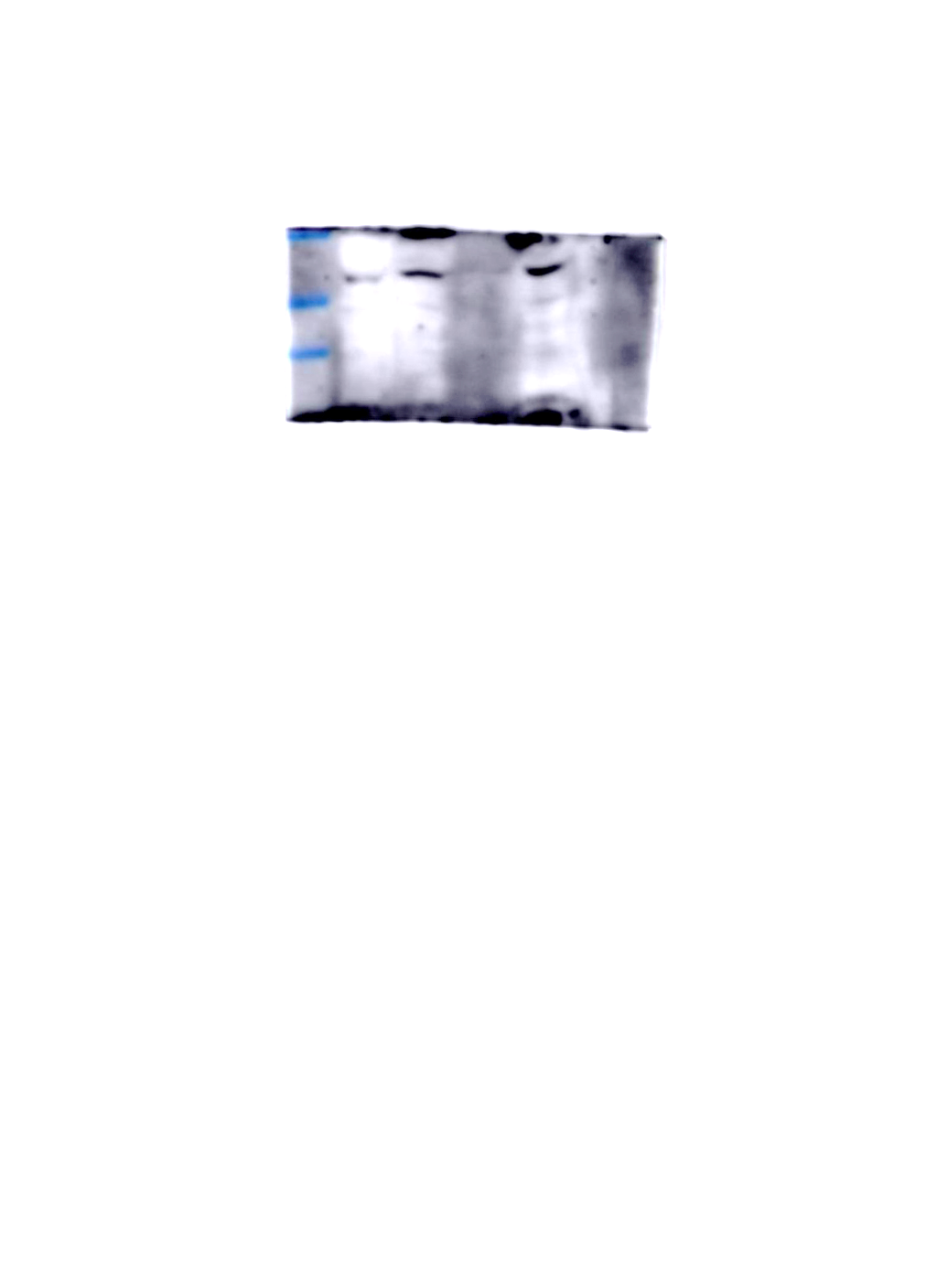

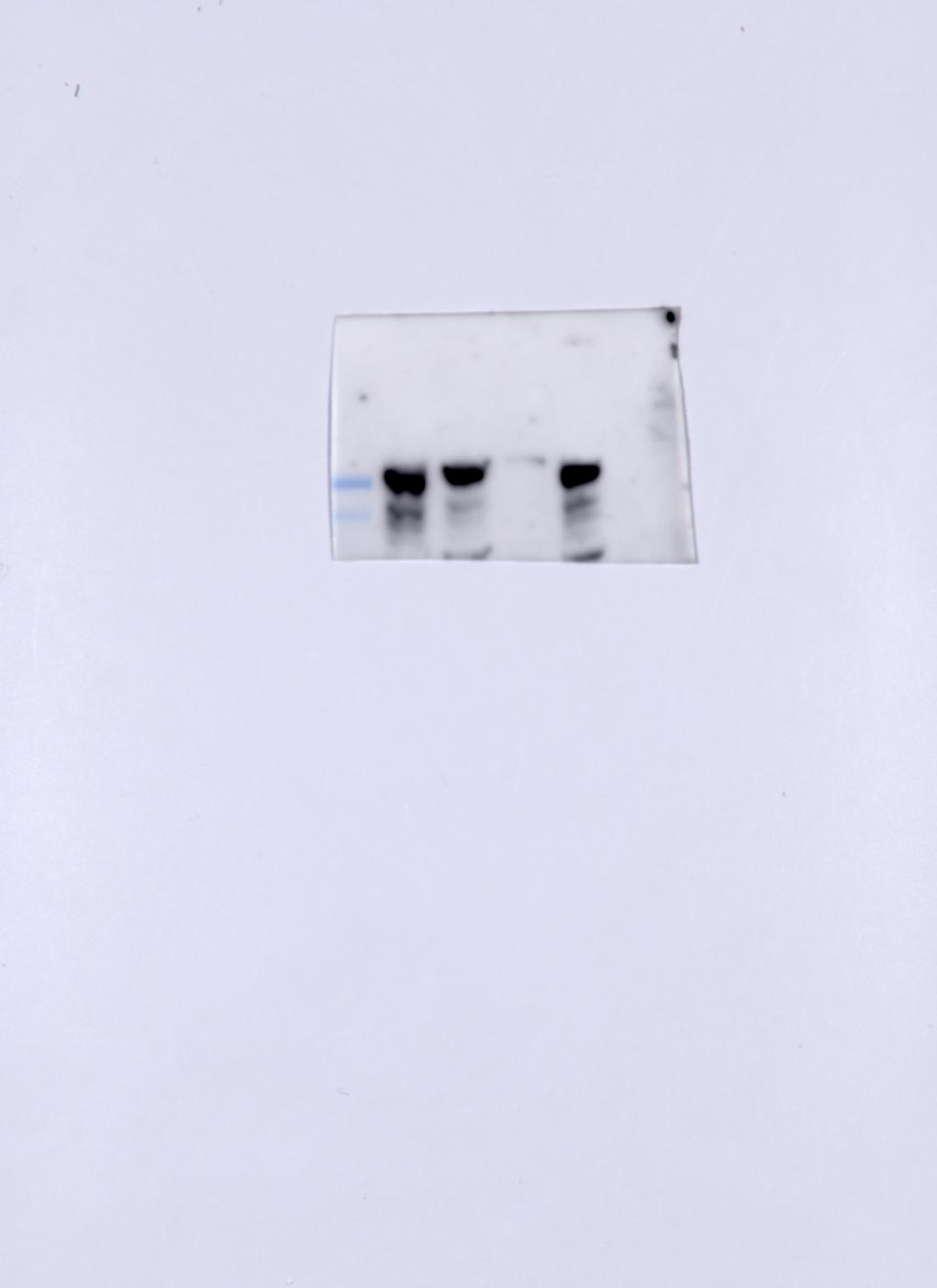

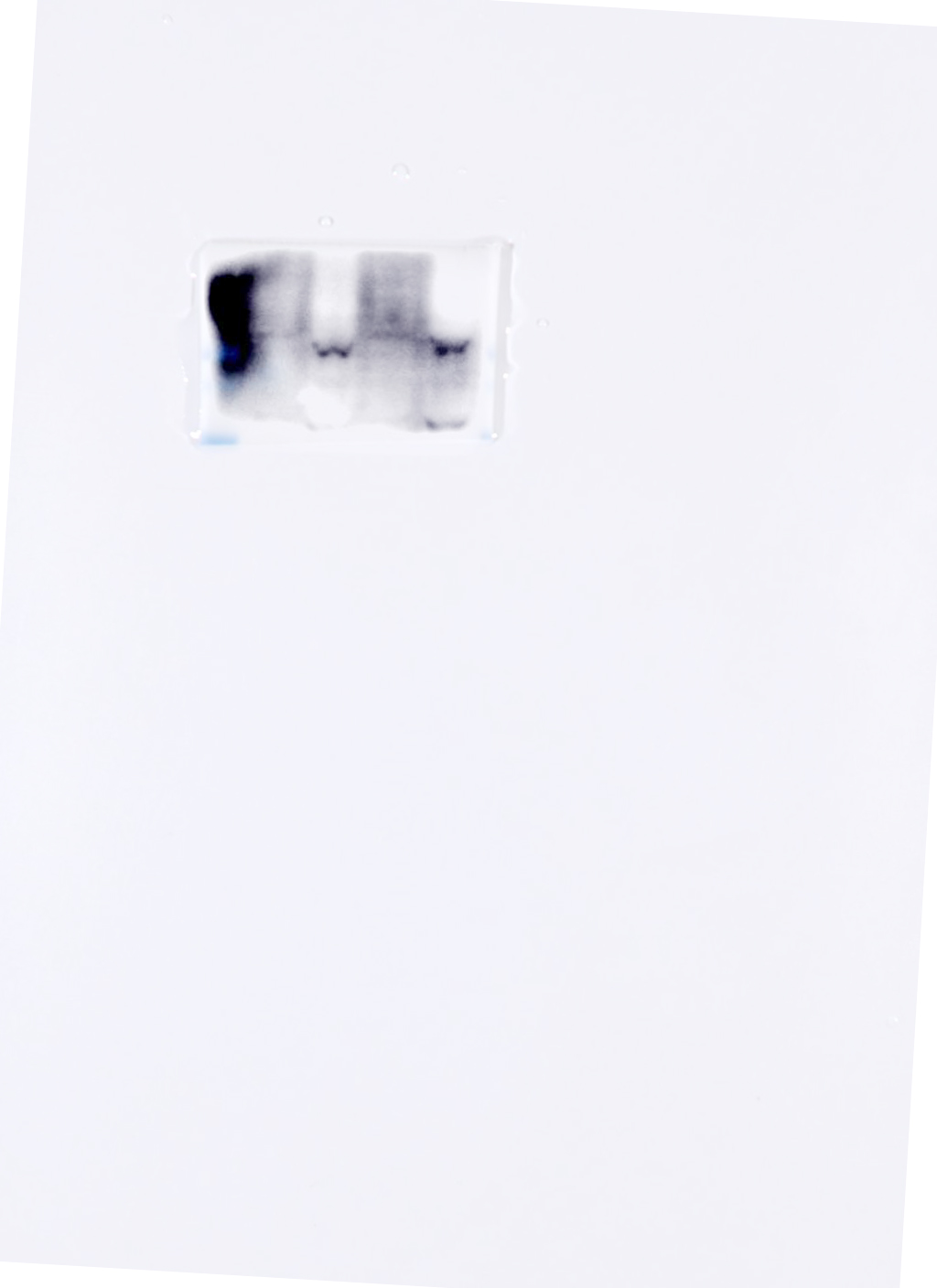

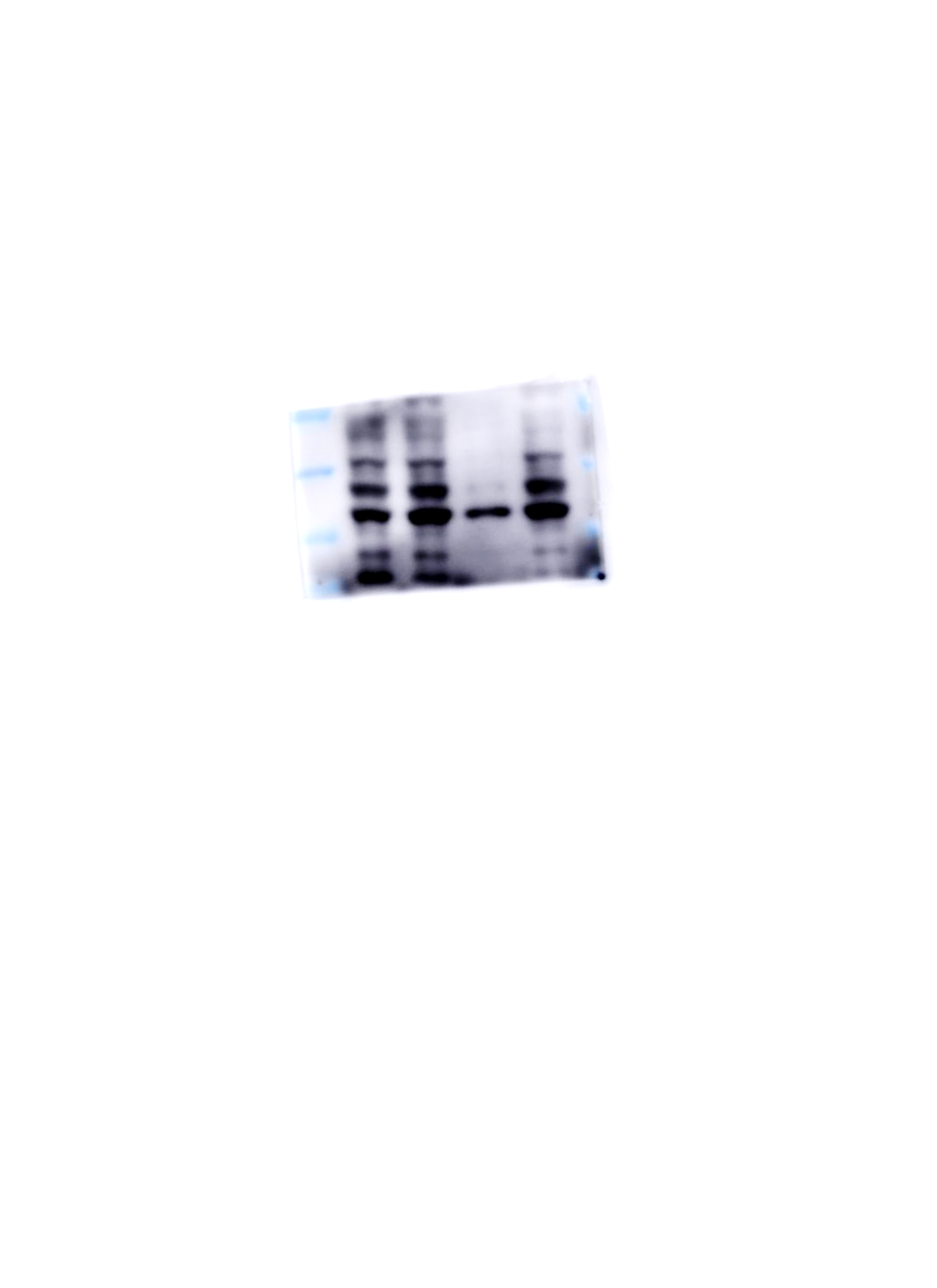


IP:HA-CDK1

Input

KDM6A

HA-CDK1

Ctrl

CDK1

Ctrl

CDK1

FLAG-KDM6A

CDK1

IP:FLAG-KDM6A

Input

Ctrl

KDM6A

Ctrl

KDM6A

*

**Fig. 3L**


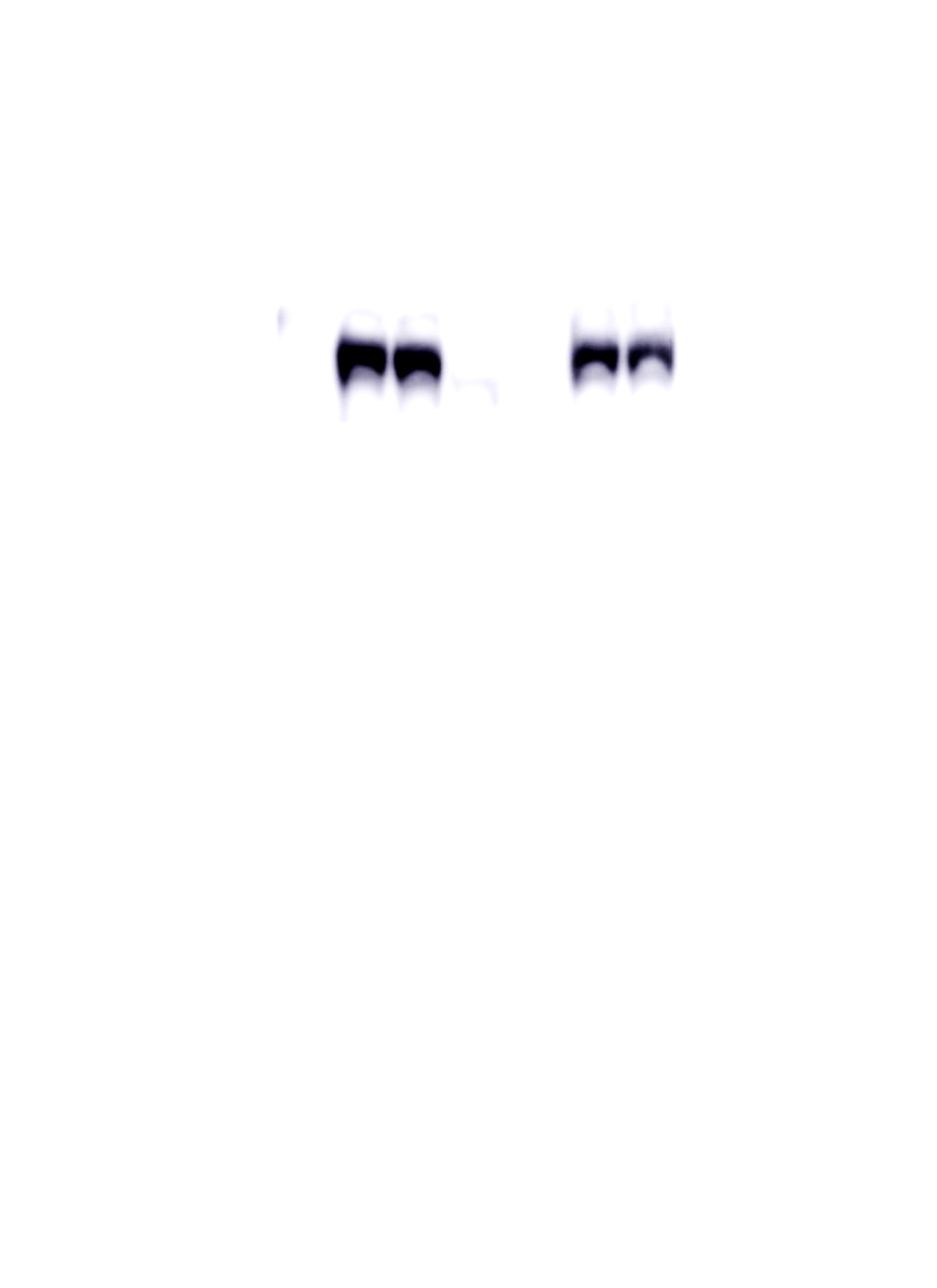

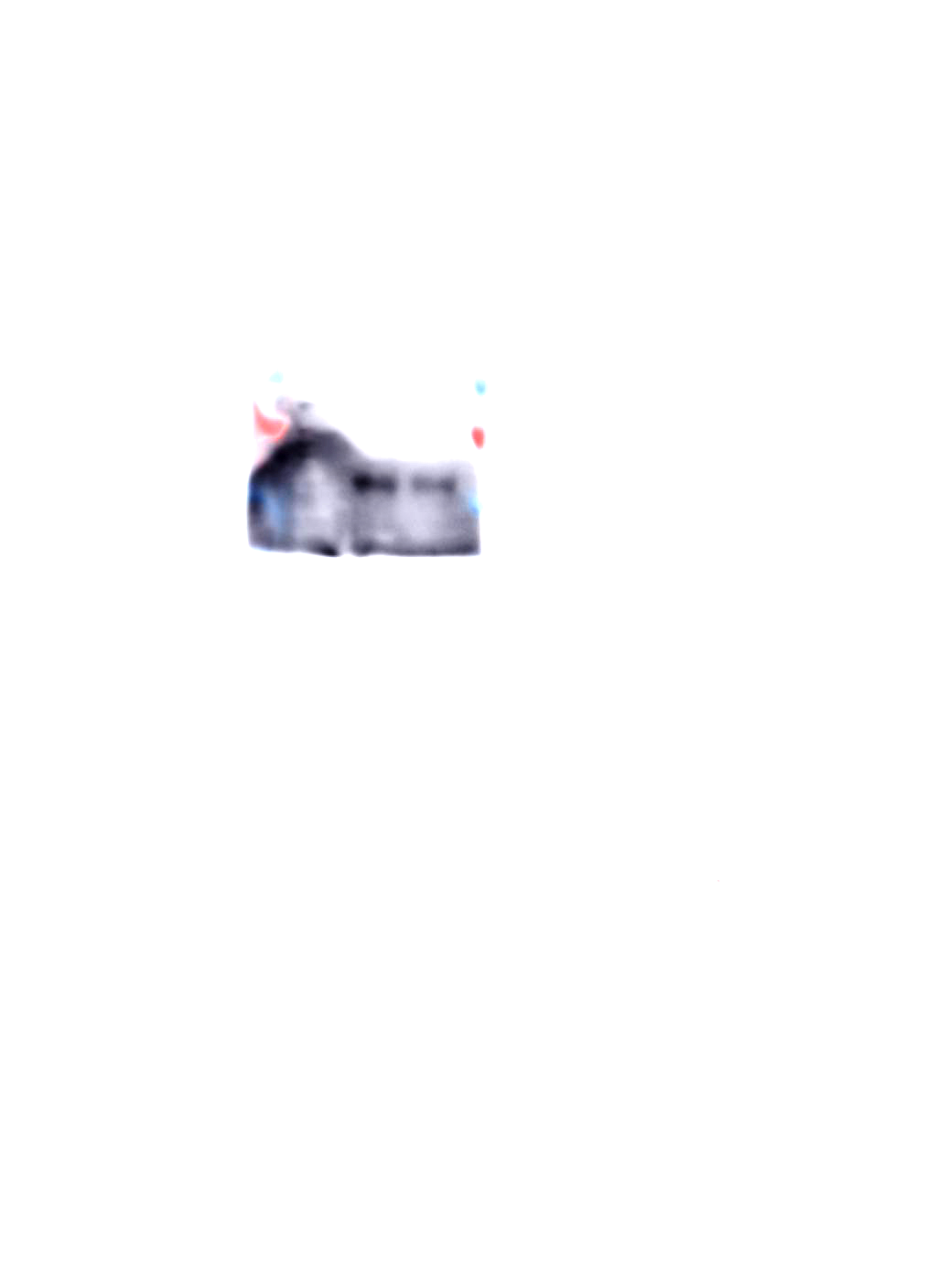

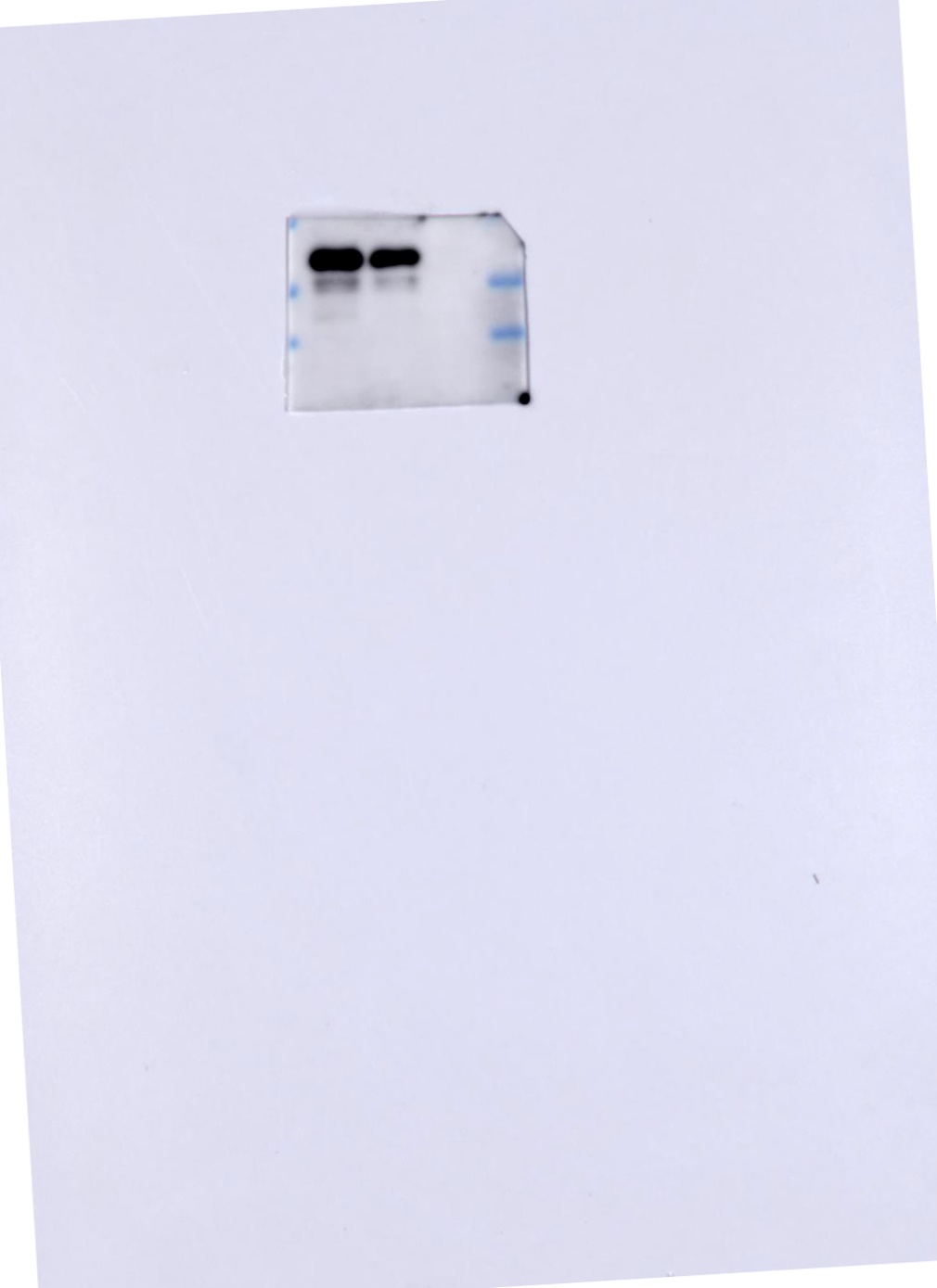

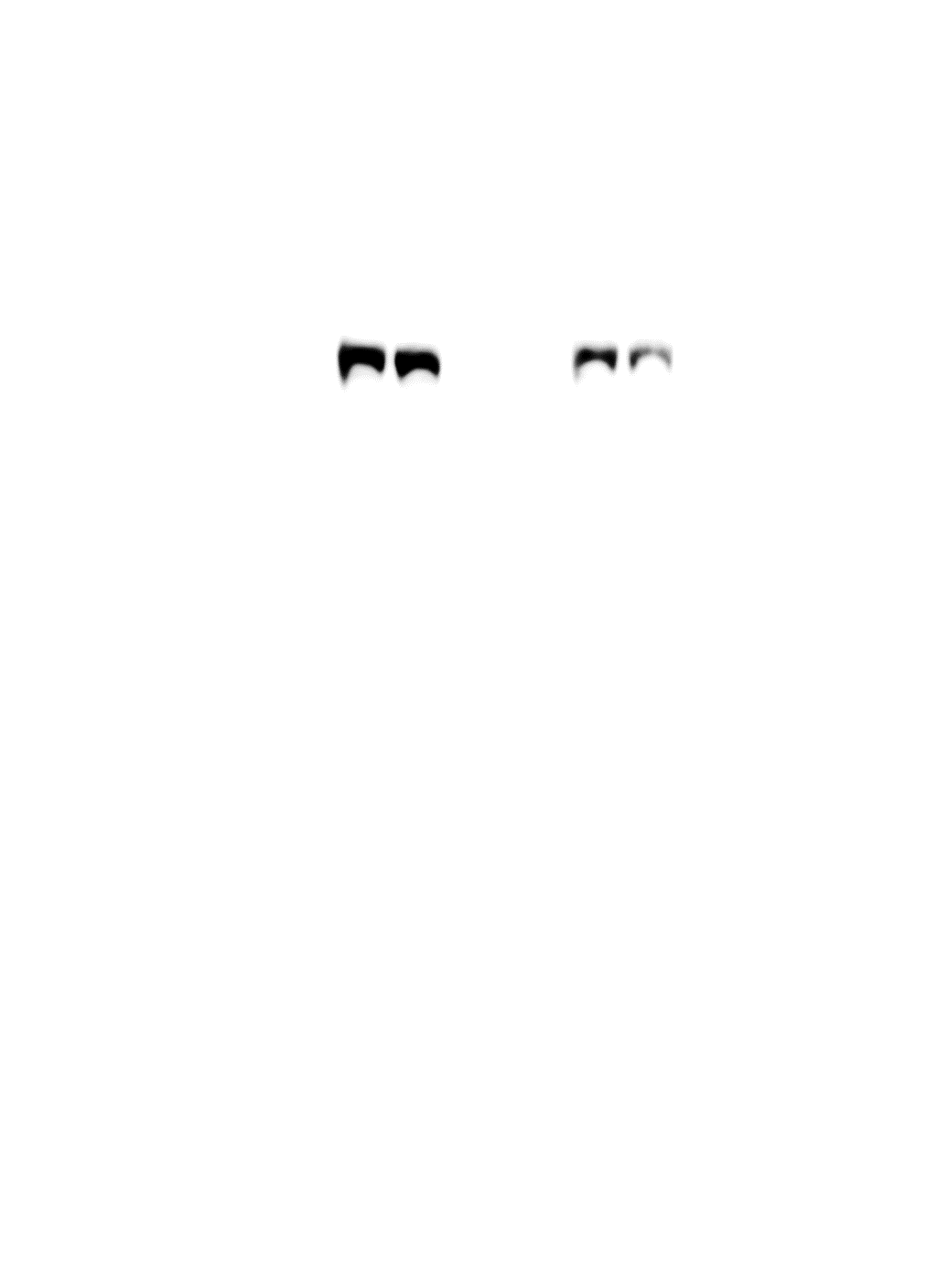

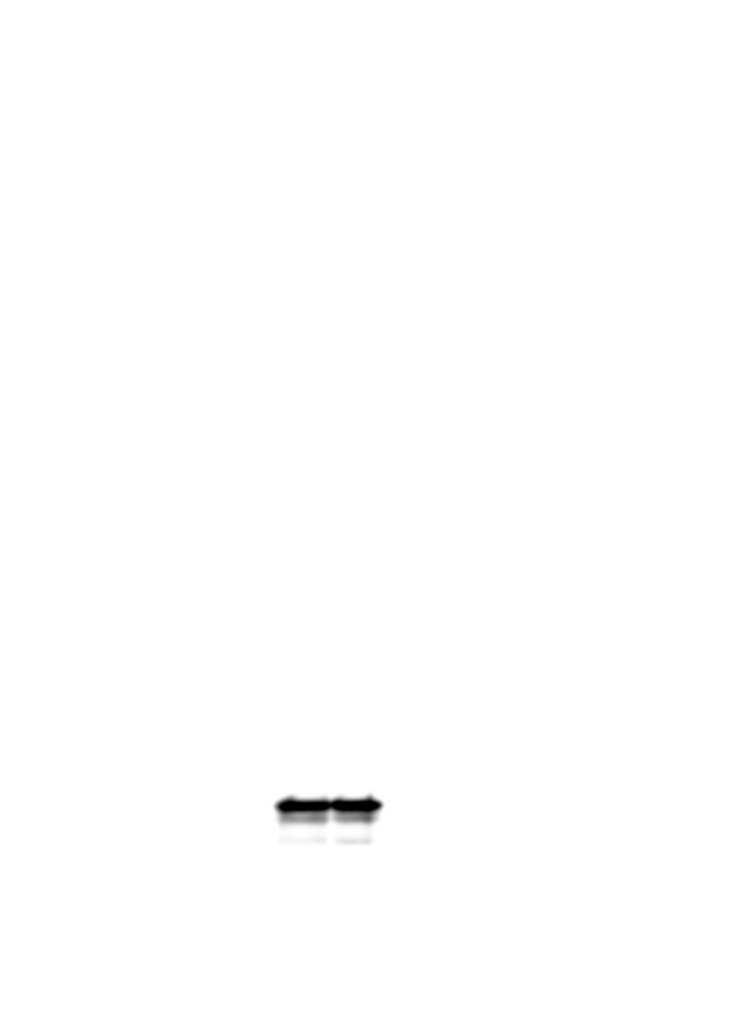


IgG

IP:CDK1

Input

IP:CDK1

CDK1

IgG

KDM6A

KDM6A

IgG

CDK1

IP:KDM6A

Input

IP:KDM6A

IgG


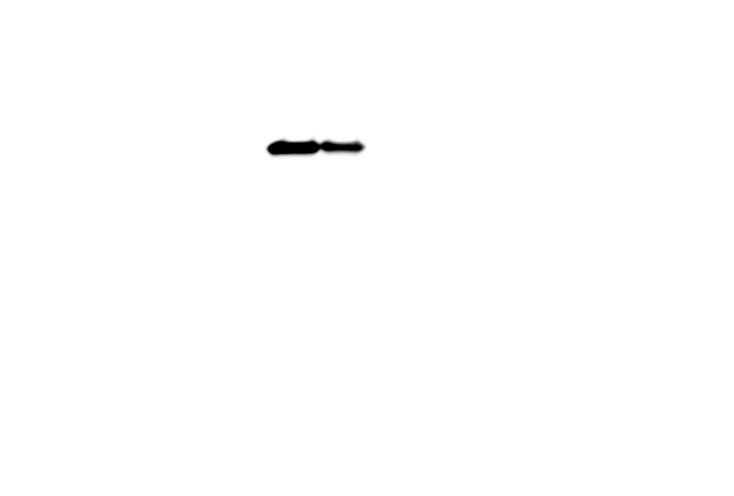


**Fig. 4A**


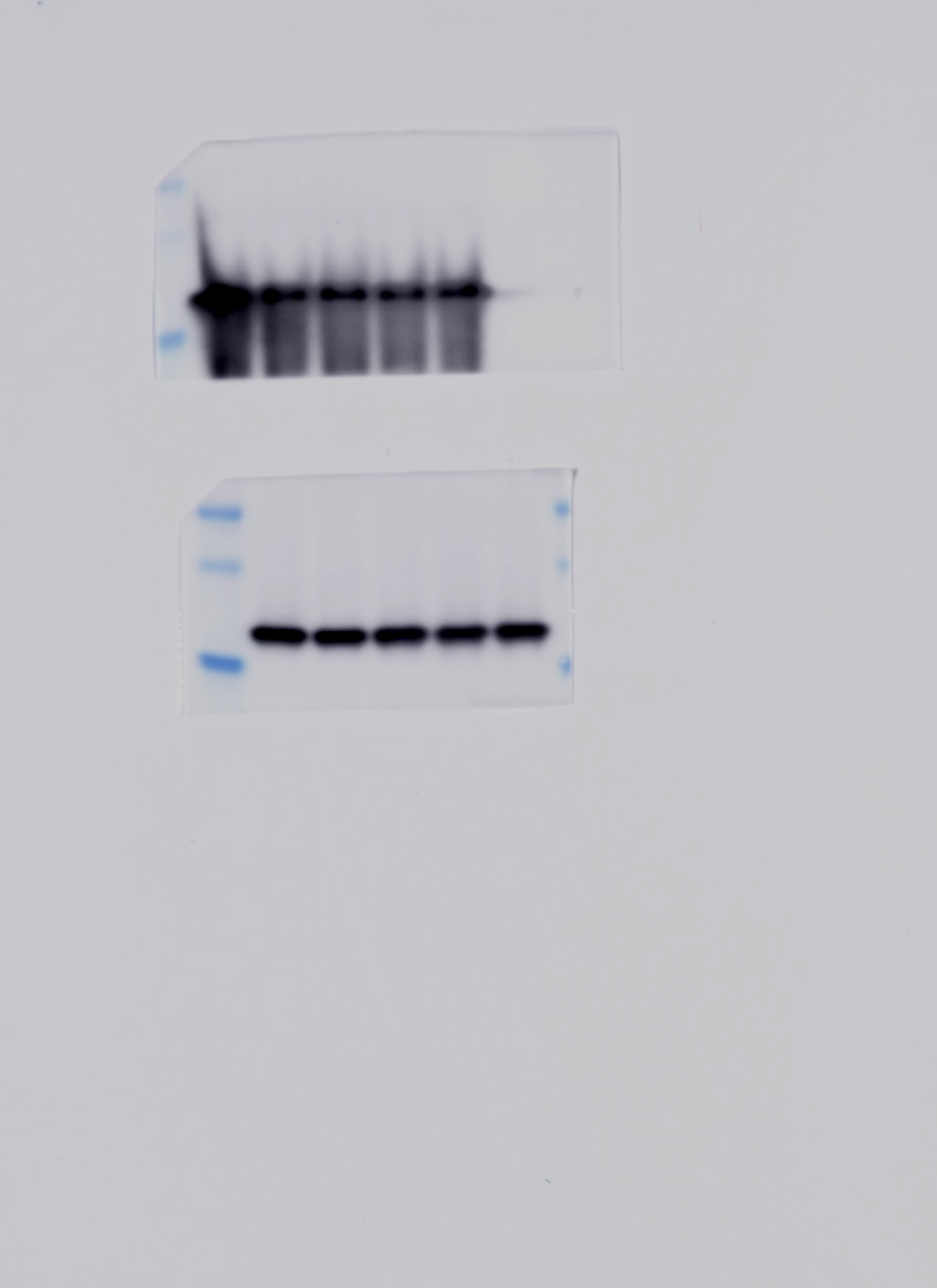

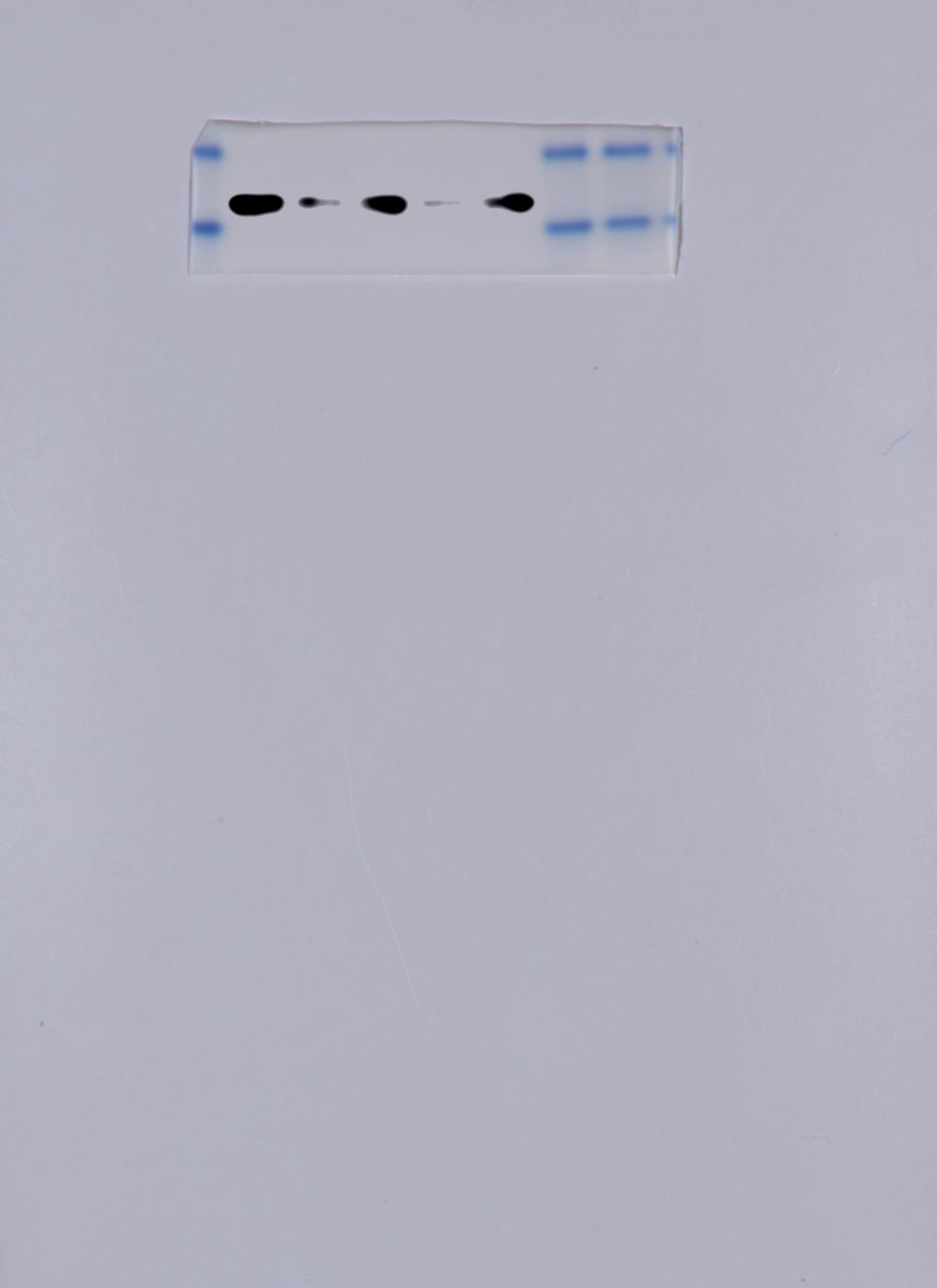


**H3**

**H3K27Me3**

**KDM6A^S829A^**

**KDM6A^S829D^**

**KDM6A^WT^**

**Vector**

**Supplementary Fig. 2A**


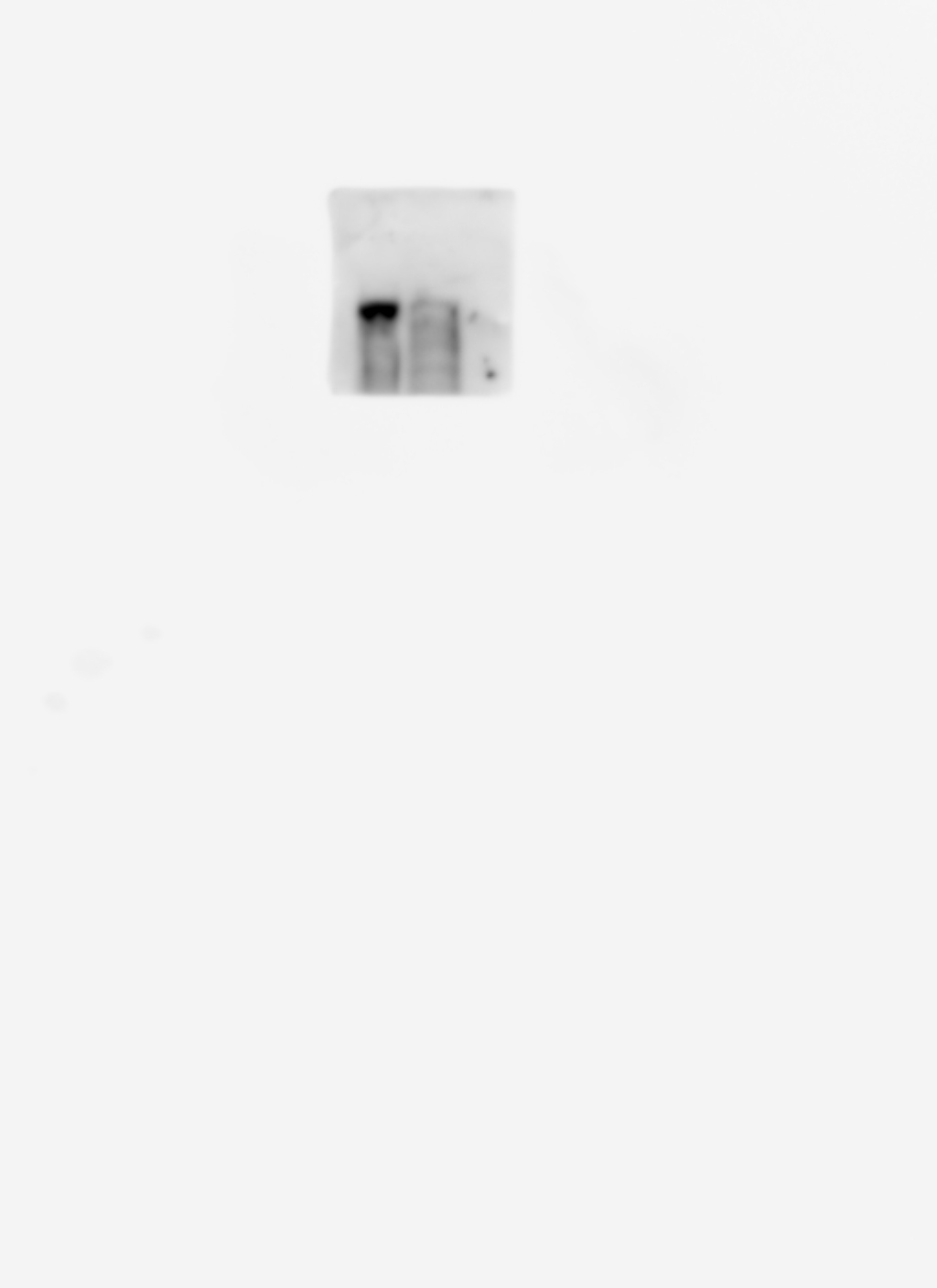


Si-KDM6A


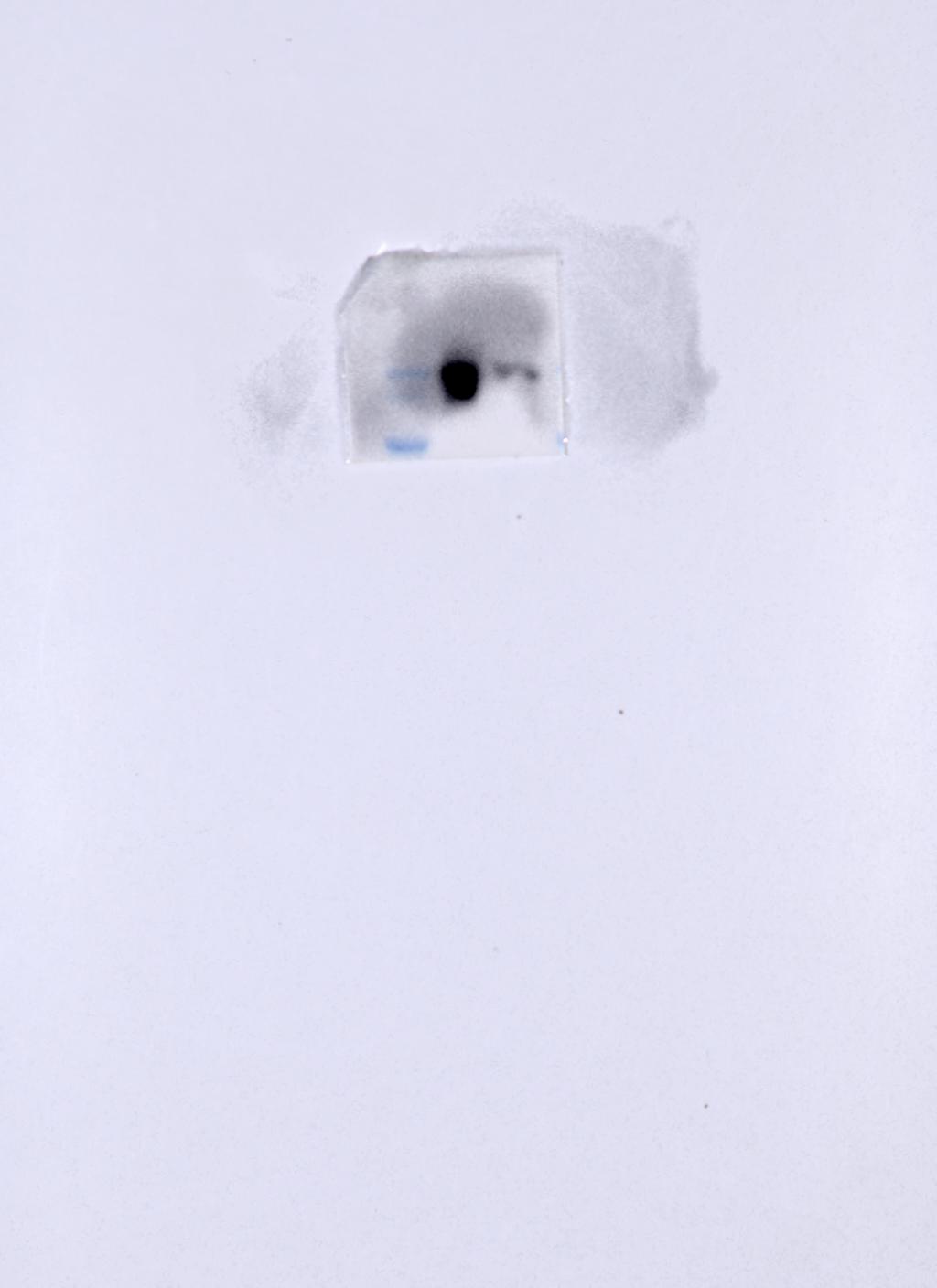

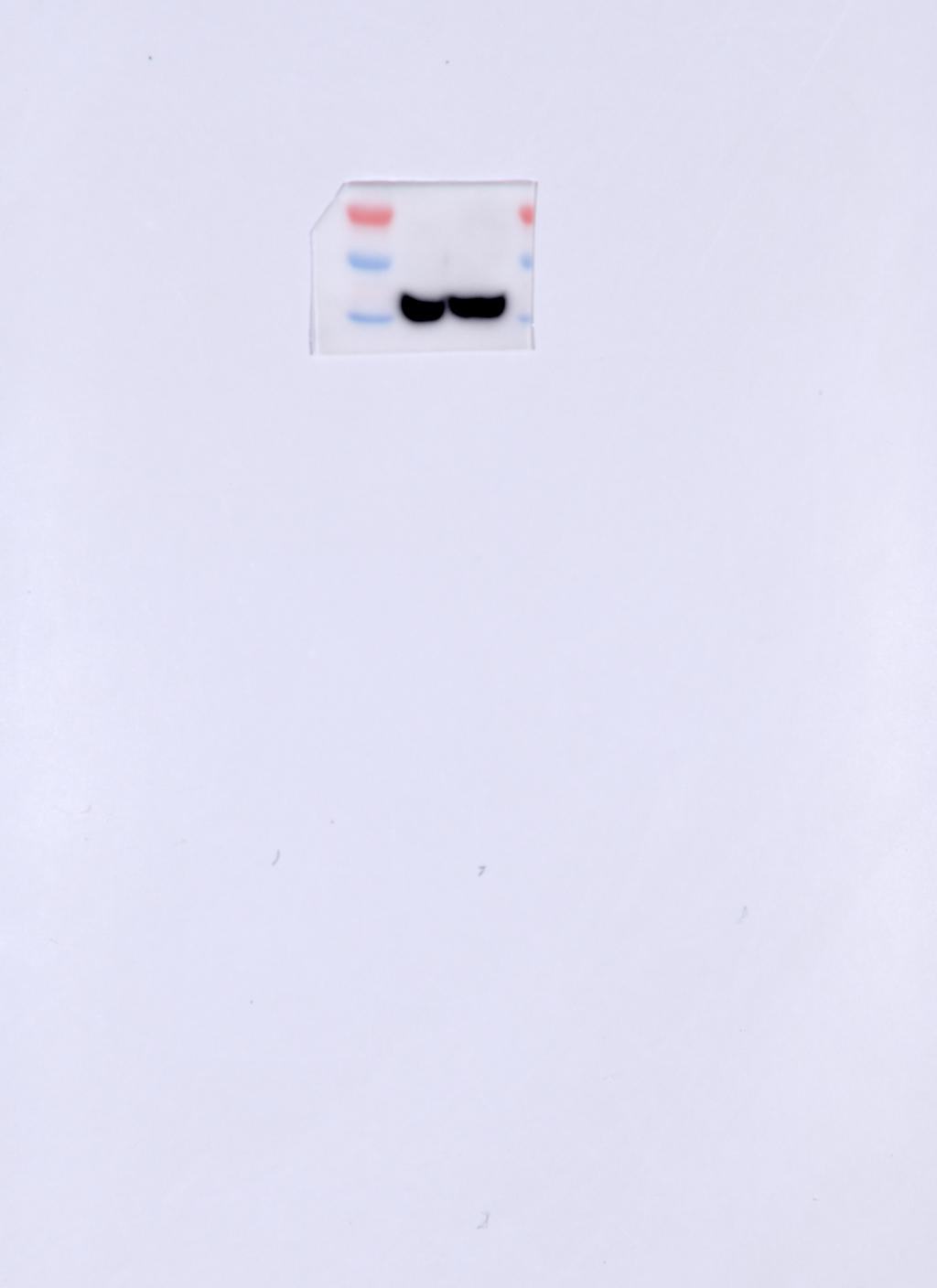

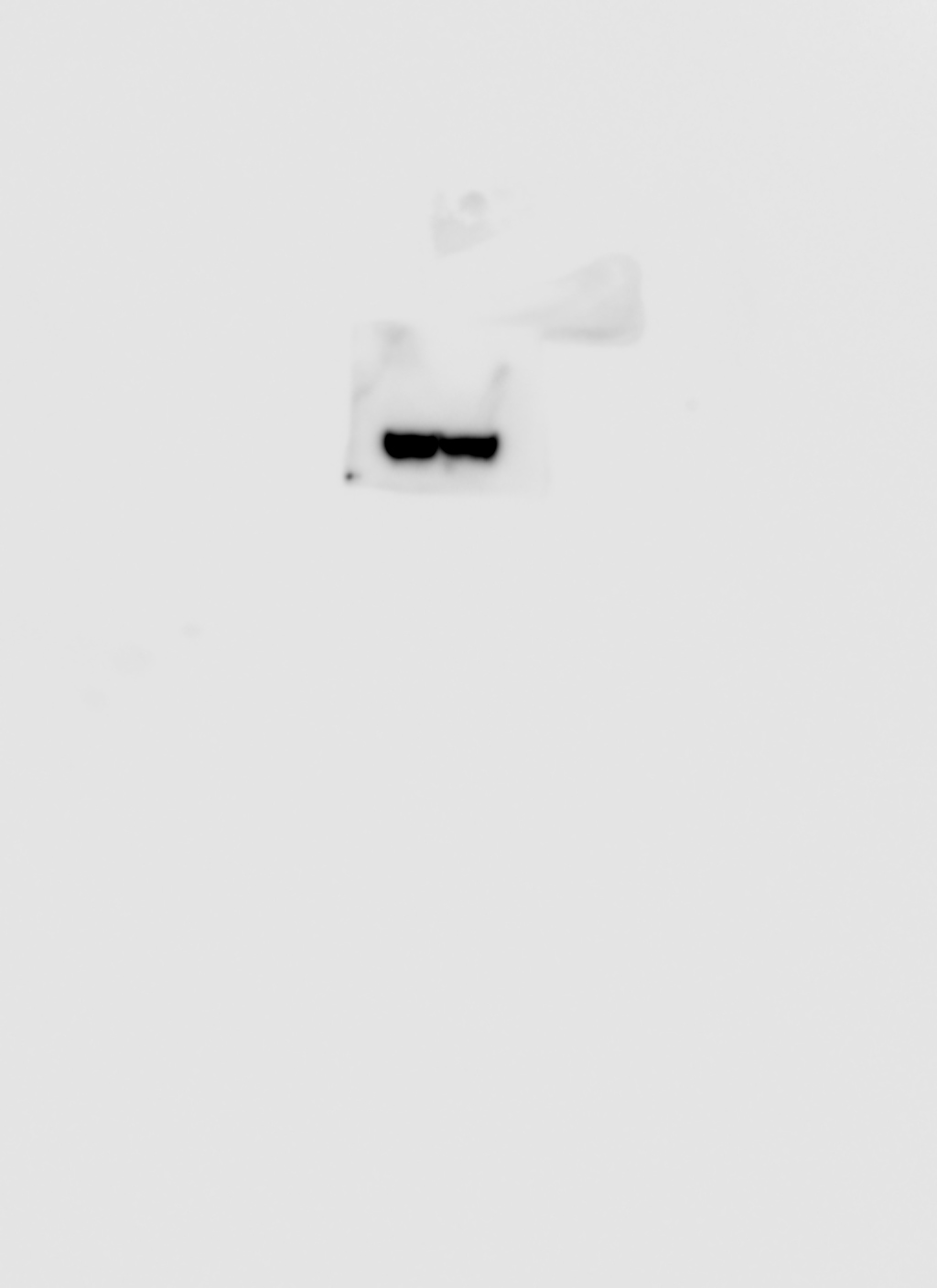


NC

Si-KDM6A

β-ACTIN

KDM6A

β-ACTIN

NC

KDM6A

**Supplementary Fig. 4C**


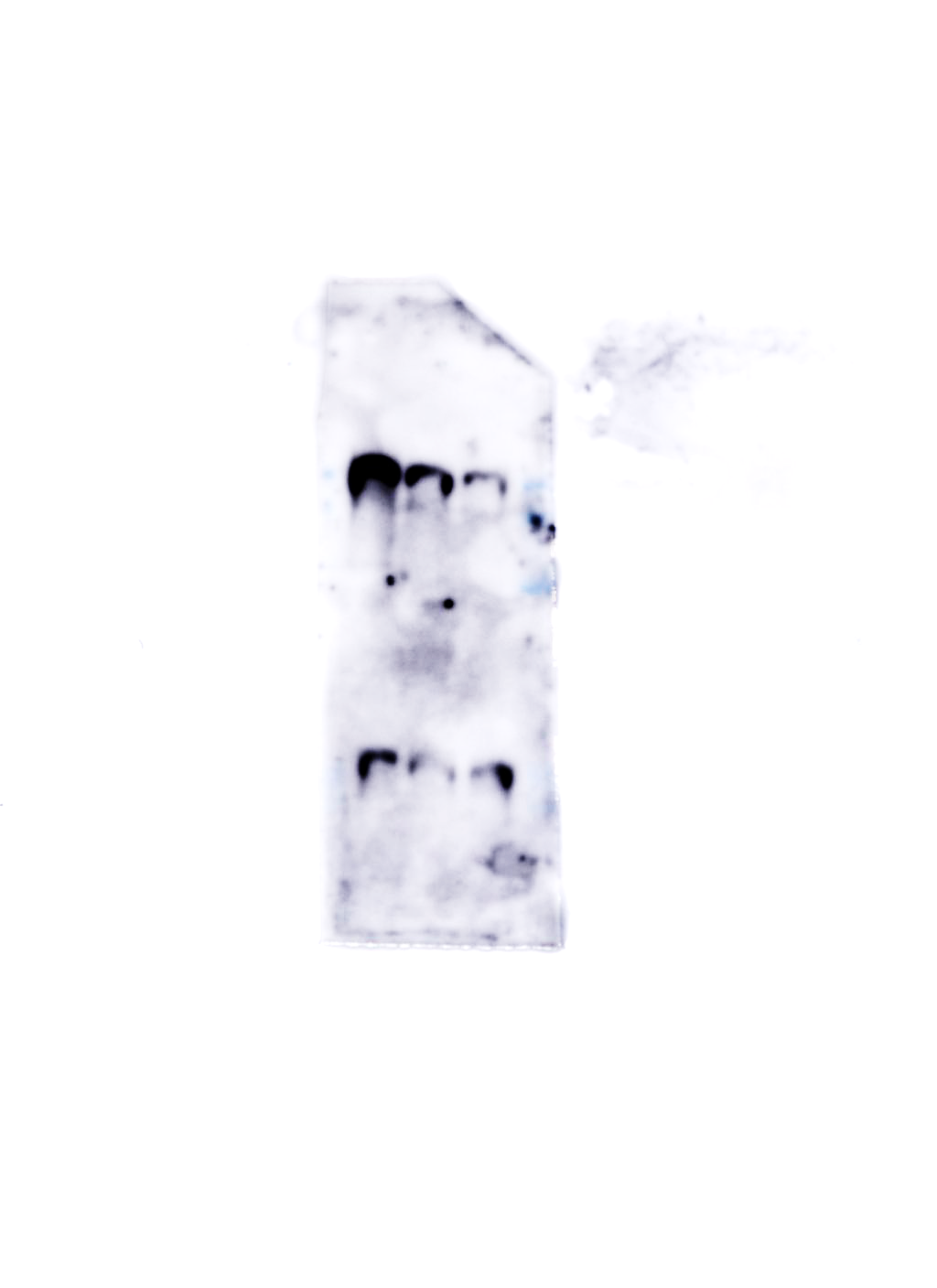

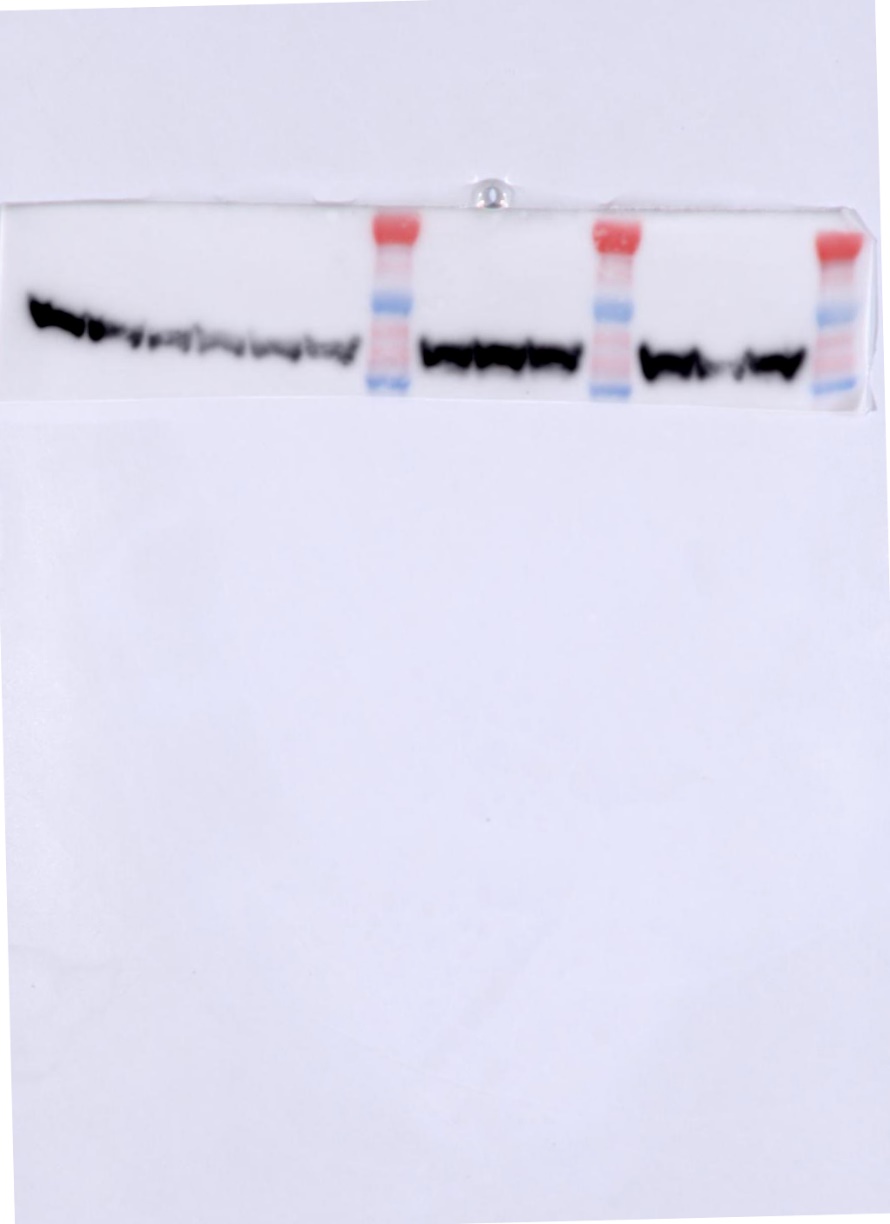


β-ACTIN

Dinaciclib:10nM

0

p-KDM6A

30

60

Time(min)

**Supplementary Fig. 4D**


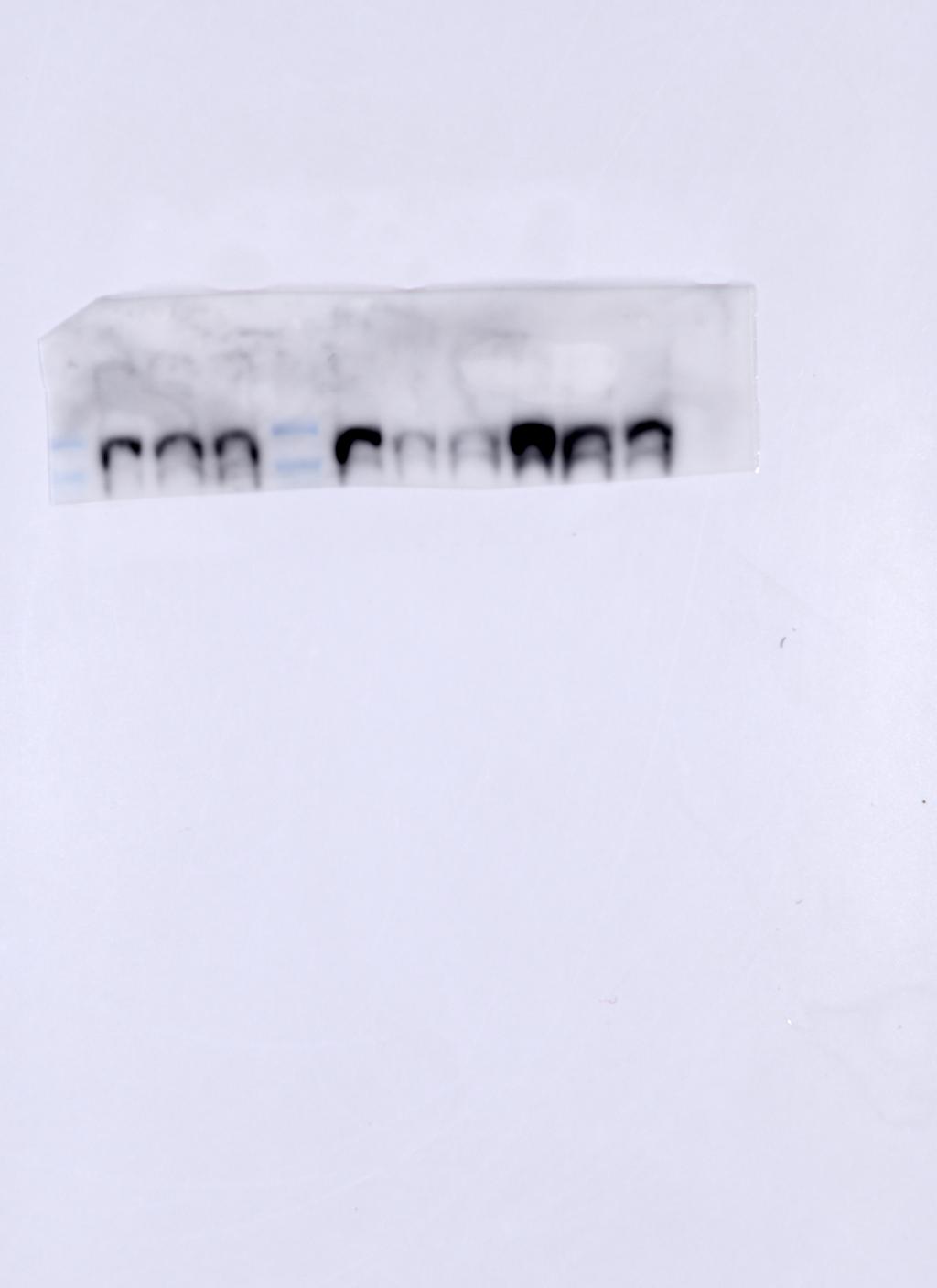


Ro-3306:10nM

Time(min)

60

0

30

p-KDM6A


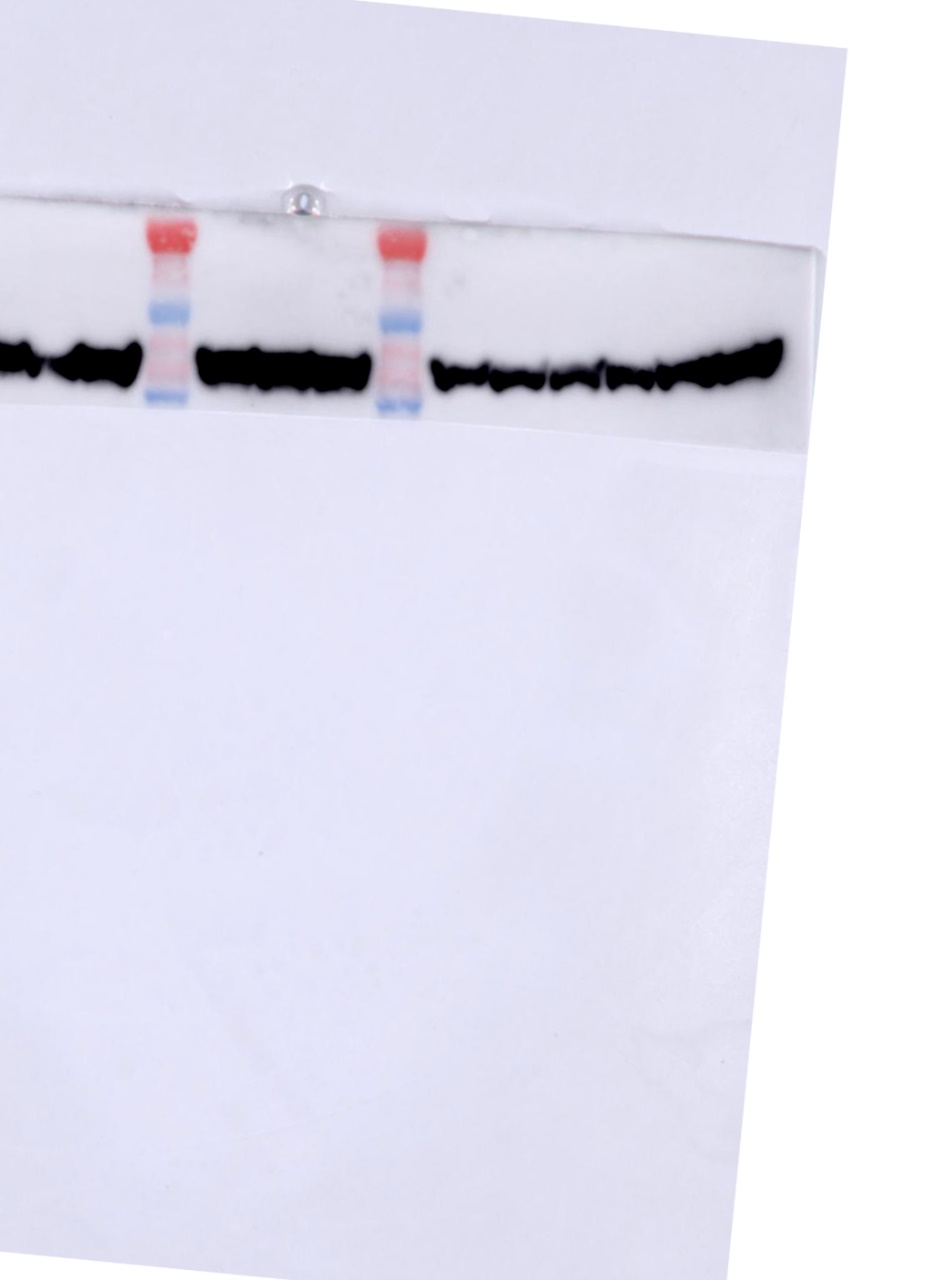


β-ACTIN

**Supplementary Fig. 4F**


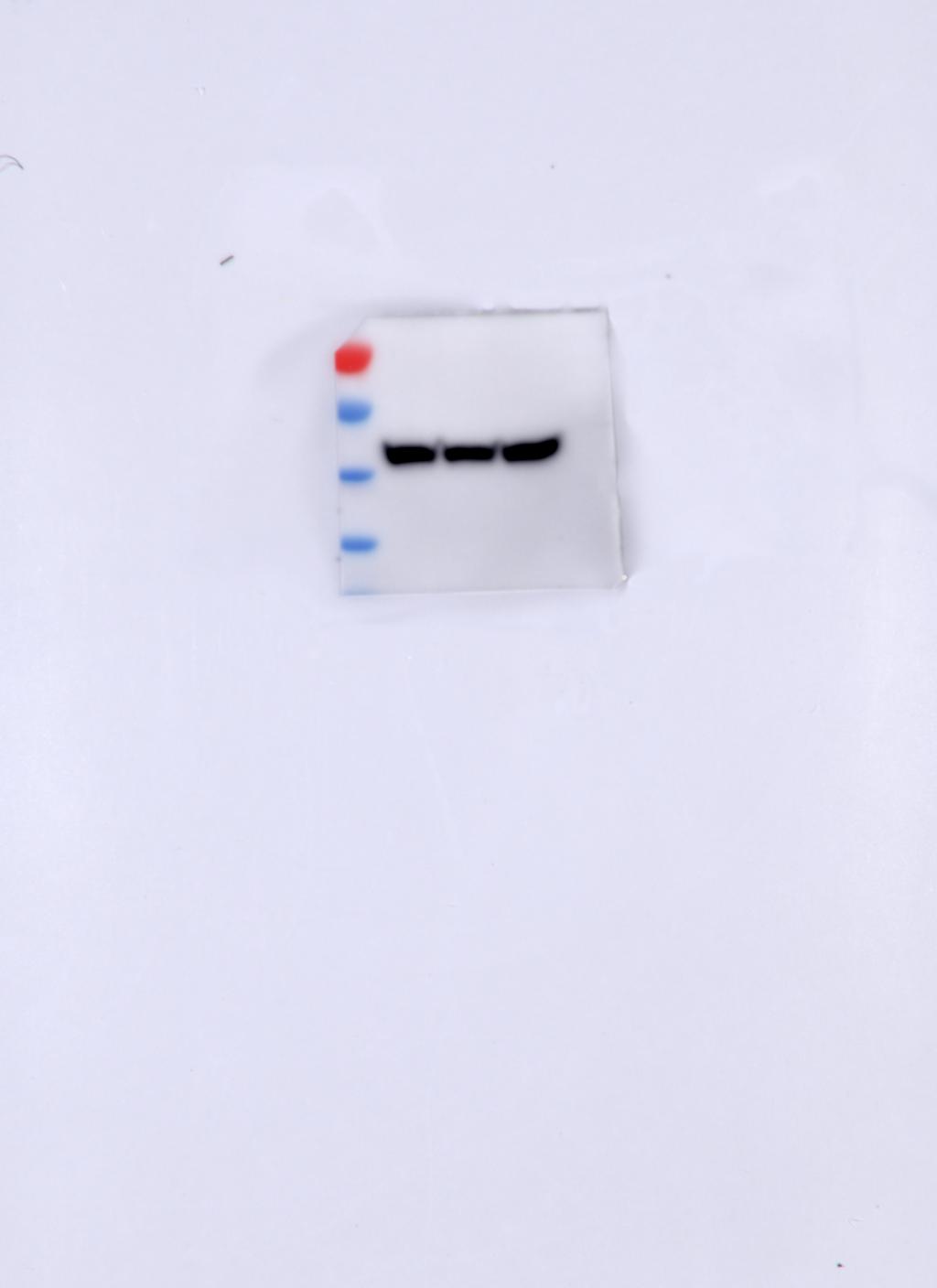

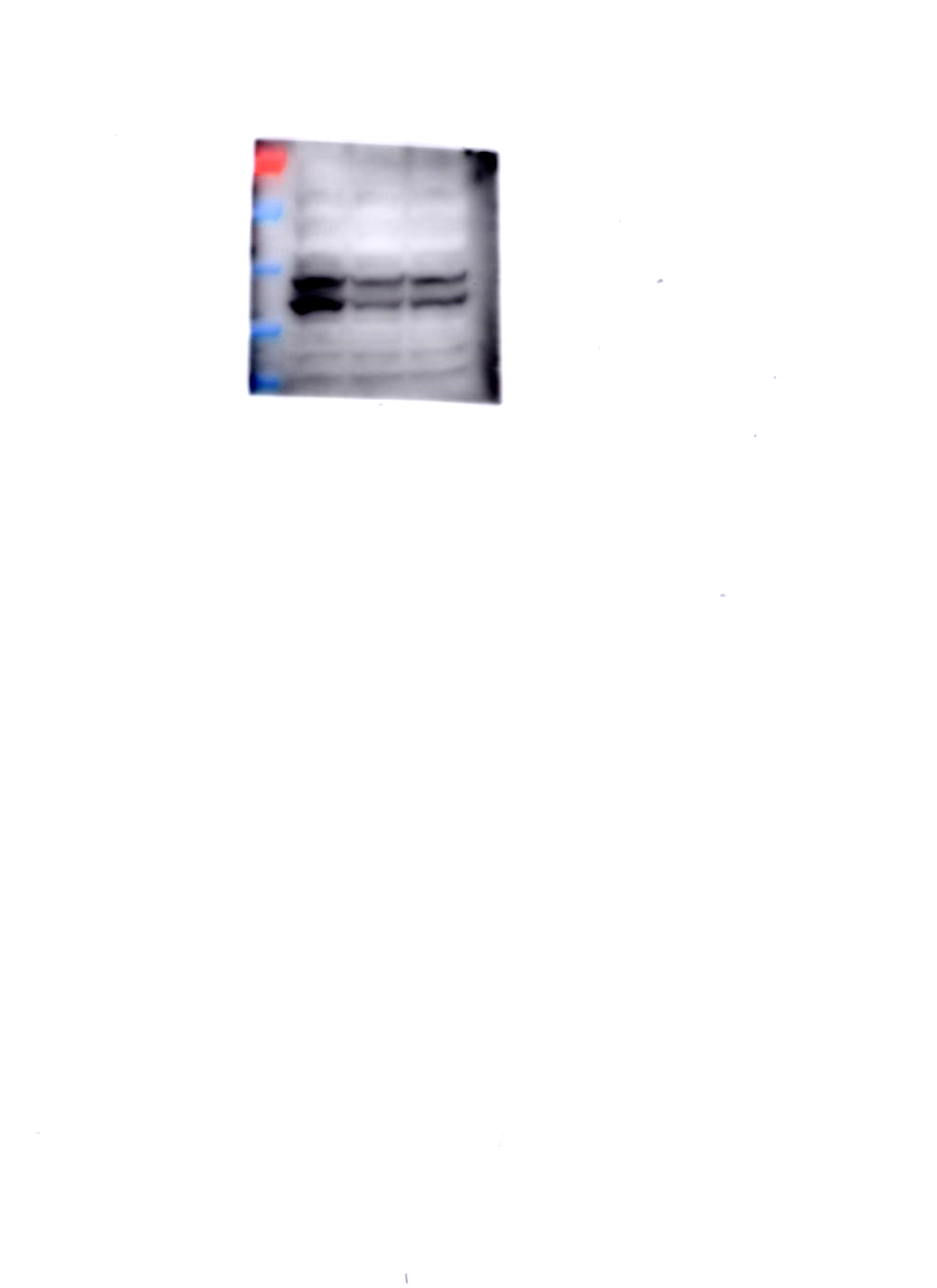

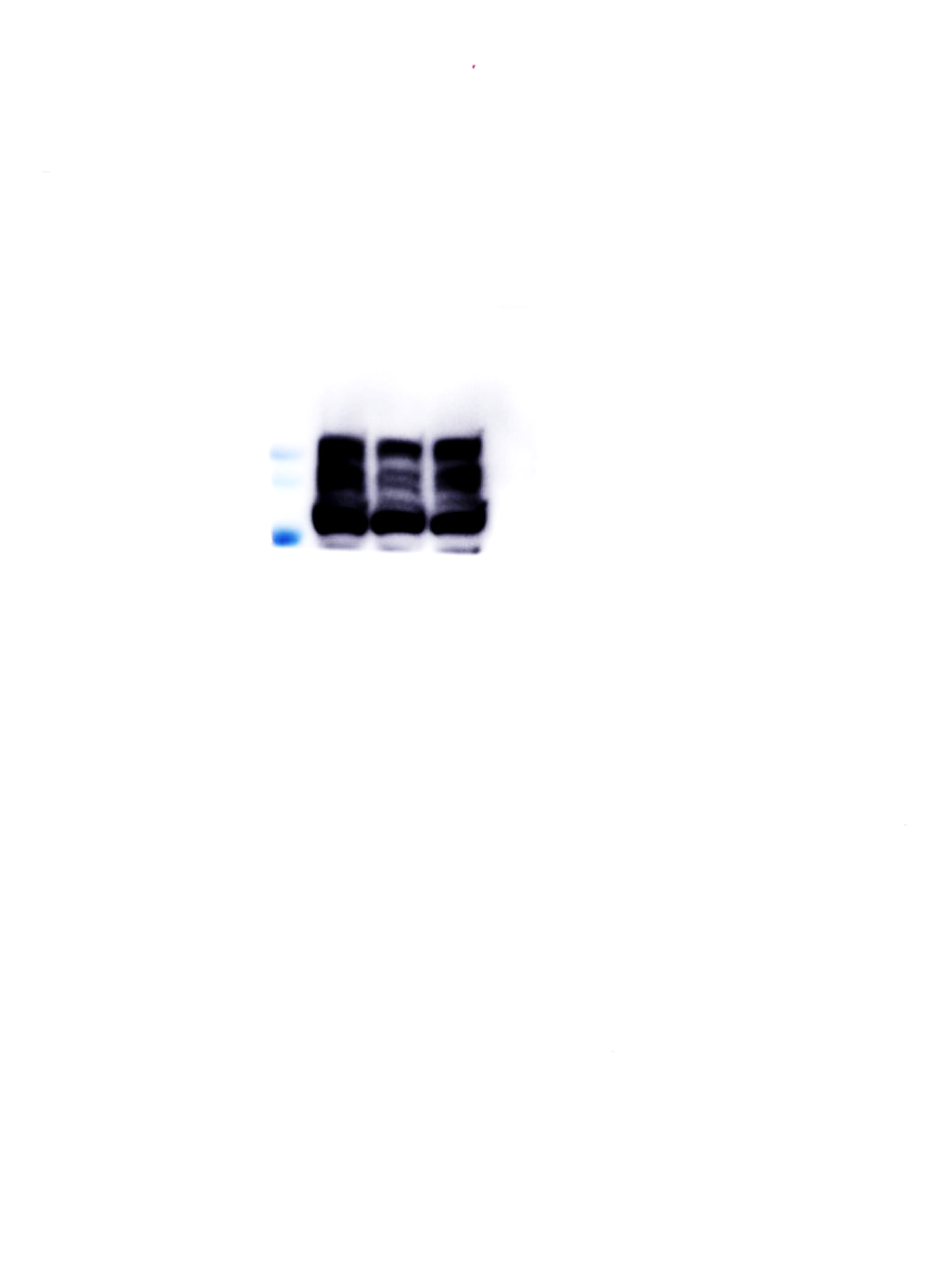

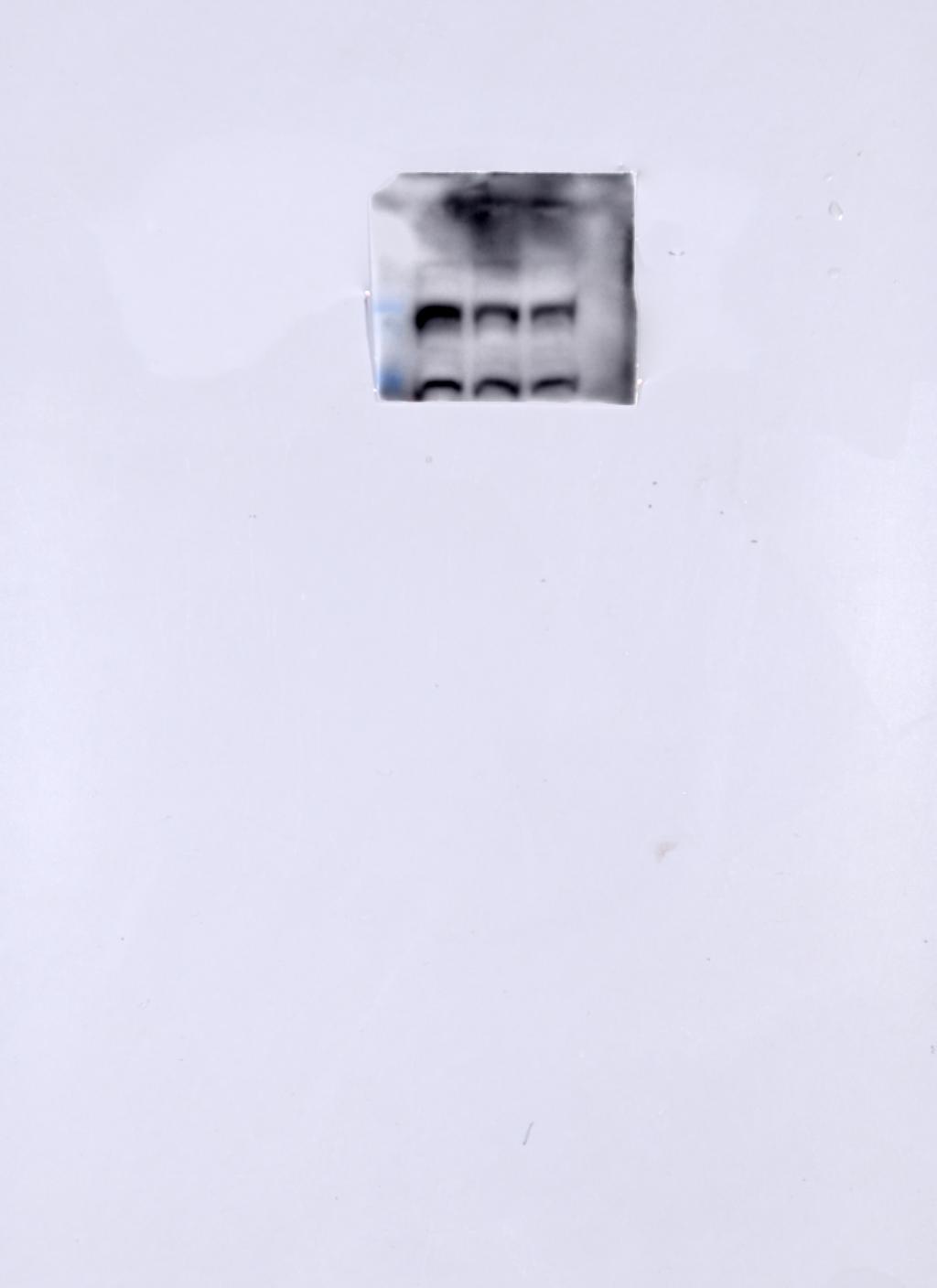


β-ACTIN

CDK1

KDM6A

p-KDM6A

si-CDK1-1

si-CDK1-2

HN6

si-NC
